# Supplementary material for: EGFR transactivates RON to drive oncogenic crosstalk
Source: eLife. 2021 Nov 25;10:e63678. doi: 10.7554/eLife.63678 (PMC8654365; doi:10.7554/eLife.63678)

Figure 1A - Source Data

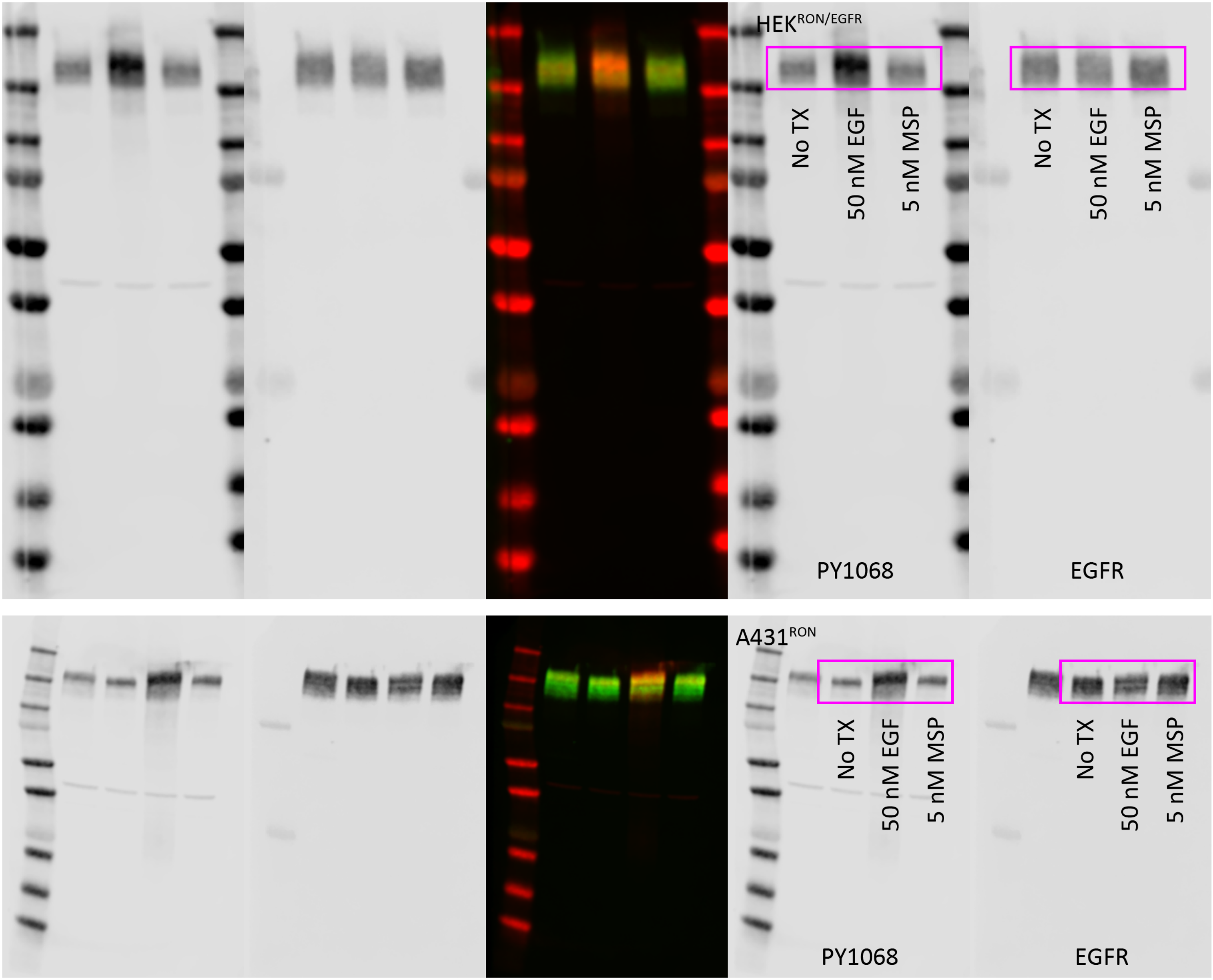

Figure 1B - Source Data

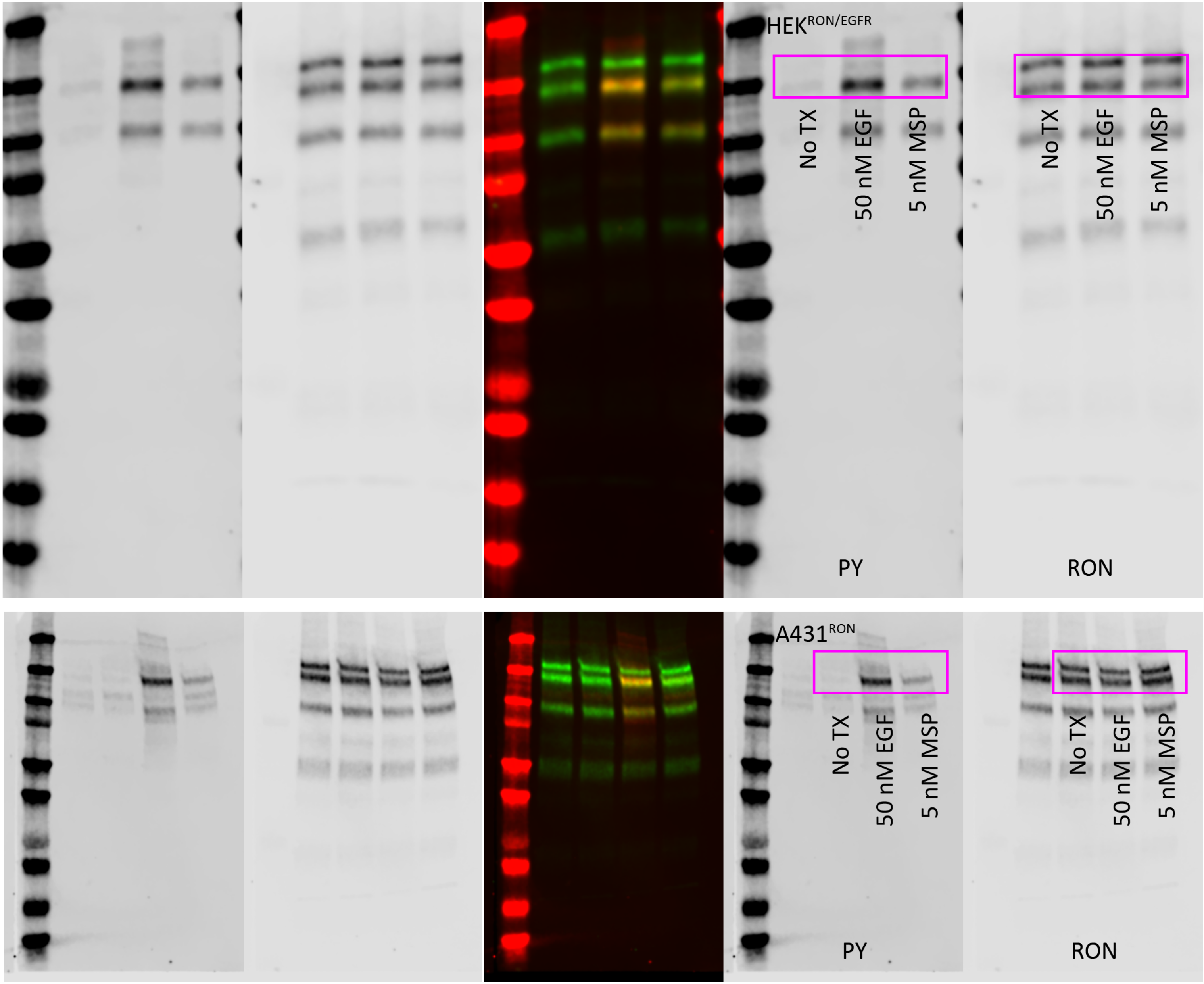

Figure 1C - Source Data

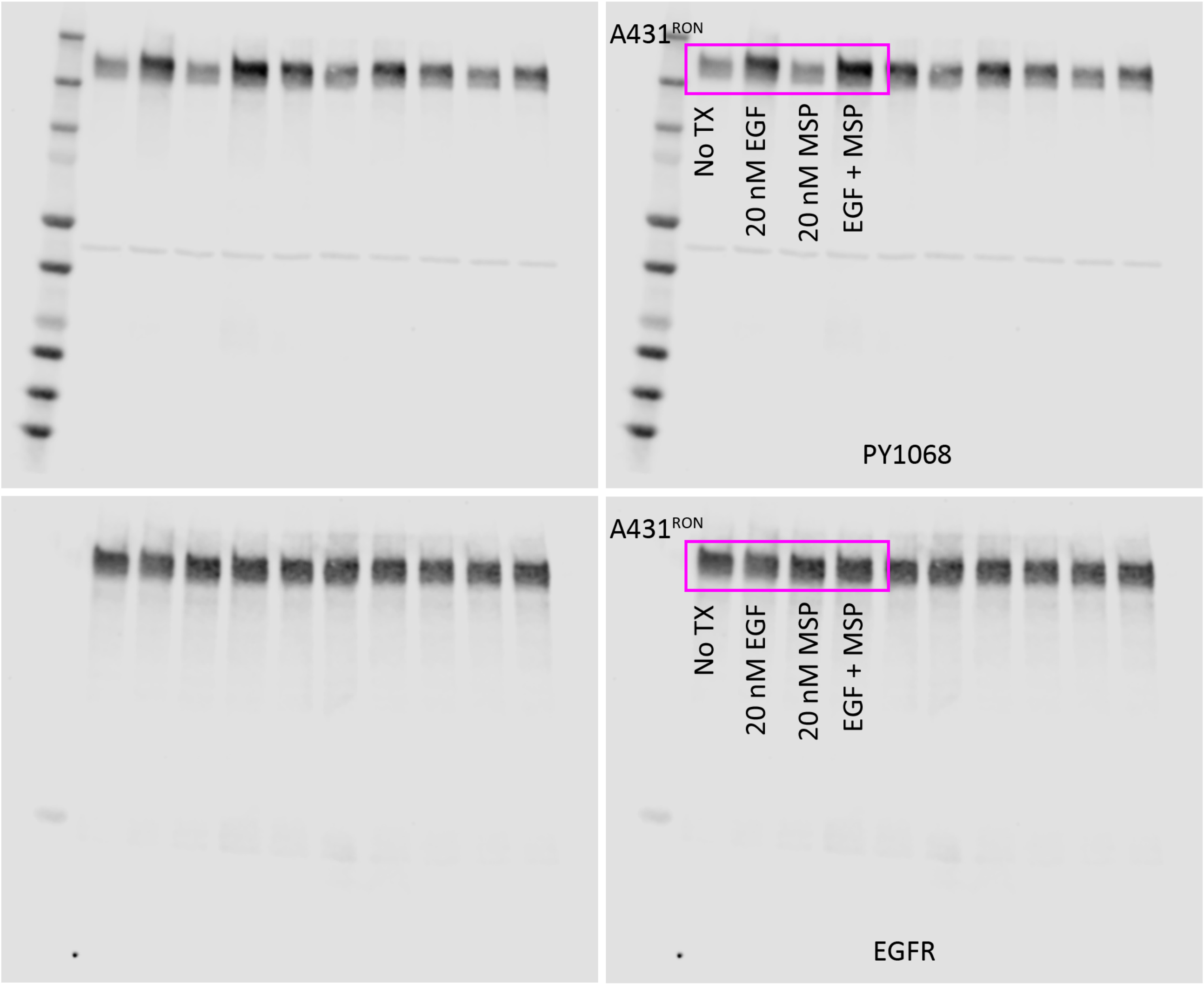

Figure 1D - Source Data

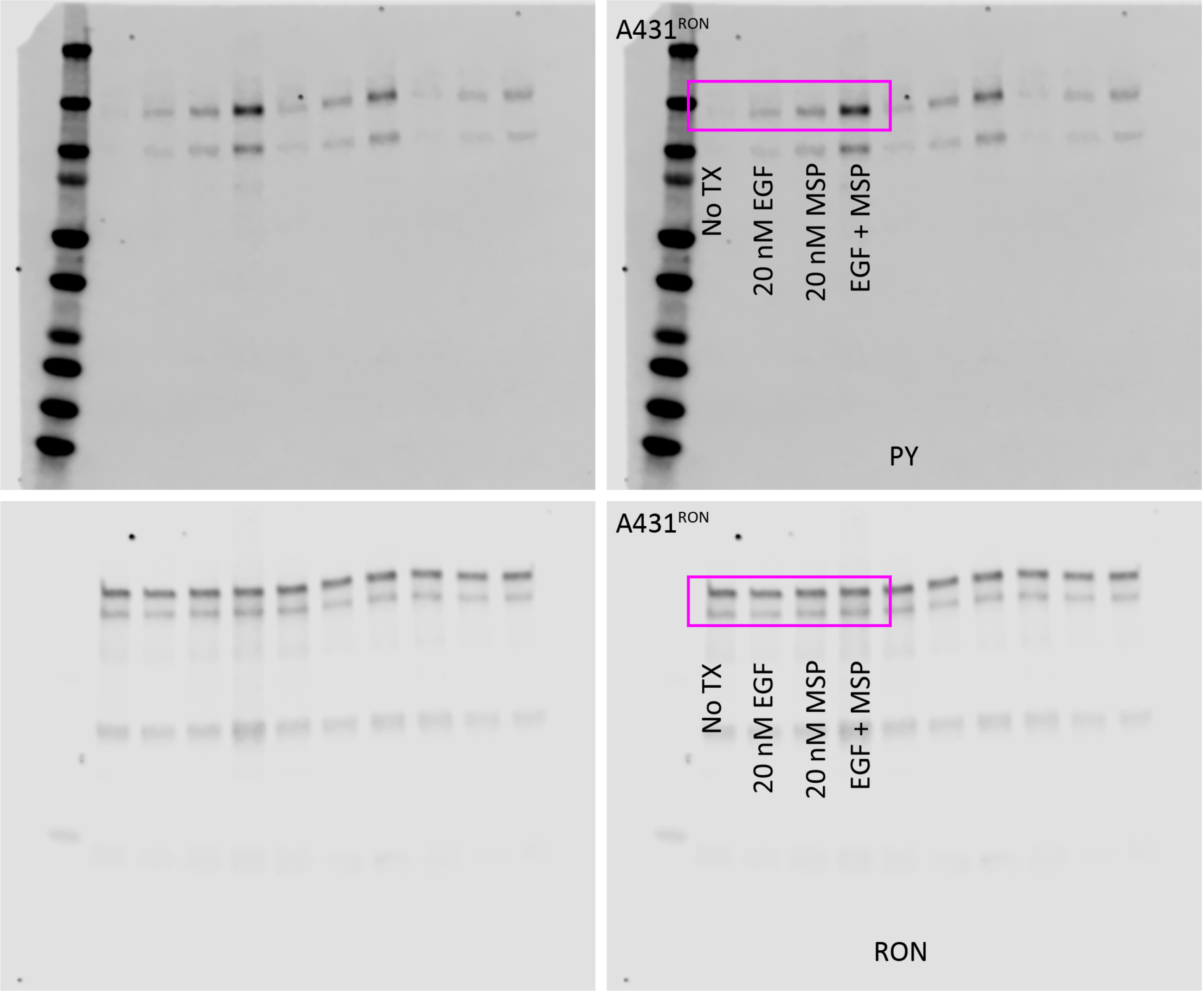

Figure 1E - Source Data

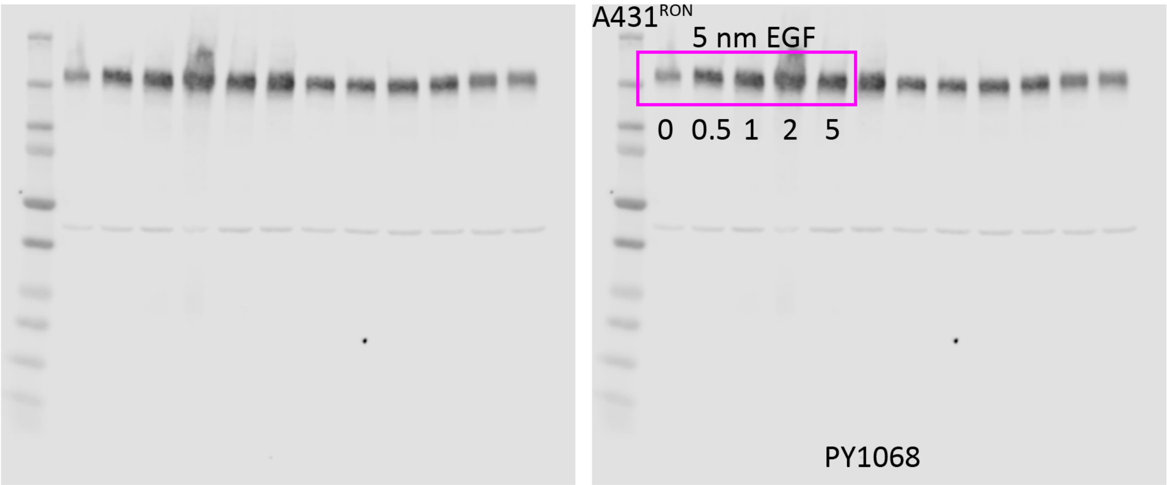

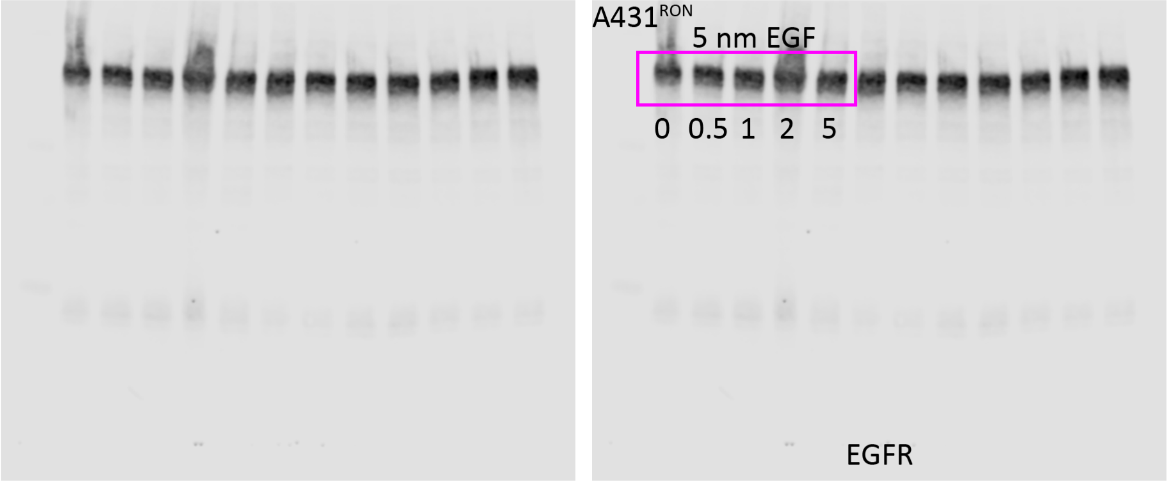

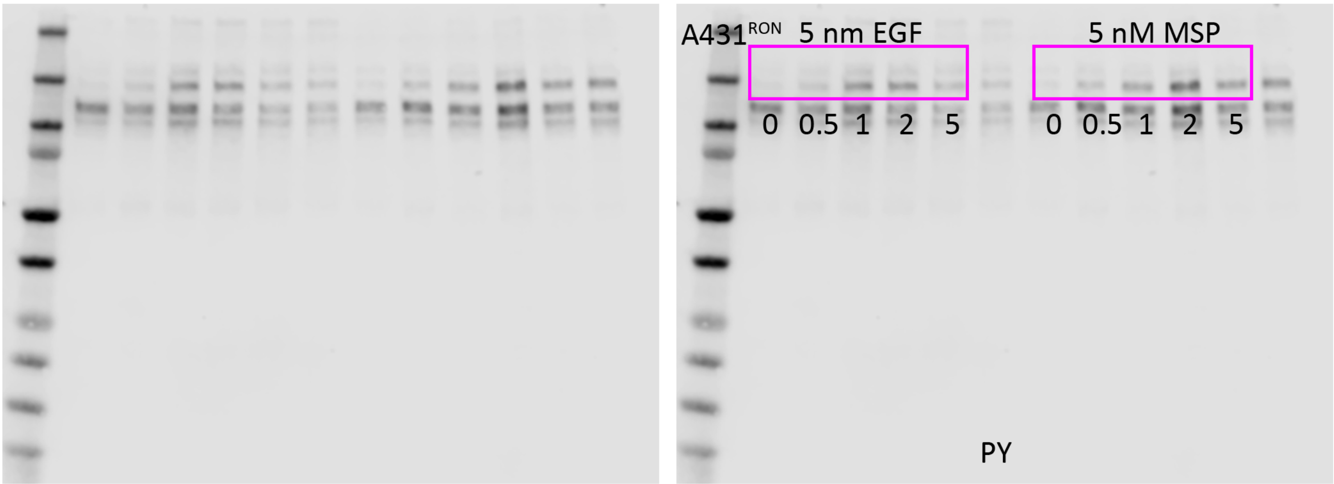

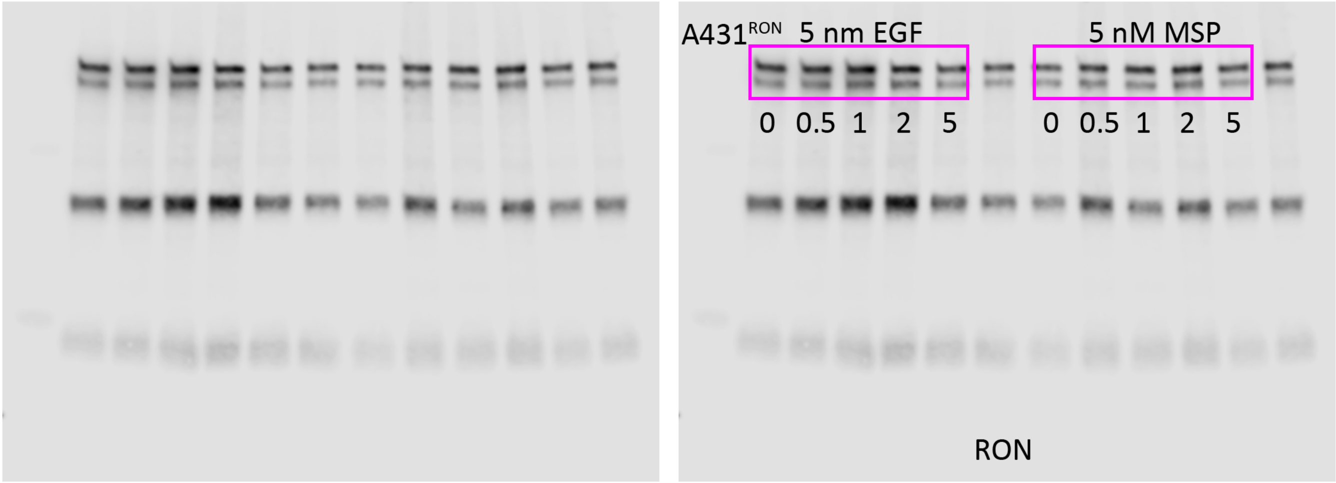

Supplement: Figure 1—source data 1. [file elife-63678-fig1-data1.zip › Figure 1 - Source Data 1/Figure 1 - Source Data 1 - Annotated.pdf]
