# Supplementary figures and images for: EGFR transactivates RON to drive oncogenic crosstalk
Source: eLife. 2021 Nov 25;10:e63678. doi: 10.7554/eLife.63678 (PMC8654365; doi:10.7554/eLife.63678)

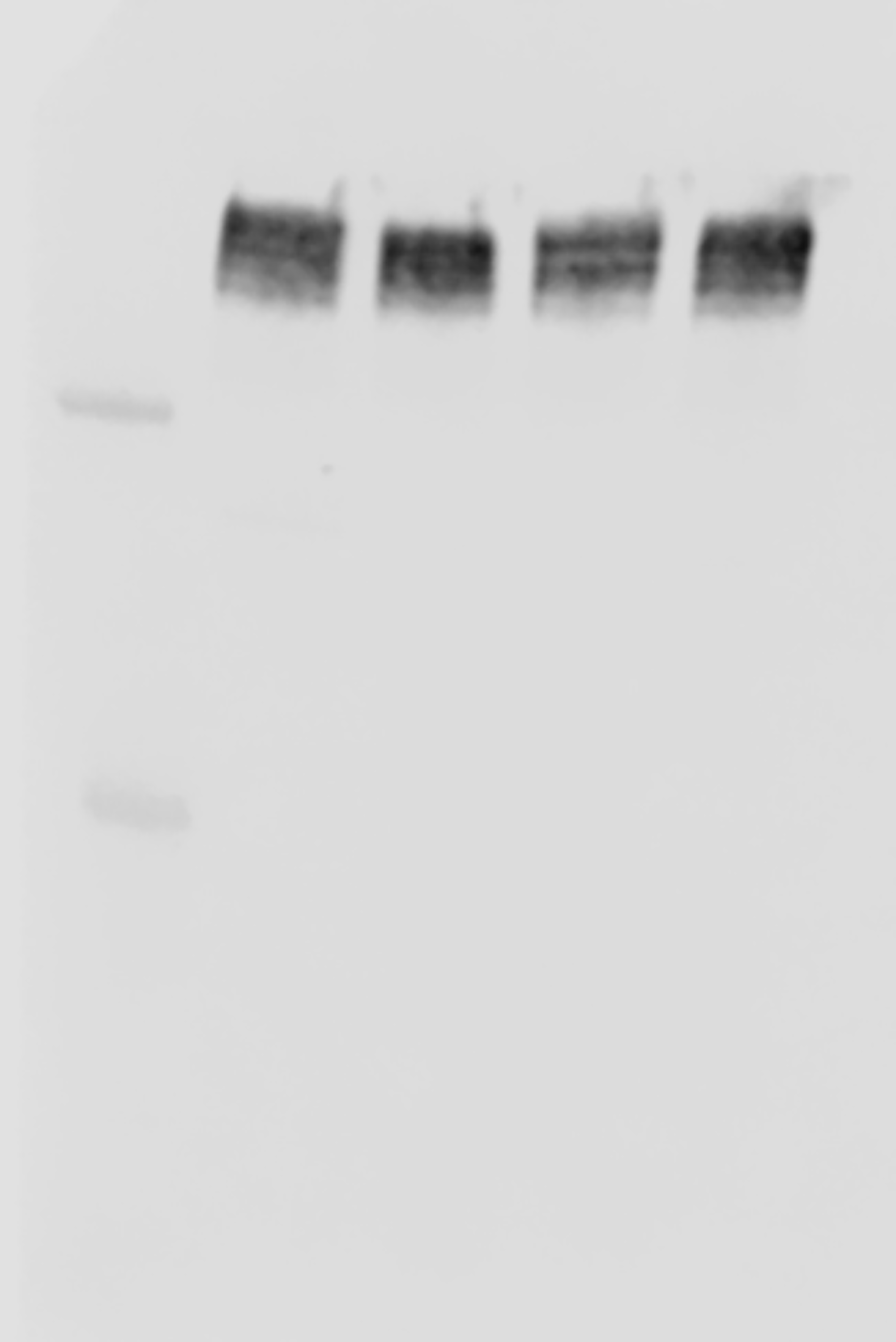

Supplement: Figure 1—source data 1. [file elife-63678-fig1-data1.zip › Figure 1 - Source Data 1/Fig1A - A431 - EGFR - Protein.tif]

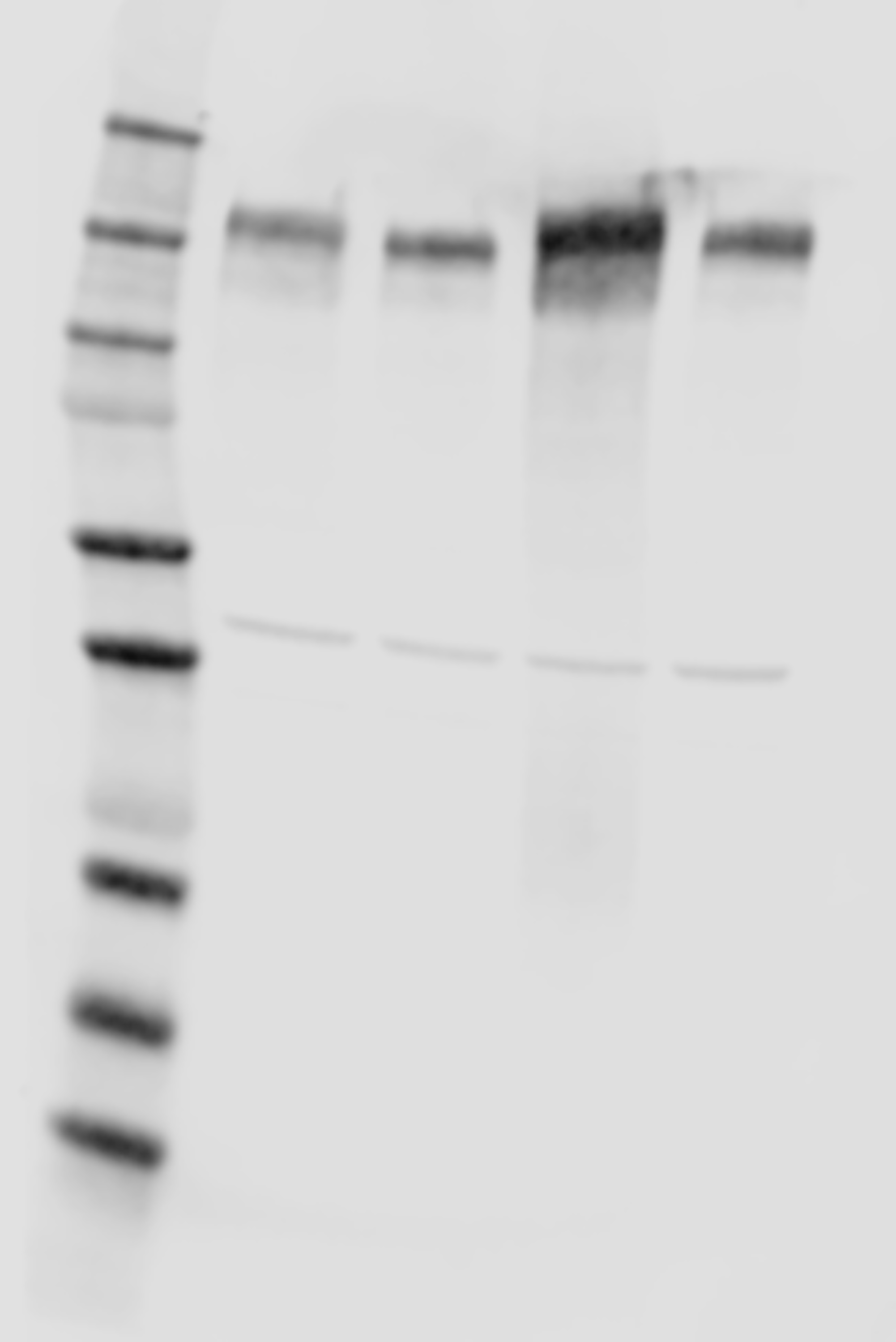

Supplement: Figure 1—source data 1. [file elife-63678-fig1-data1.zip › Figure 1 - Source Data 1/Fig1A - A431 - EGFR - PY.tif]

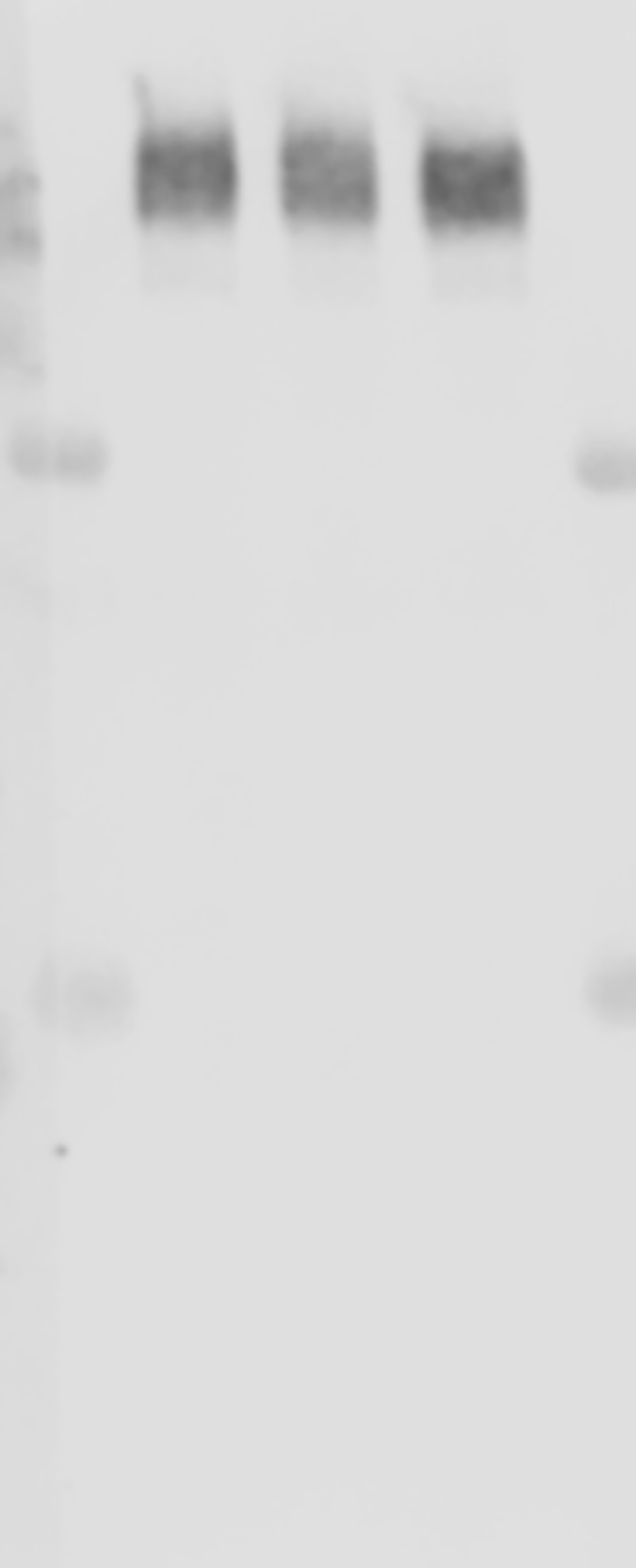

Supplement: Figure 1—source data 1. [file elife-63678-fig1-data1.zip › Figure 1 - Source Data 1/Fig1A - HEK - EGFR - Protein.tif]

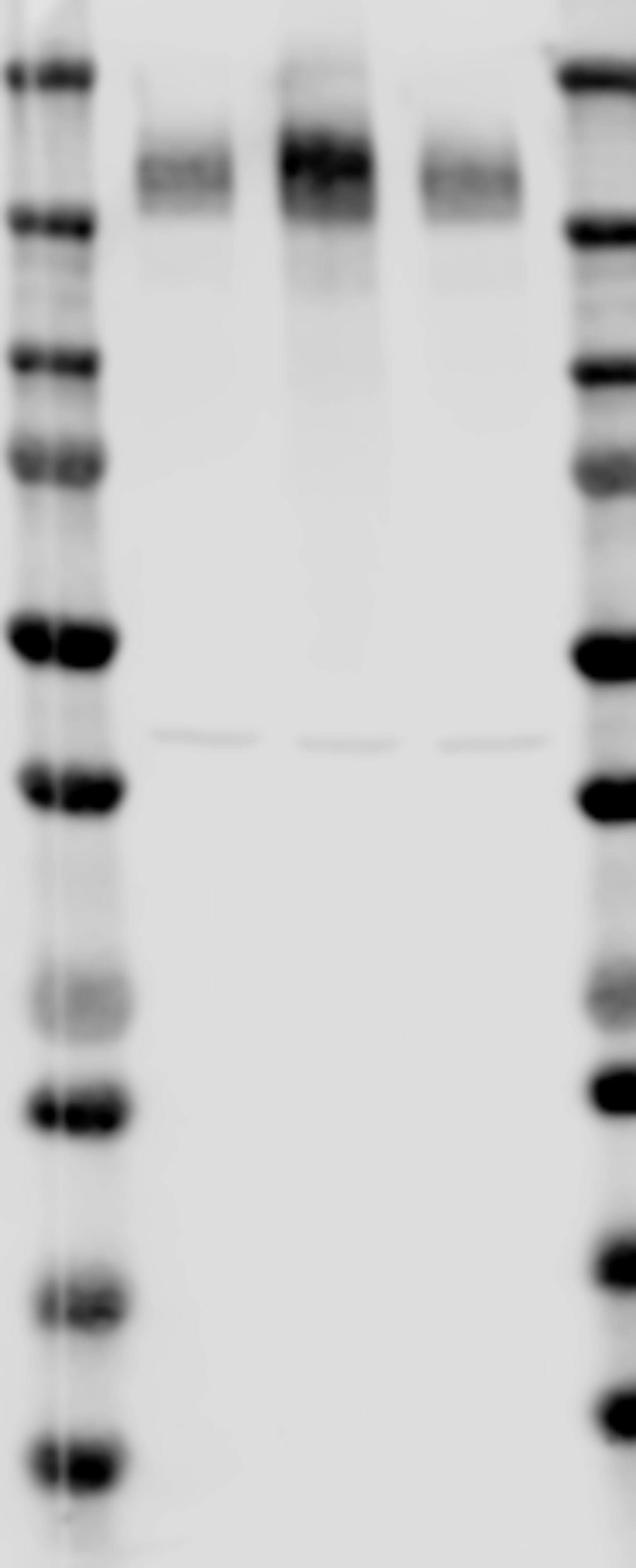

Supplement: Figure 1—source data 1. [file elife-63678-fig1-data1.zip › Figure 1 - Source Data 1/Fig1A - HEK - EGFR - PY.tif]

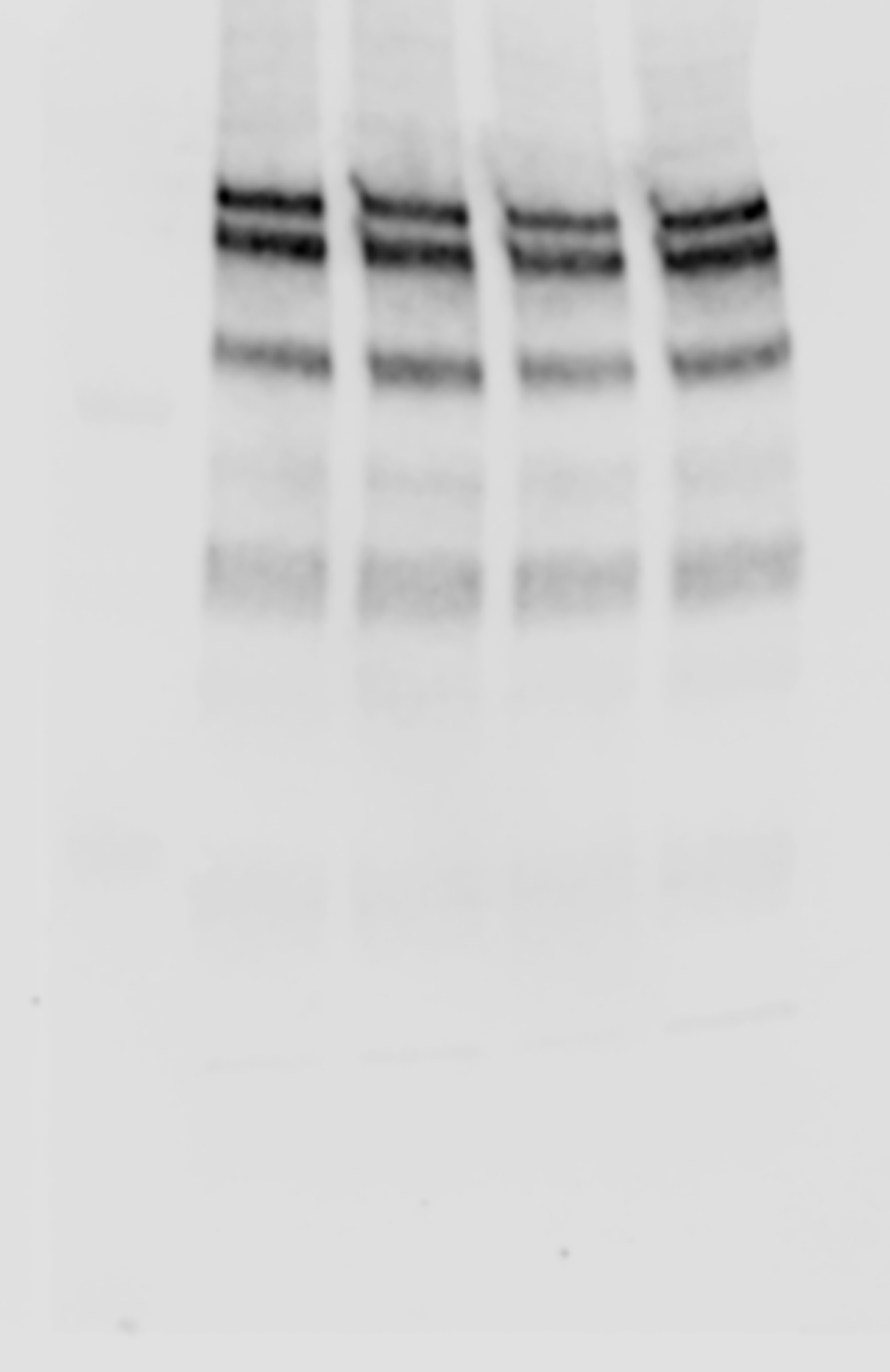

Supplement: Figure 1—source data 1. [file elife-63678-fig1-data1.zip › Figure 1 - Source Data 1/Fig1B - A431 - RON IP - Protein.tif]

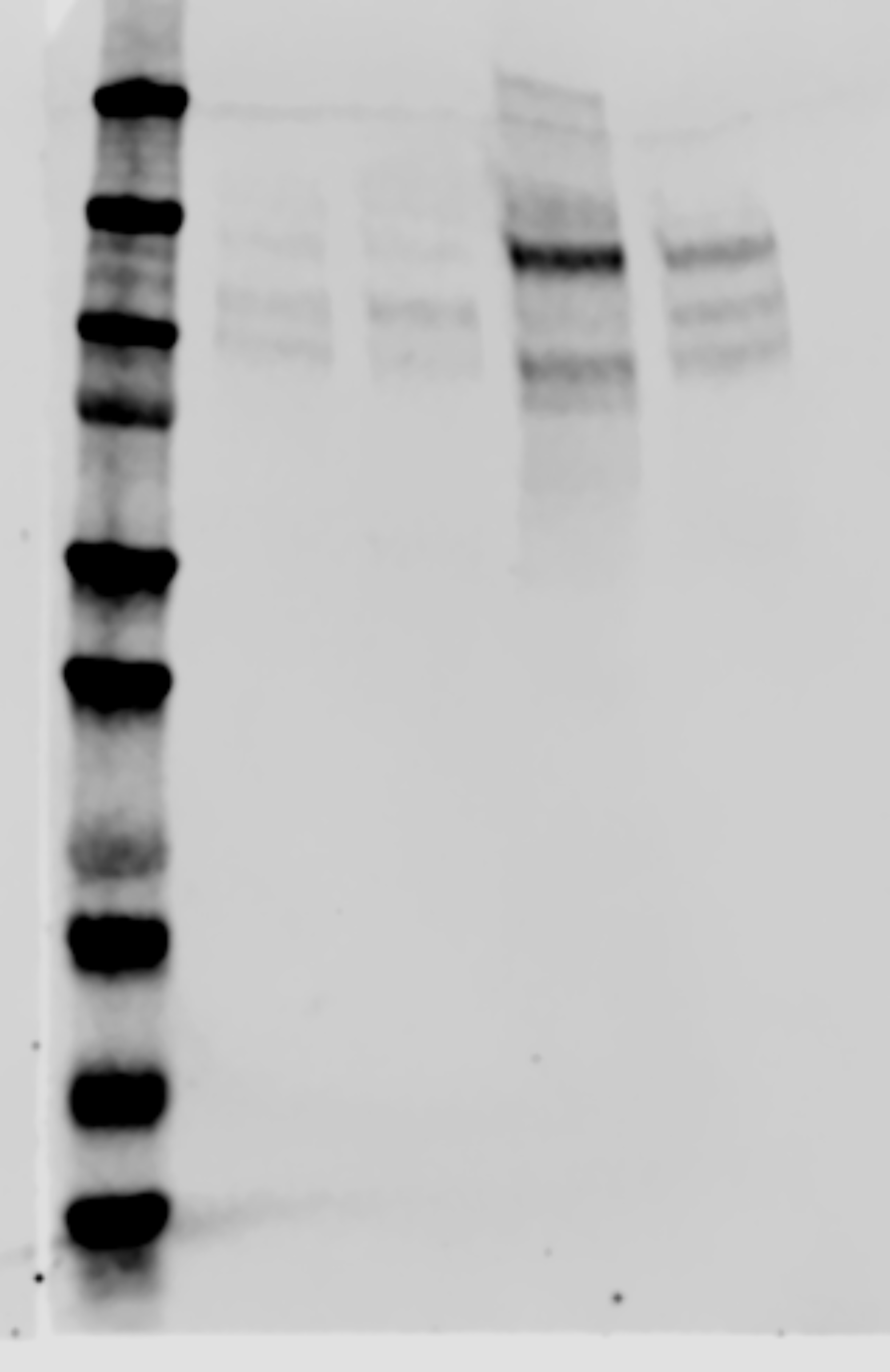

Supplement: Figure 1—source data 1. [file elife-63678-fig1-data1.zip › Figure 1 - Source Data 1/Fig1B - A431 - RON IP - PY.tif]

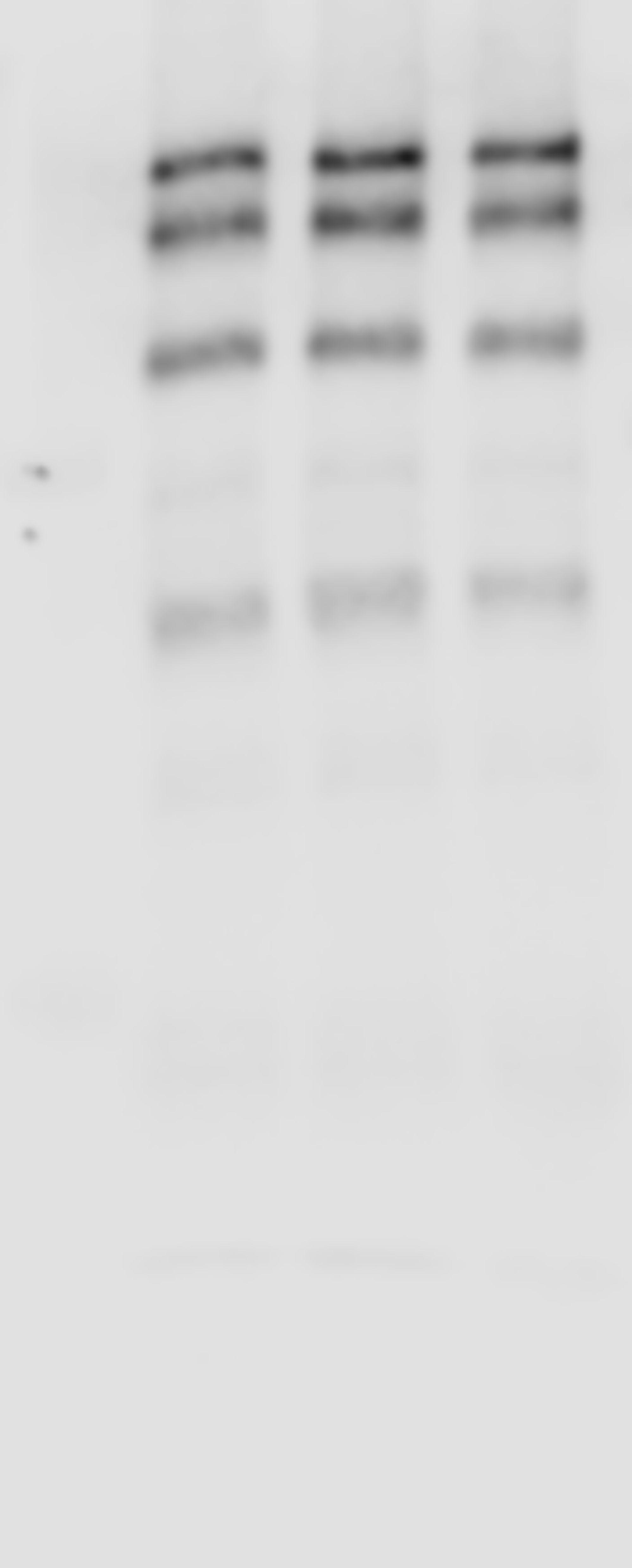

Supplement: Figure 1—source data 1. [file elife-63678-fig1-data1.zip › Figure 1 - Source Data 1/Fig1B - HEK - RON IP - Protein.tif]

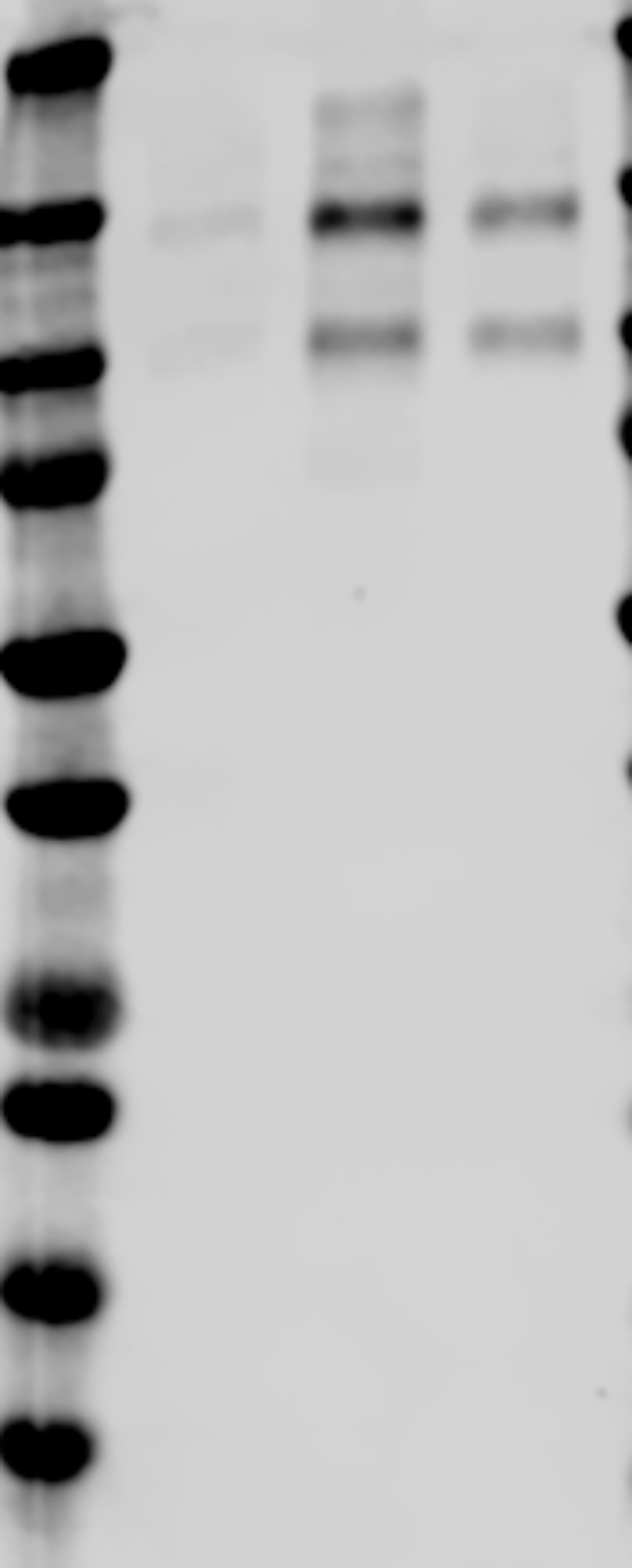

Supplement: Figure 1—source data 1. [file elife-63678-fig1-data1.zip › Figure 1 - Source Data 1/Fig1B - HEK - RON IP - PY.tif]

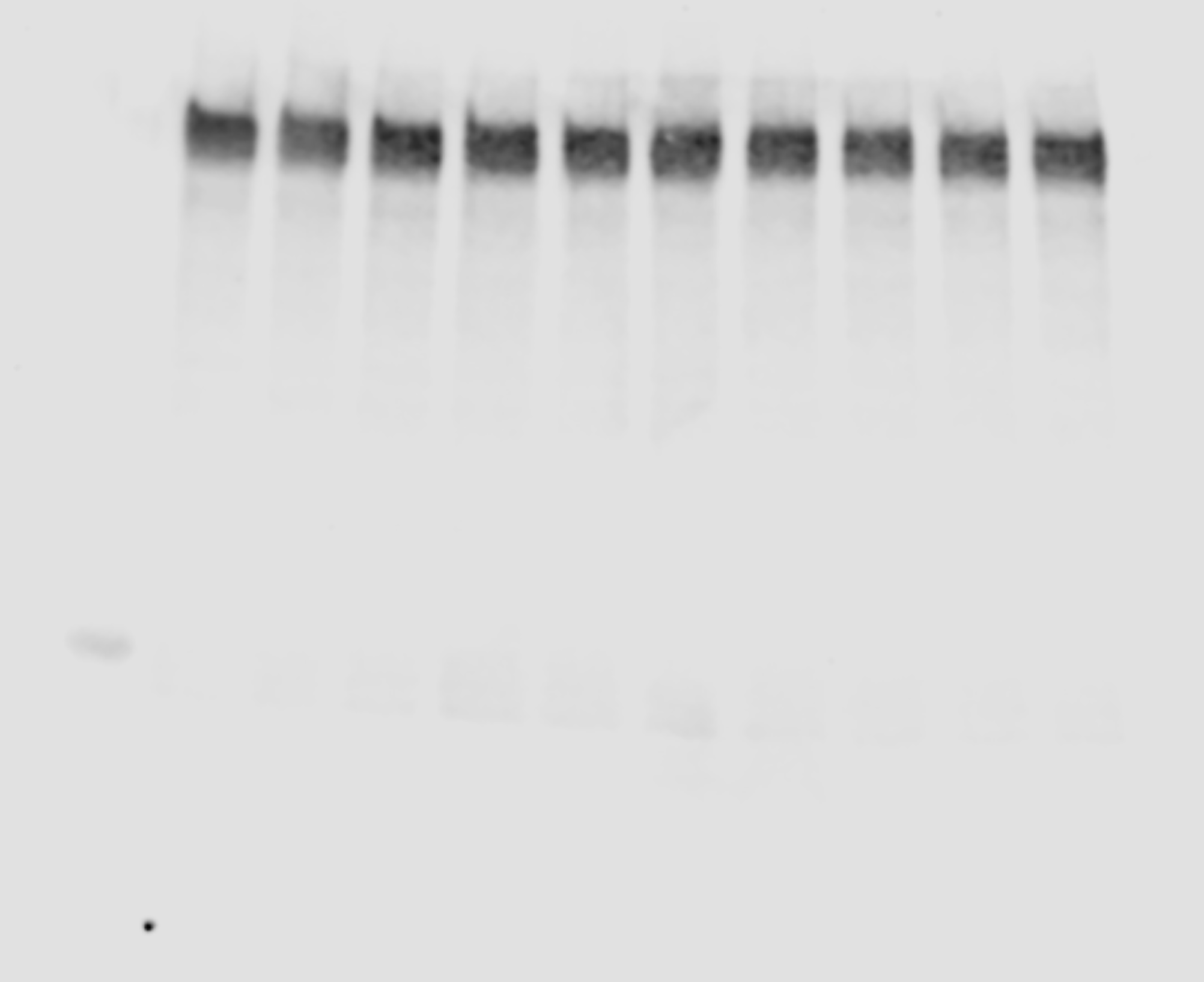

Supplement: Figure 1—source data 1. [file elife-63678-fig1-data1.zip › Figure 1 - Source Data 1/Fig1C - EGFR - Protein.tif]

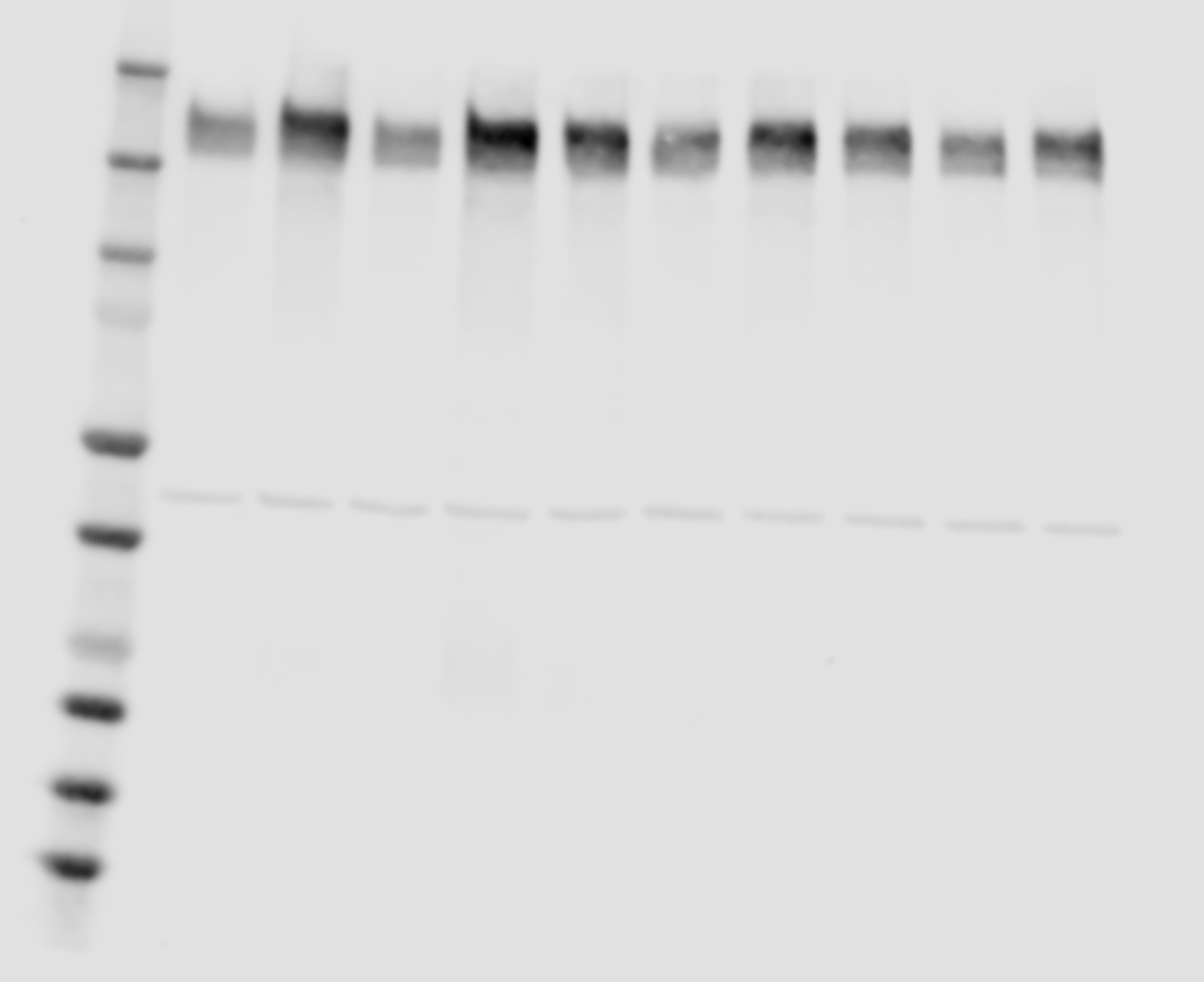

Supplement: Figure 1—source data 1. [file elife-63678-fig1-data1.zip › Figure 1 - Source Data 1/Fig1C - EGFR - PY.tif]

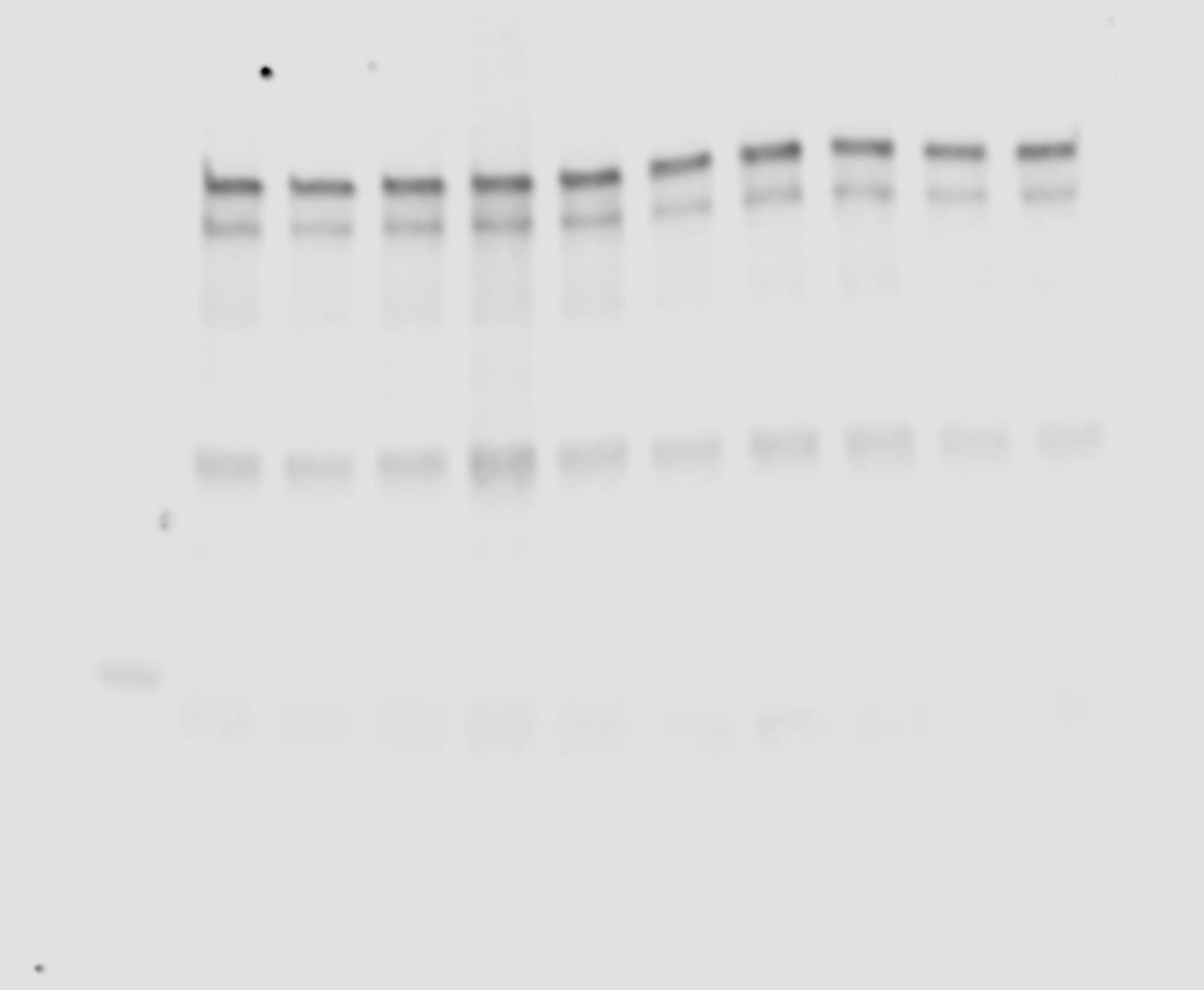

Supplement: Figure 1—source data 1. [file elife-63678-fig1-data1.zip › Figure 1 - Source Data 1/Fig1D - RON IP - Protein.tif]

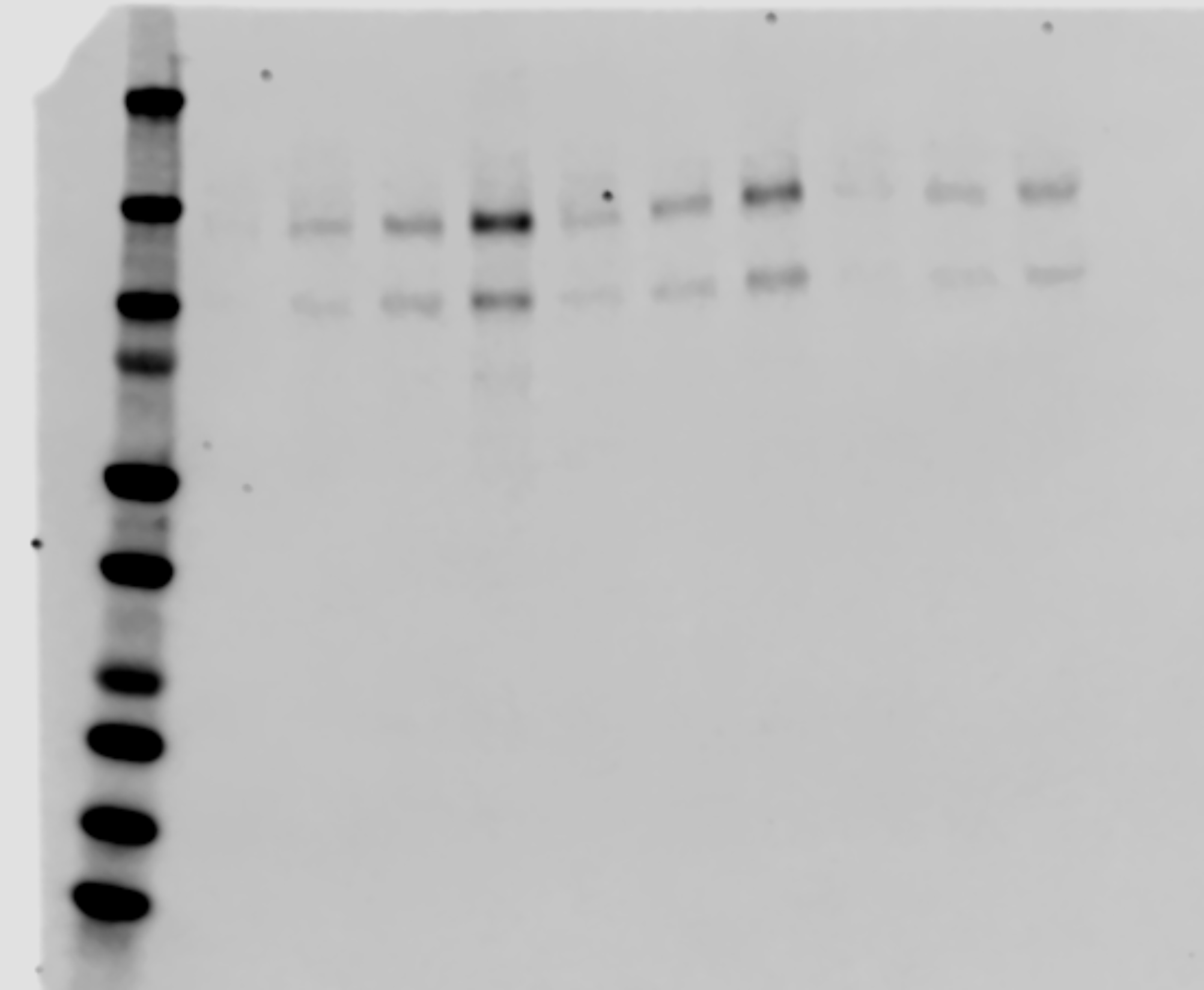

Supplement: Figure 1—source data 1. [file elife-63678-fig1-data1.zip › Figure 1 - Source Data 1/Fig1D - RON IP - PY.tif]

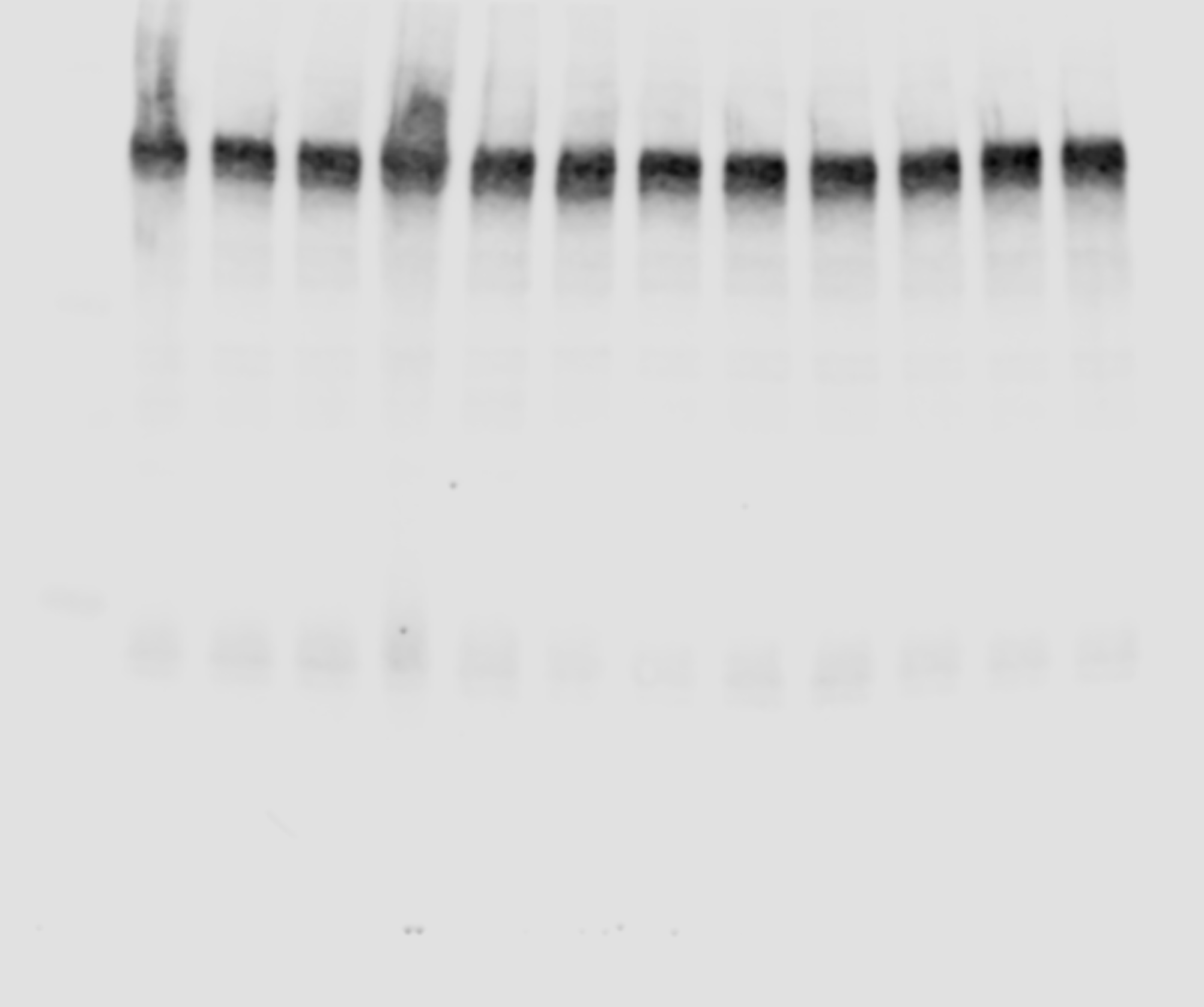

Supplement: Figure 1—source data 1. [file elife-63678-fig1-data1.zip › Figure 1 - Source Data 1/Fig1E - EGFR - Protein.tif]

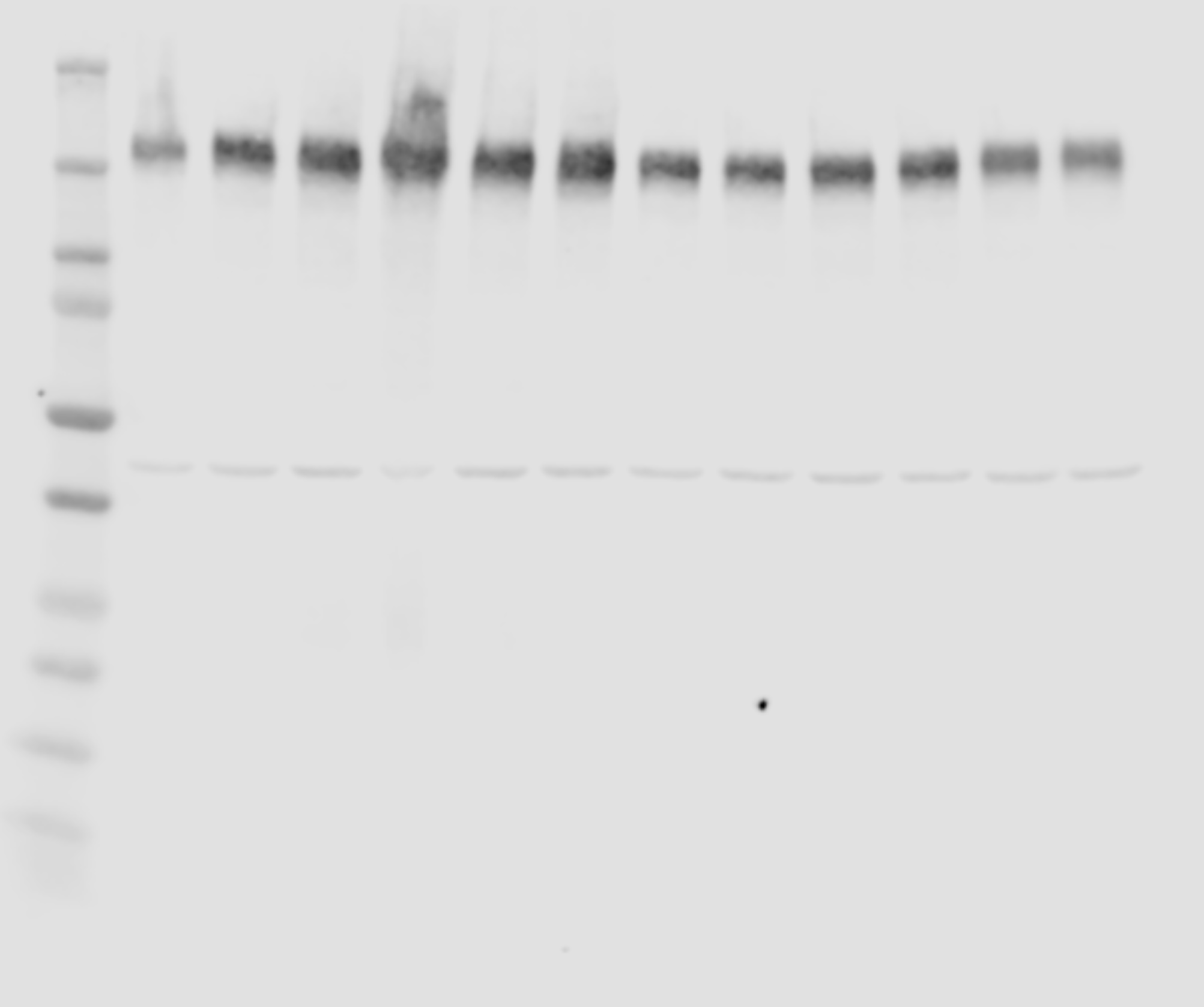

Supplement: Figure 1—source data 1. [file elife-63678-fig1-data1.zip › Figure 1 - Source Data 1/Fig1E - EGFR - PY.tif]

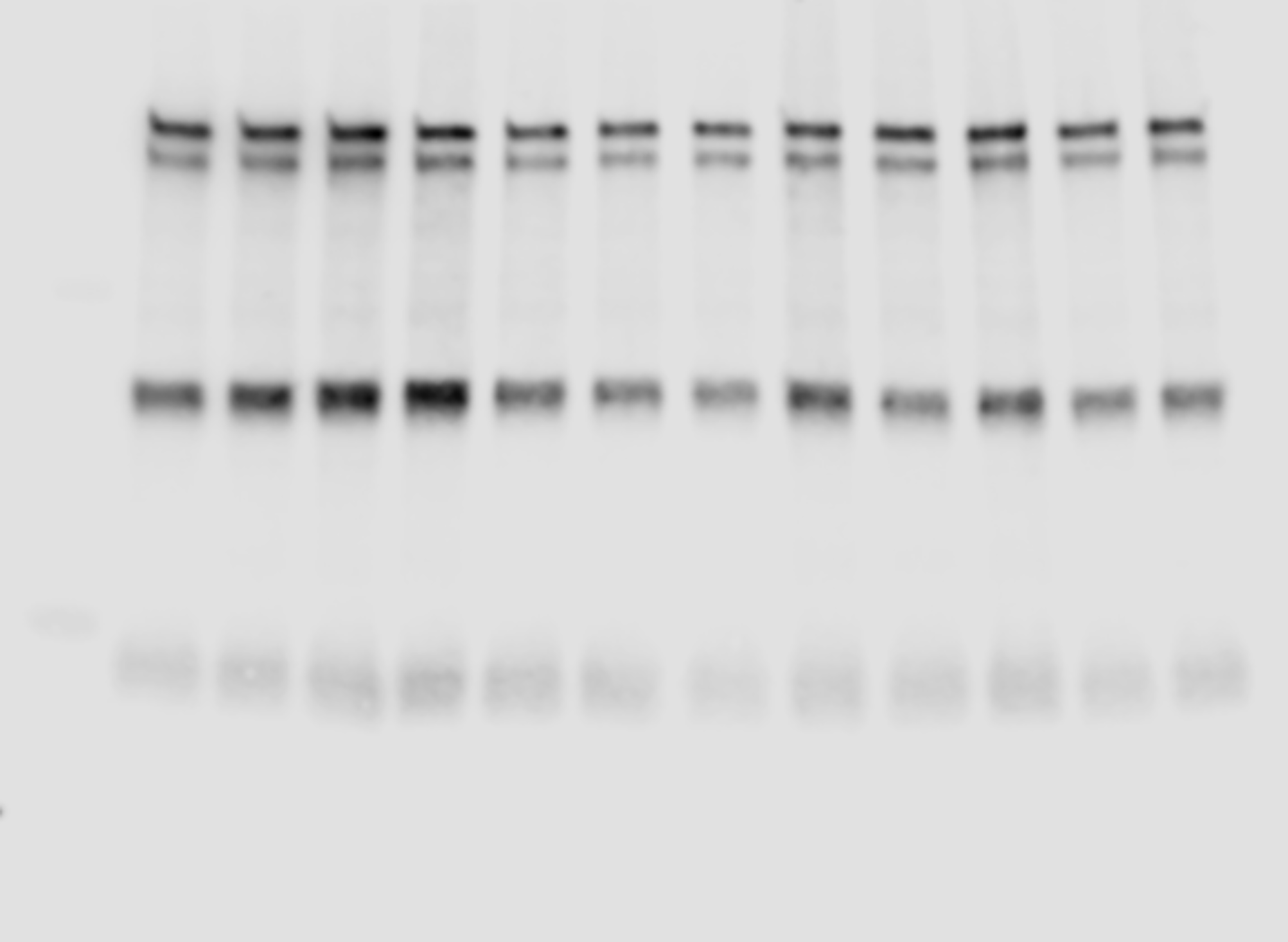

Supplement: Figure 1—source data 1. [file elife-63678-fig1-data1.zip › Figure 1 - Source Data 1/Fig1E - IP RON - Protein.tif]

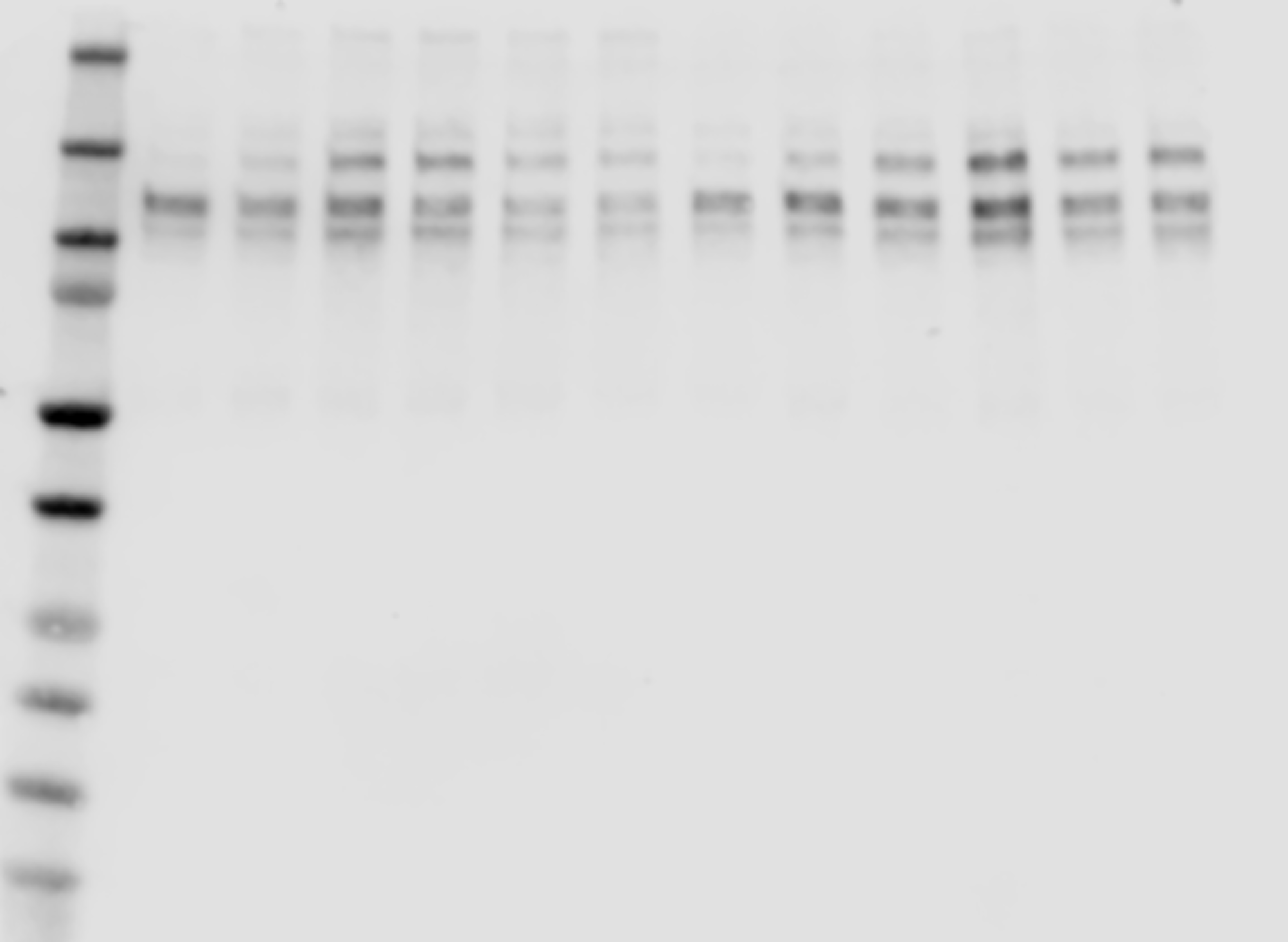

Supplement: Figure 1—source data 1. [file elife-63678-fig1-data1.zip › Figure 1 - Source Data 1/Fig1E - IP RON - PY.tif]

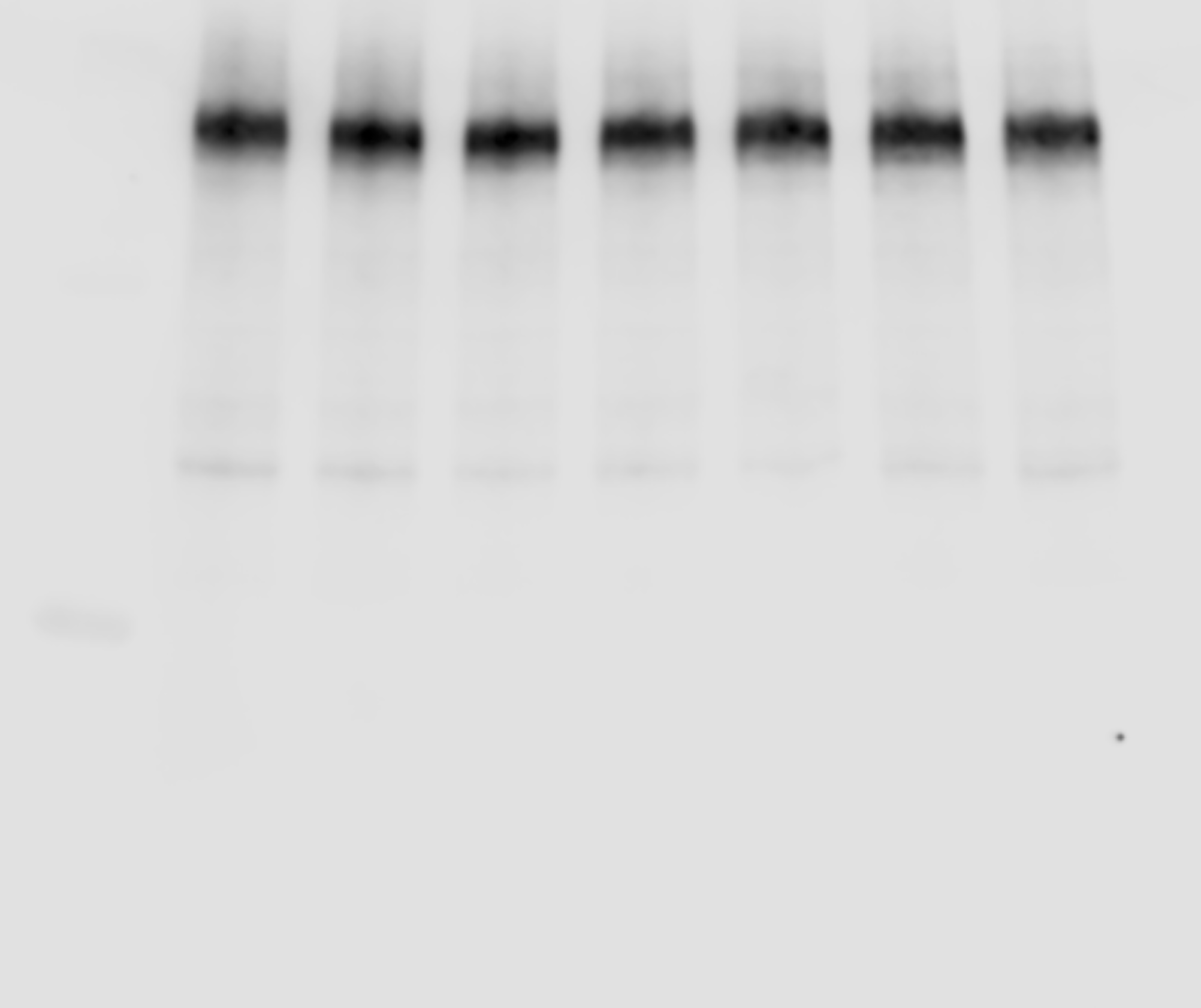

Supplement: Figure 1—figure supplement 1—source data 1. [file elife-63678-fig1-figsupp1-data1.zip › Figure 1 - Figure Supplement 1 - Source Data 1/Fig1Sup1 - EGFR - Protein.tif]

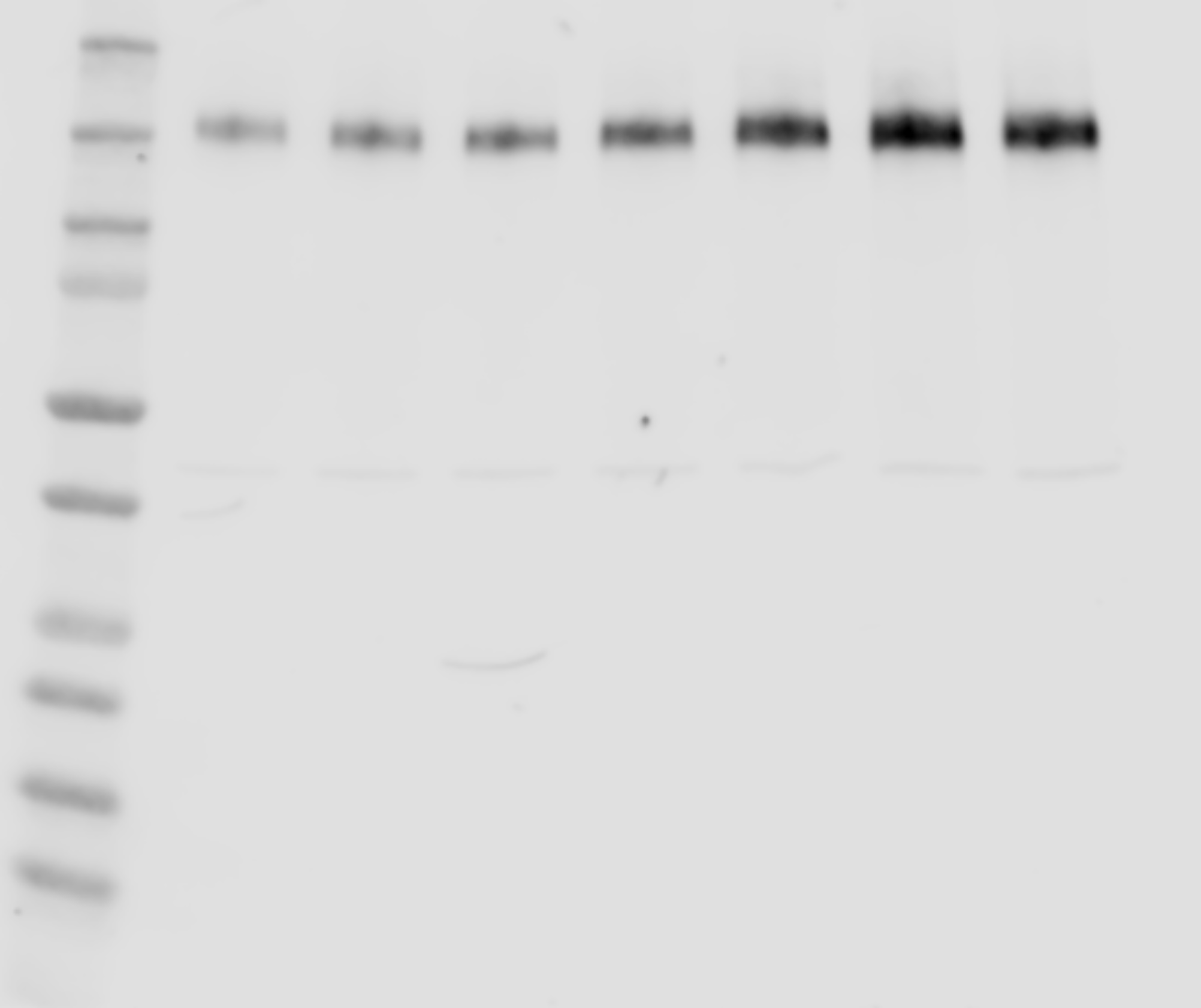

Supplement: Figure 1—figure supplement 1—source data 1. [file elife-63678-fig1-figsupp1-data1.zip › Figure 1 - Figure Supplement 1 - Source Data 1/Fig1Sup1 - EGFR - PY.tif]

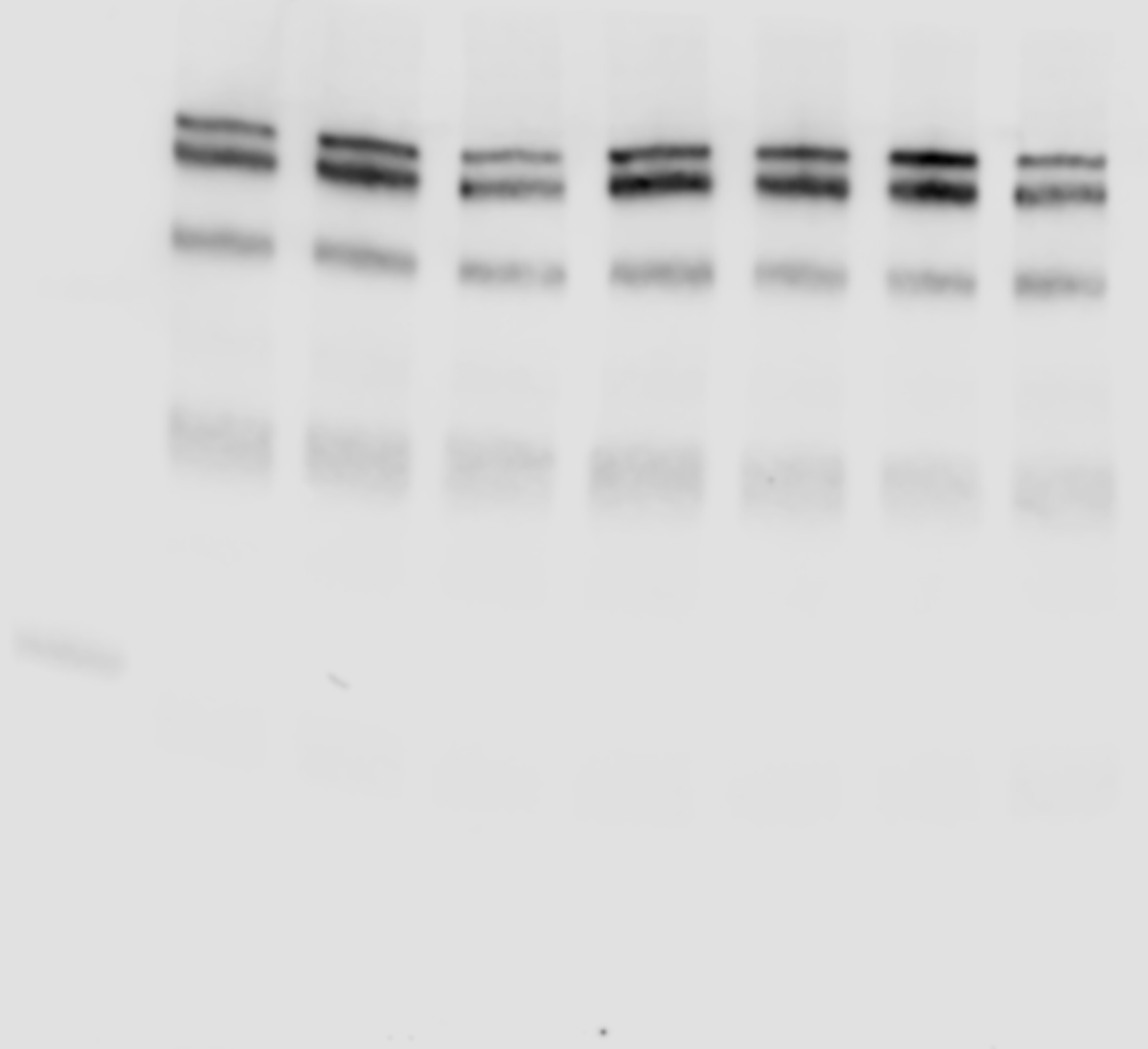

Supplement: Figure 1—figure supplement 1—source data 1. [file elife-63678-fig1-figsupp1-data1.zip › Figure 1 - Figure Supplement 1 - Source Data 1/Fig1Sup1 - RON IP - Protein.tif]

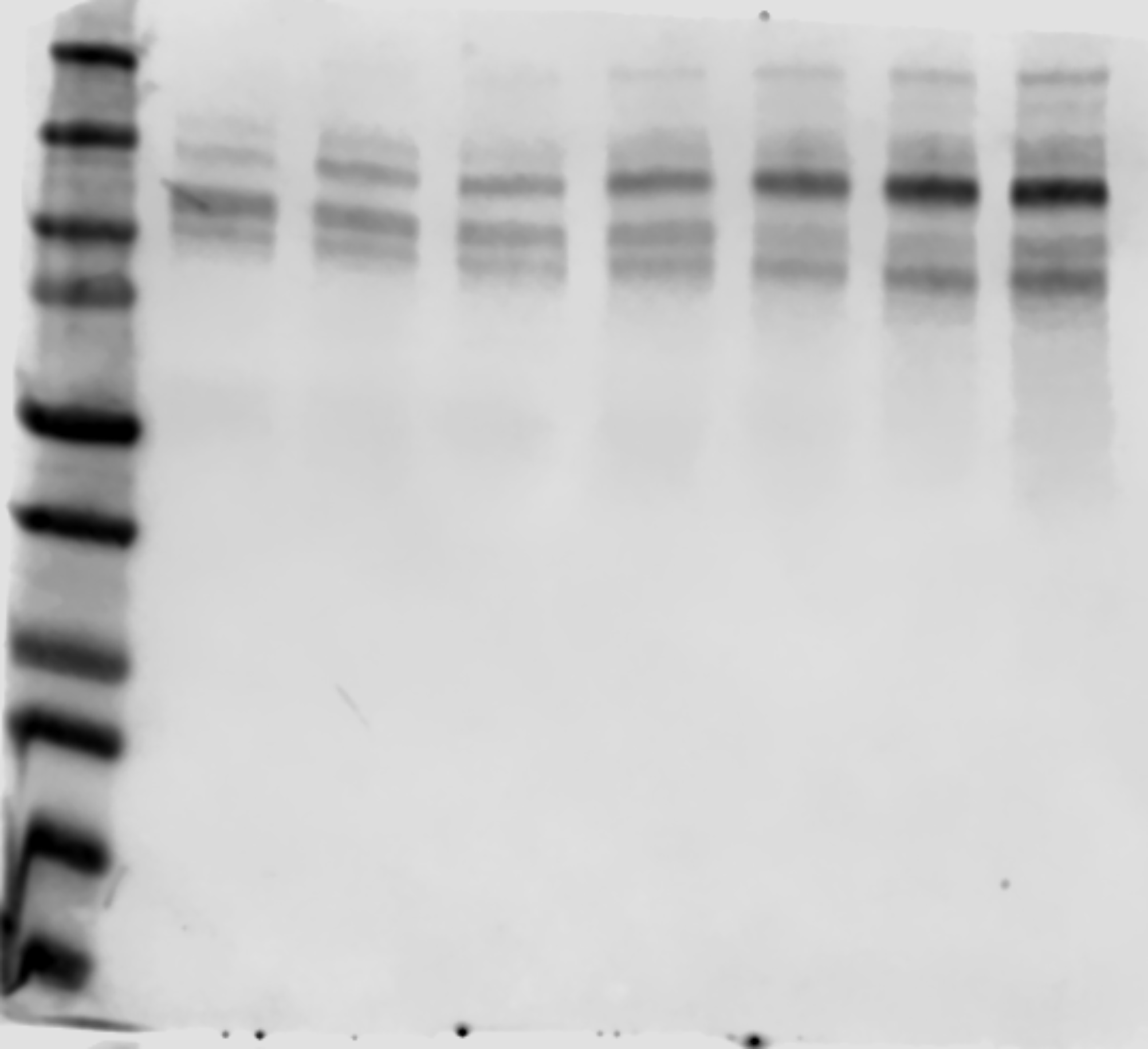

Supplement: Figure 1—figure supplement 1—source data 1. [file elife-63678-fig1-figsupp1-data1.zip › Figure 1 - Figure Supplement 1 - Source Data 1/Fig1Sup1 - RON IP - PY.tif]

Figure 1 - Figure Supplement 1 - Source Data 1

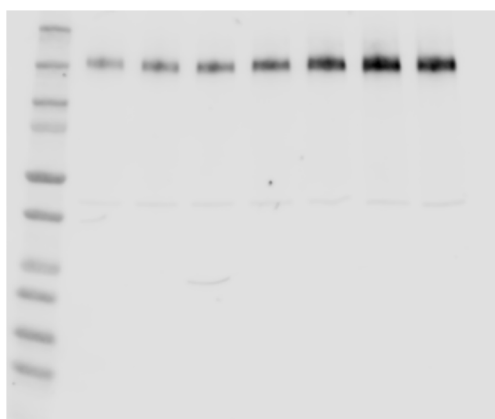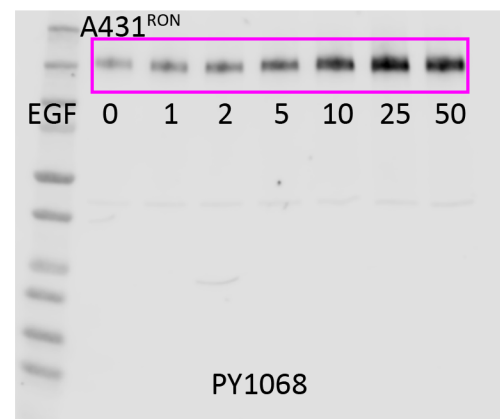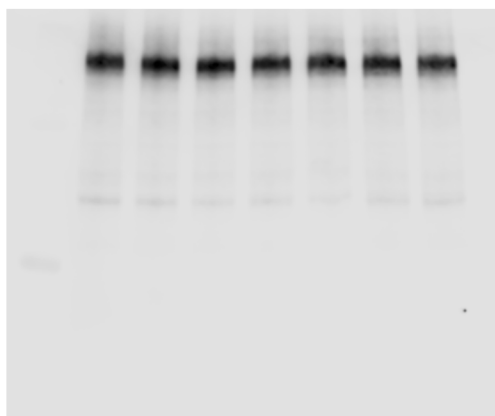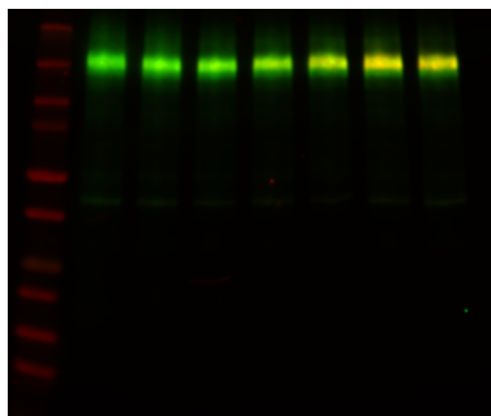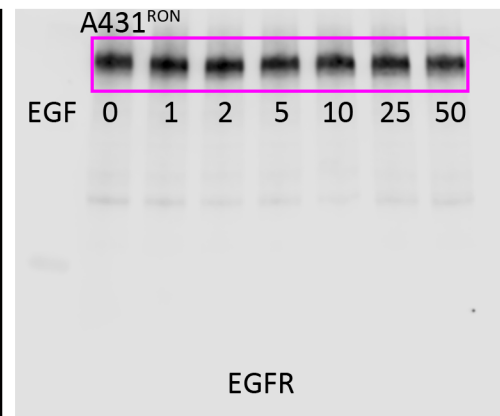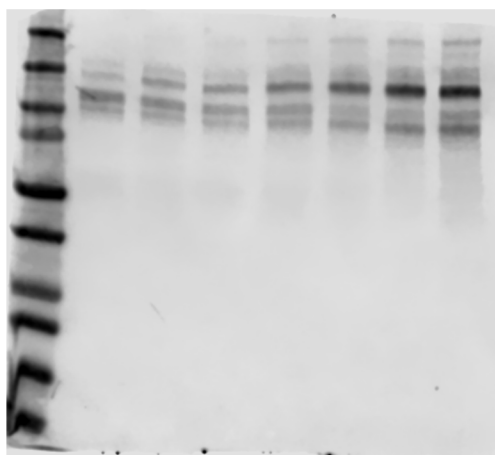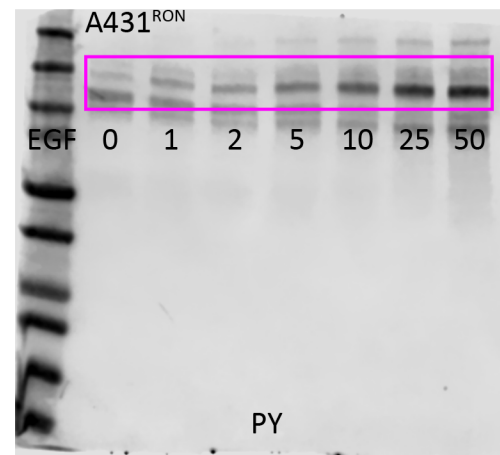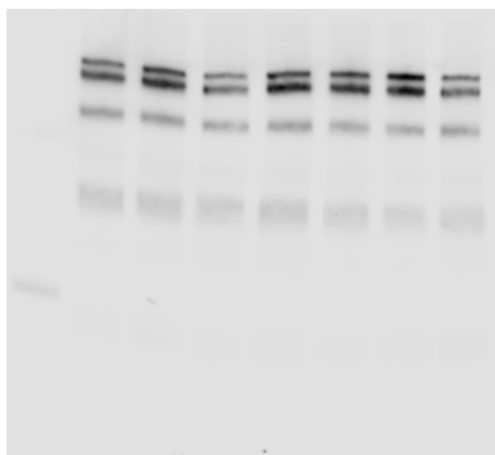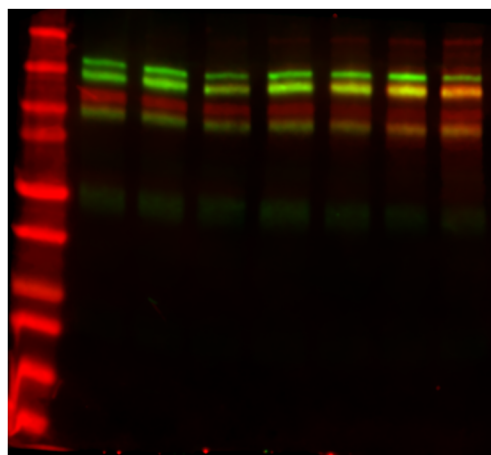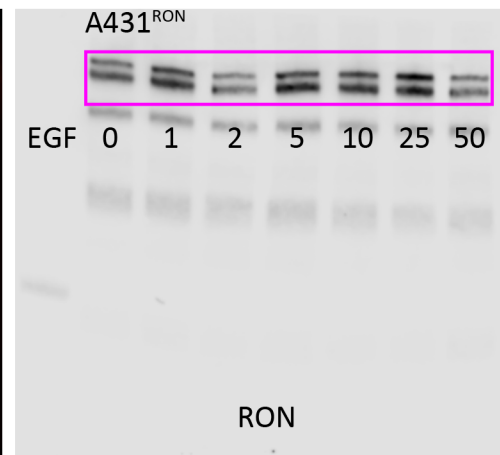

Supplement: Figure 1—figure supplement 1—source data 1. [file elife-63678-fig1-figsupp1-data1.zip › Figure 1 - Figure Supplement 1 - Source Data 1/Figure 1 - Figure Supplement 1 - Source Data 1 annotated.pdf]

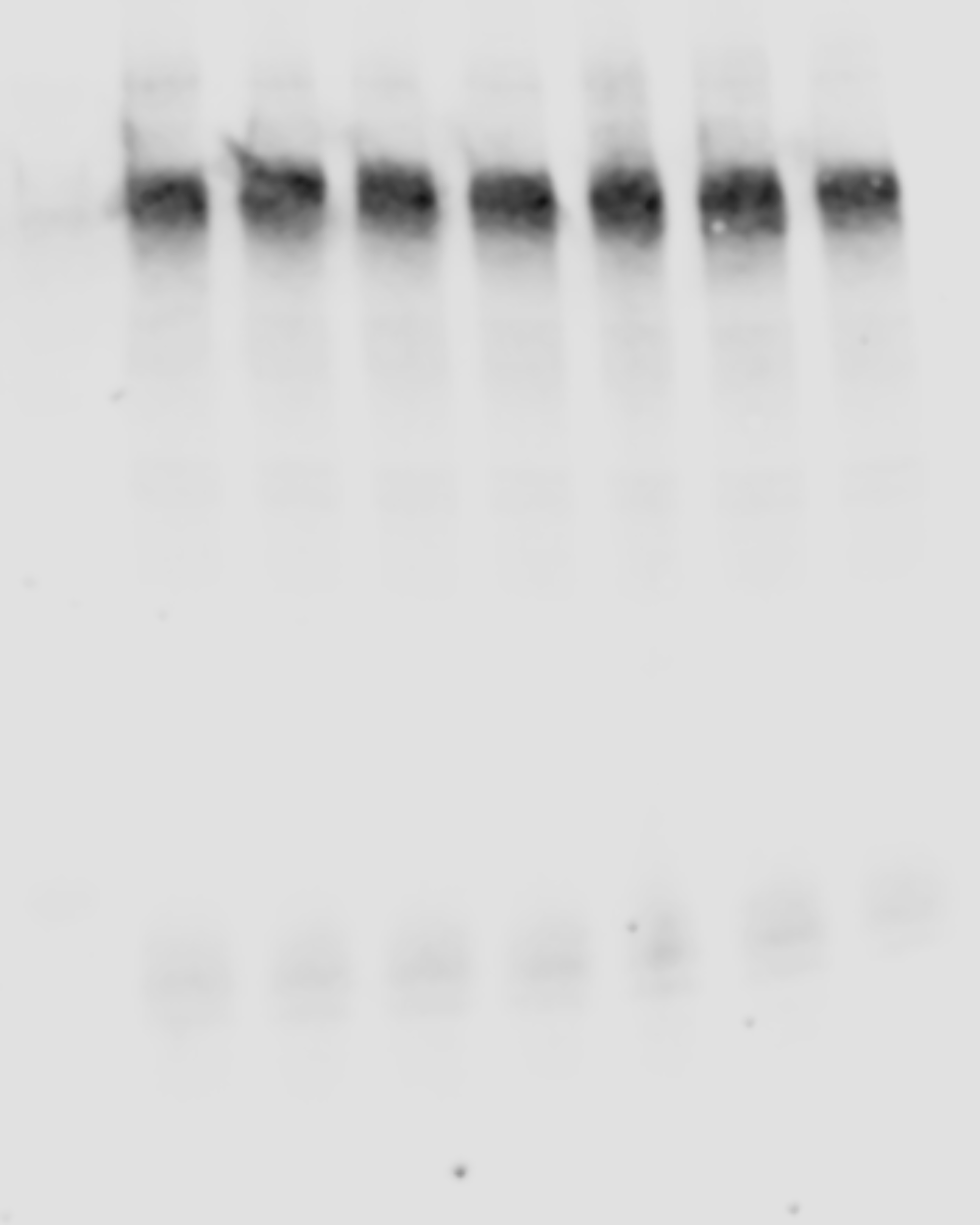

Supplement: Figure 1—figure supplement 2—source data 1. [file elife-63678-fig1-figsupp2-data1.zip › Figure 1 - Figure Supplement 2 - Source Data 1/Fig1Sup2 - EGFR - Protein.tif]

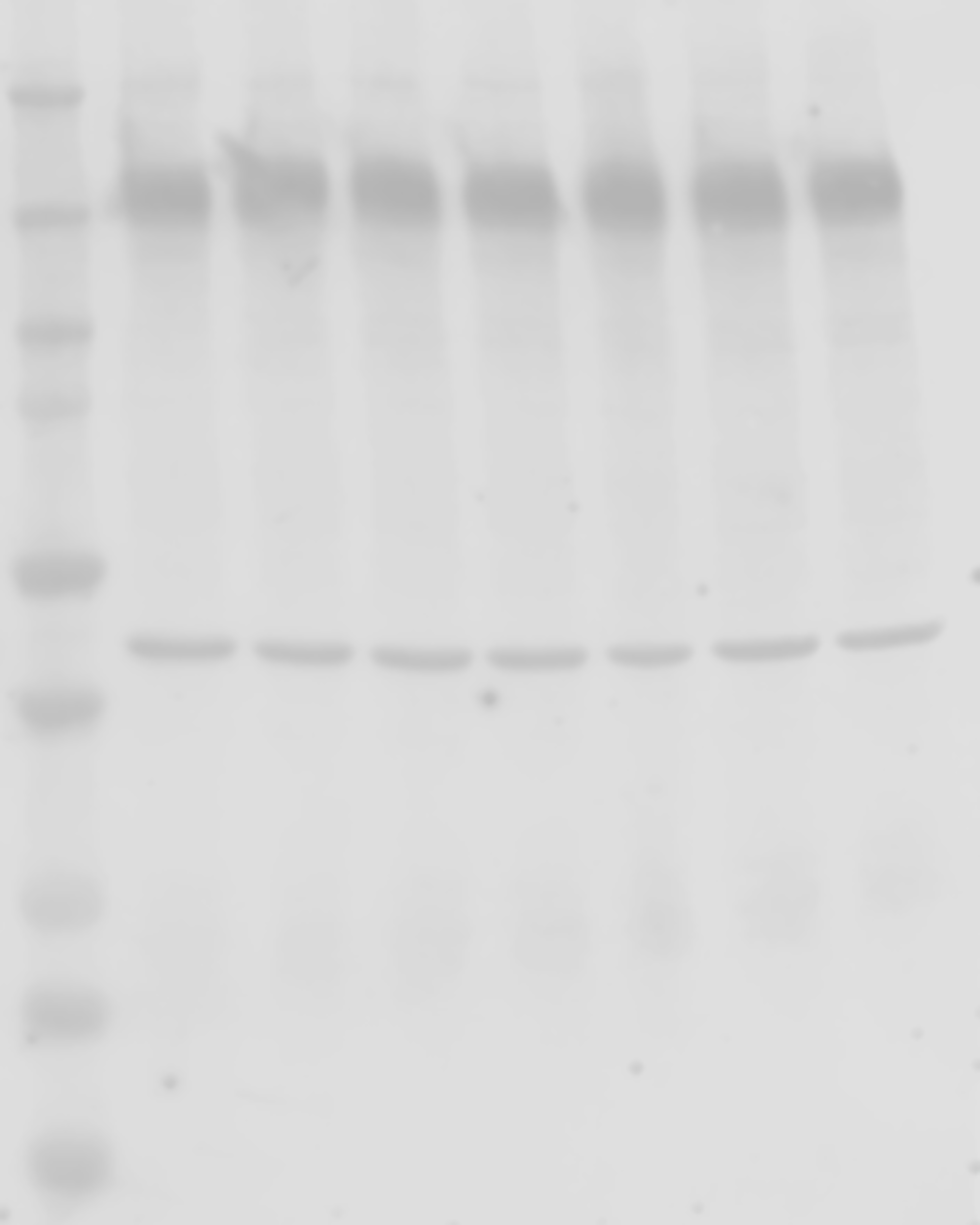

Supplement: Figure 1—figure supplement 2—source data 1. [file elife-63678-fig1-figsupp2-data1.zip › Figure 1 - Figure Supplement 2 - Source Data 1/Fig1Sup2 - EGFR - PY.tif]

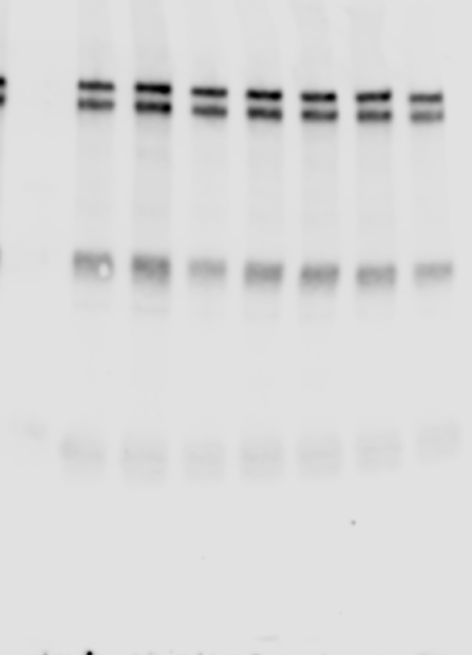

Supplement: Figure 1—figure supplement 2—source data 1. [file elife-63678-fig1-figsupp2-data1.zip › Figure 1 - Figure Supplement 2 - Source Data 1/Fig1Sup2 - RON IP - Protein.tif]

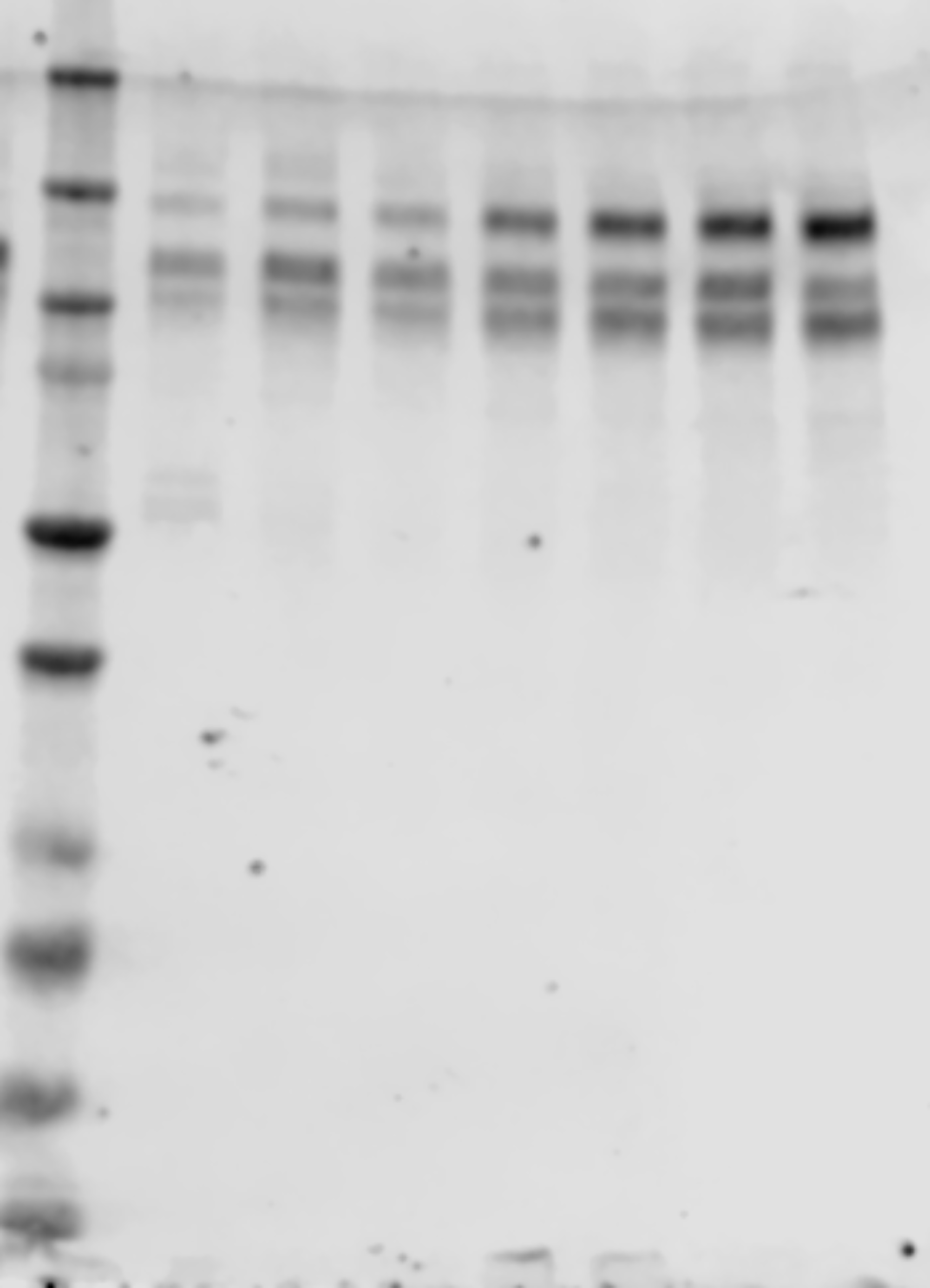

Supplement: Figure 1—figure supplement 2—source data 1. [file elife-63678-fig1-figsupp2-data1.zip › Figure 1 - Figure Supplement 2 - Source Data 1/Fig1Sup2 - RON IP - PY.tif]

Figure 1 - Figure Supplement 2 - Source Data 1

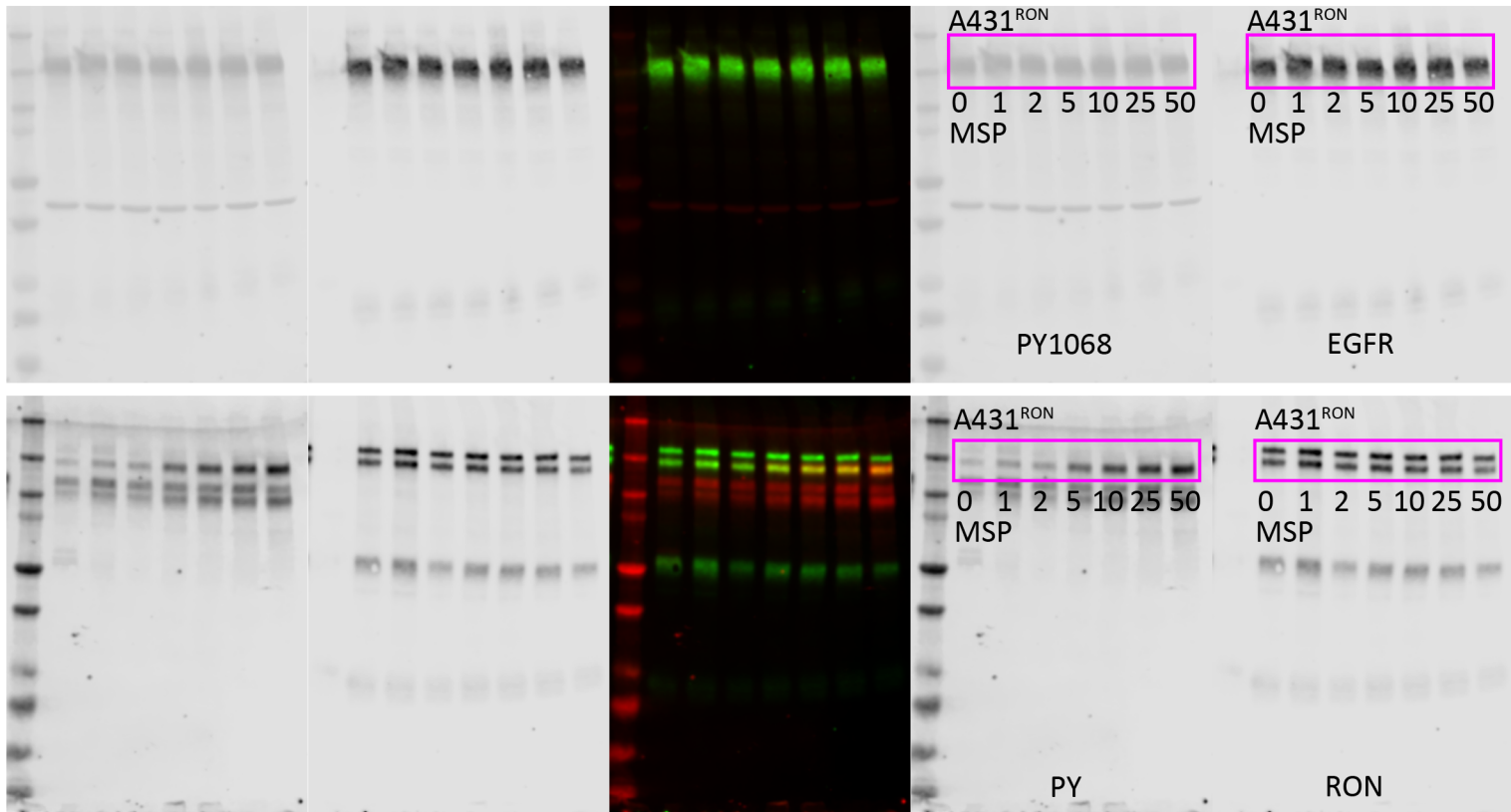

Supplement: Figure 1—figure supplement 2—source data 1. [file elife-63678-fig1-figsupp2-data1.zip › Figure 1 - Figure Supplement 2 - Source Data 1/Figure 1 - Figure Supplement 2 - Source Data 1 - Annotated.pdf]

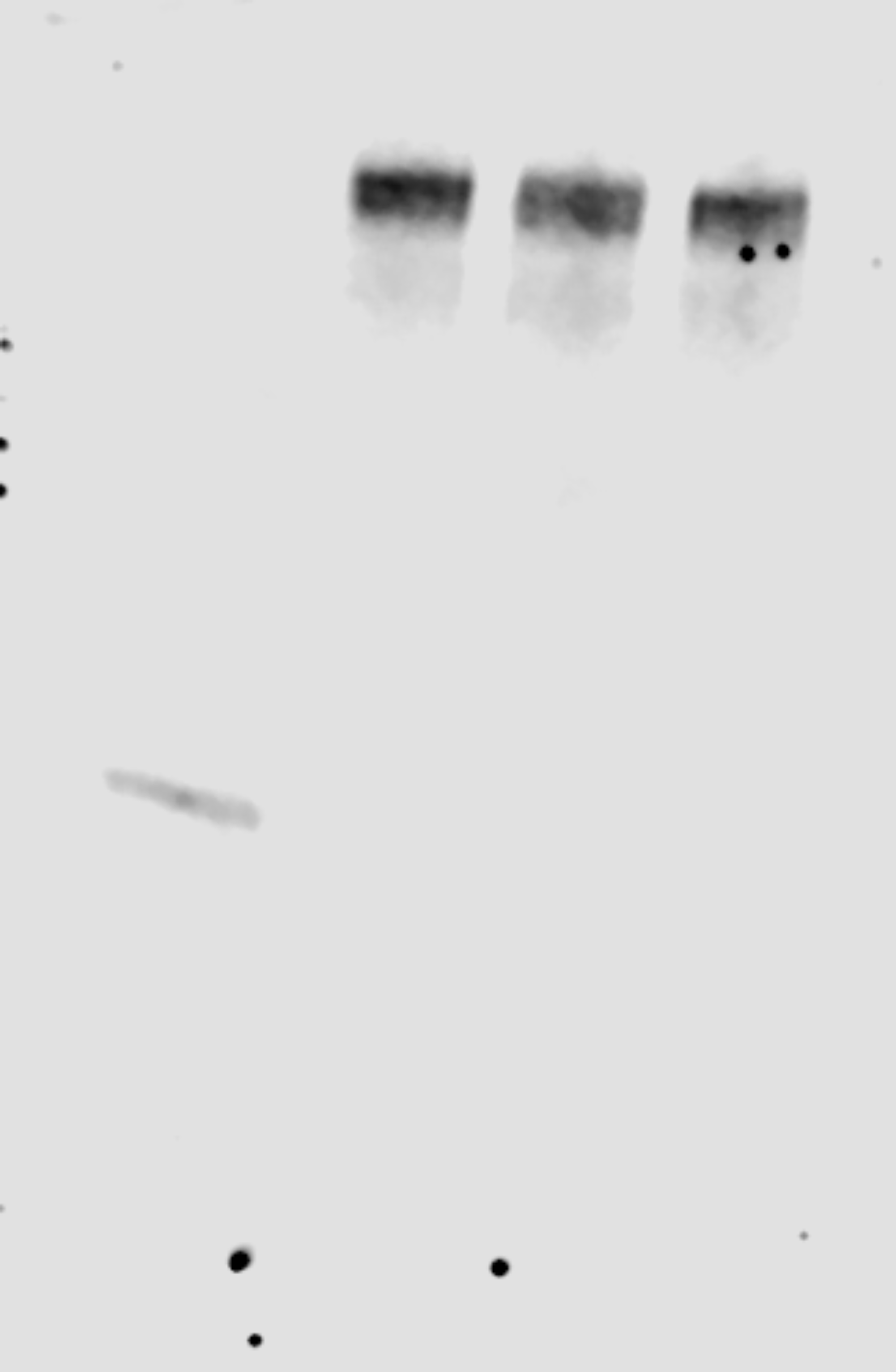

Supplement: Figure 1—figure supplement 3—source data 1. [file elife-63678-fig1-figsupp3-data1.zip › Figure 1 - Figure Supplement 3 - Source Data 1/Fig1Sup3 - EGFRPY - Protein.tif]

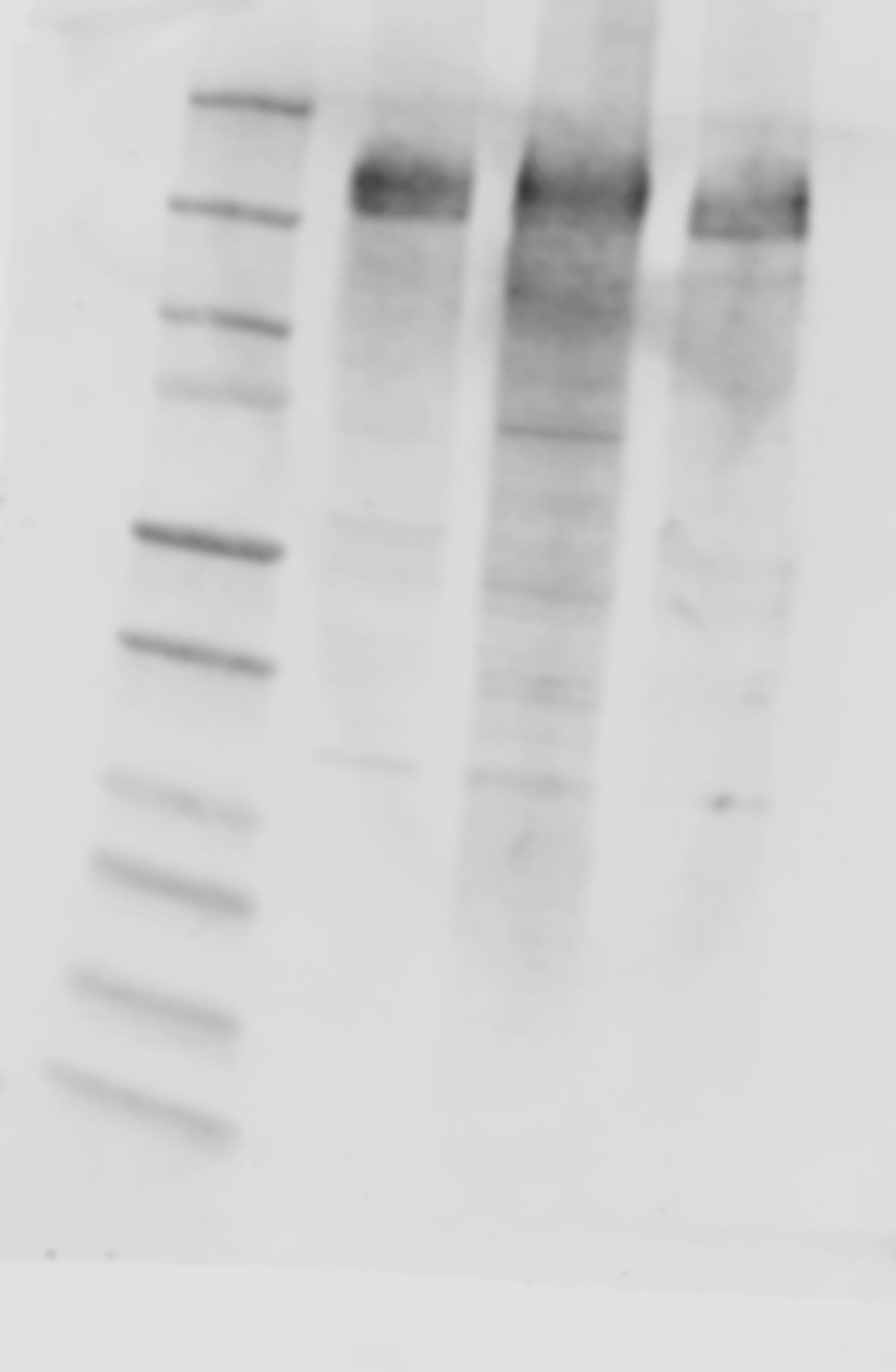

Supplement: Figure 1—figure supplement 3—source data 1. [file elife-63678-fig1-figsupp3-data1.zip › Figure 1 - Figure Supplement 3 - Source Data 1/Fig1Sup3 - EGFRPY - PY.tif]

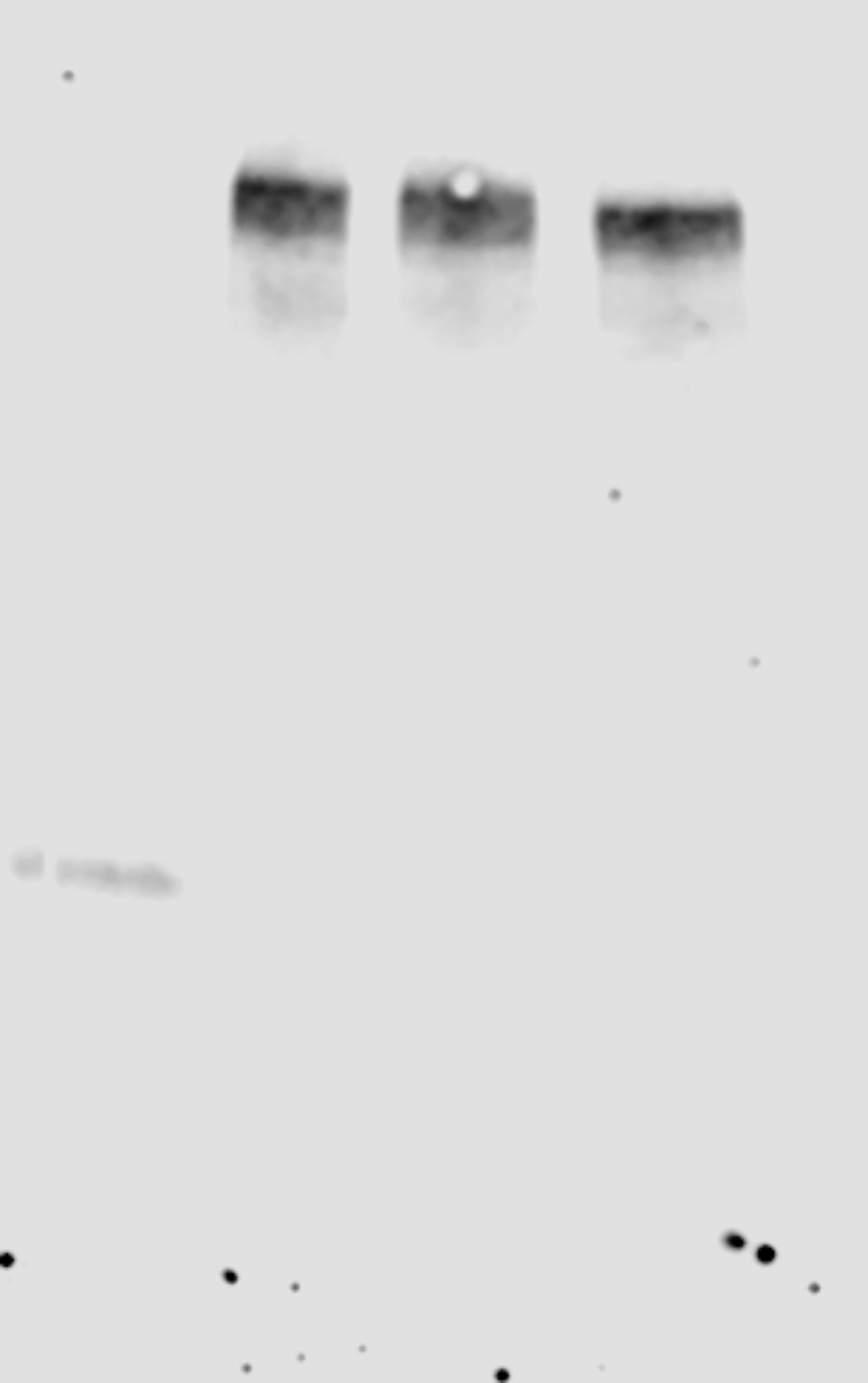

Supplement: Figure 1—figure supplement 3—source data 1. [file elife-63678-fig1-figsupp3-data1.zip › Figure 1 - Figure Supplement 3 - Source Data 1/Fig1Sup3 - EGFRPY1068 - Protein.tif]

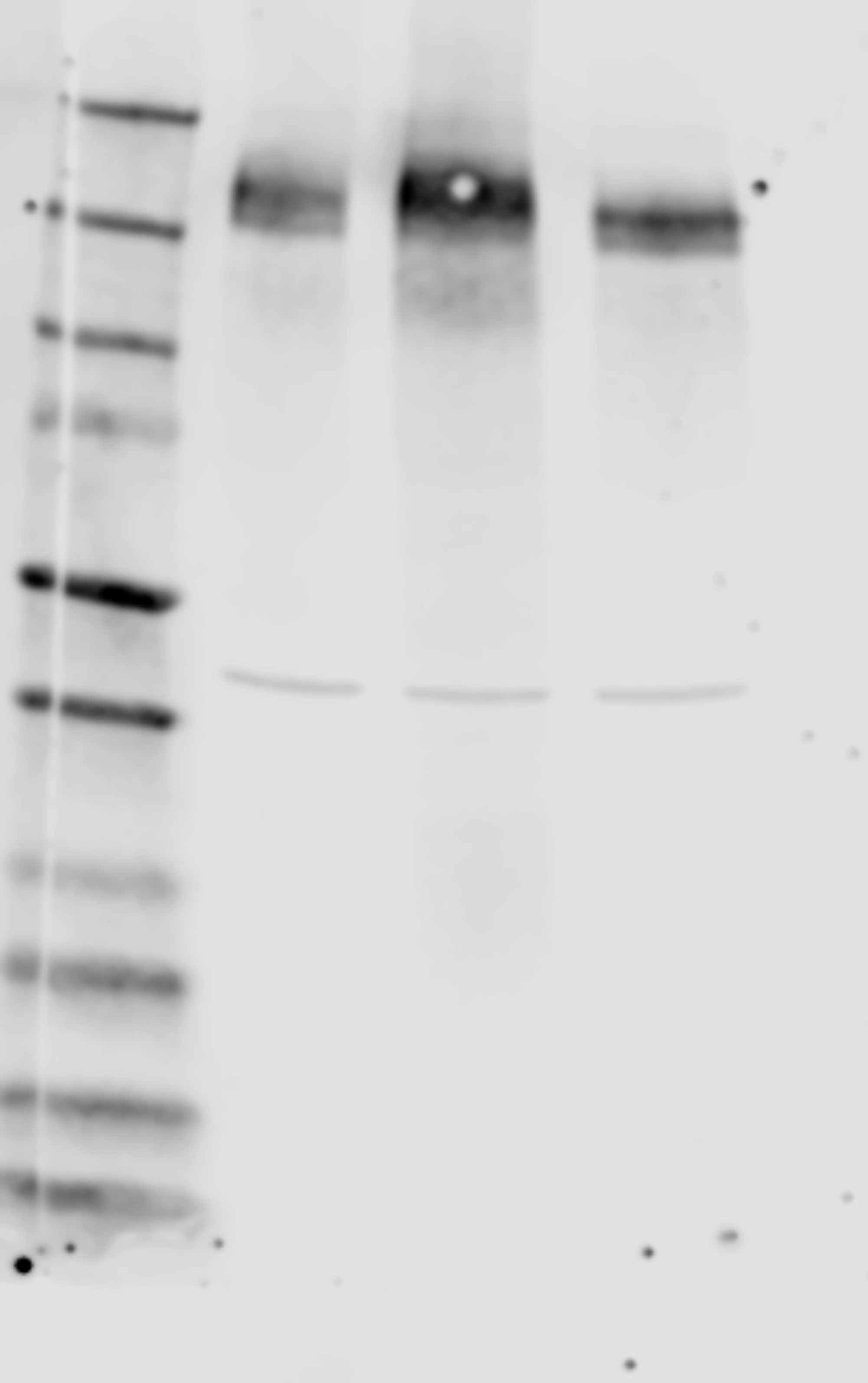

Supplement: Figure 1—figure supplement 3—source data 1. [file elife-63678-fig1-figsupp3-data1.zip › Figure 1 - Figure Supplement 3 - Source Data 1/Fig1Sup3 - EGFRPY1068 - PY.tif]

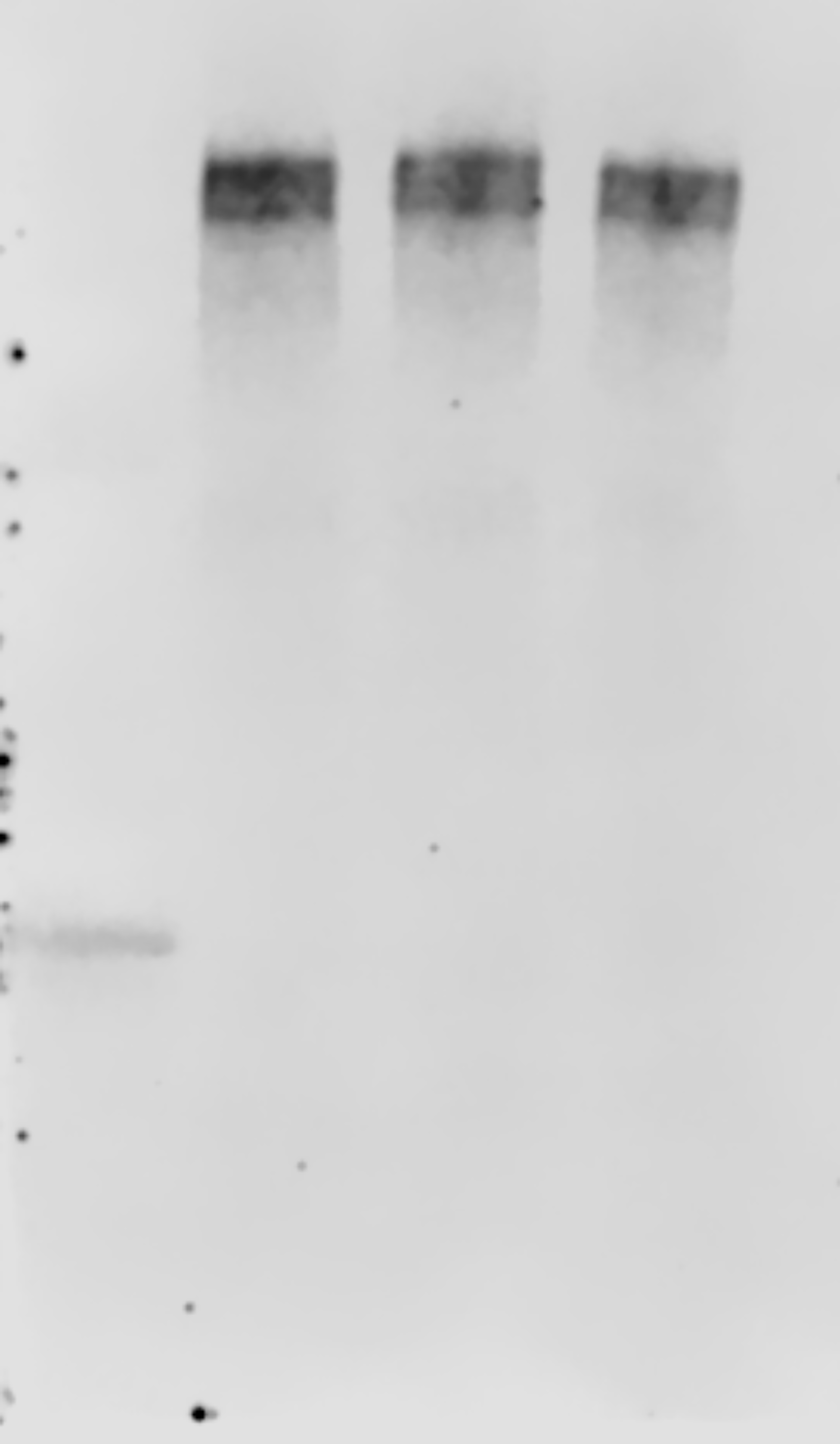

Supplement: Figure 1—figure supplement 3—source data 1. [file elife-63678-fig1-figsupp3-data1.zip › Figure 1 - Figure Supplement 3 - Source Data 1/Fig1Sup3 - EGFRPY1148 - Protein.tif]

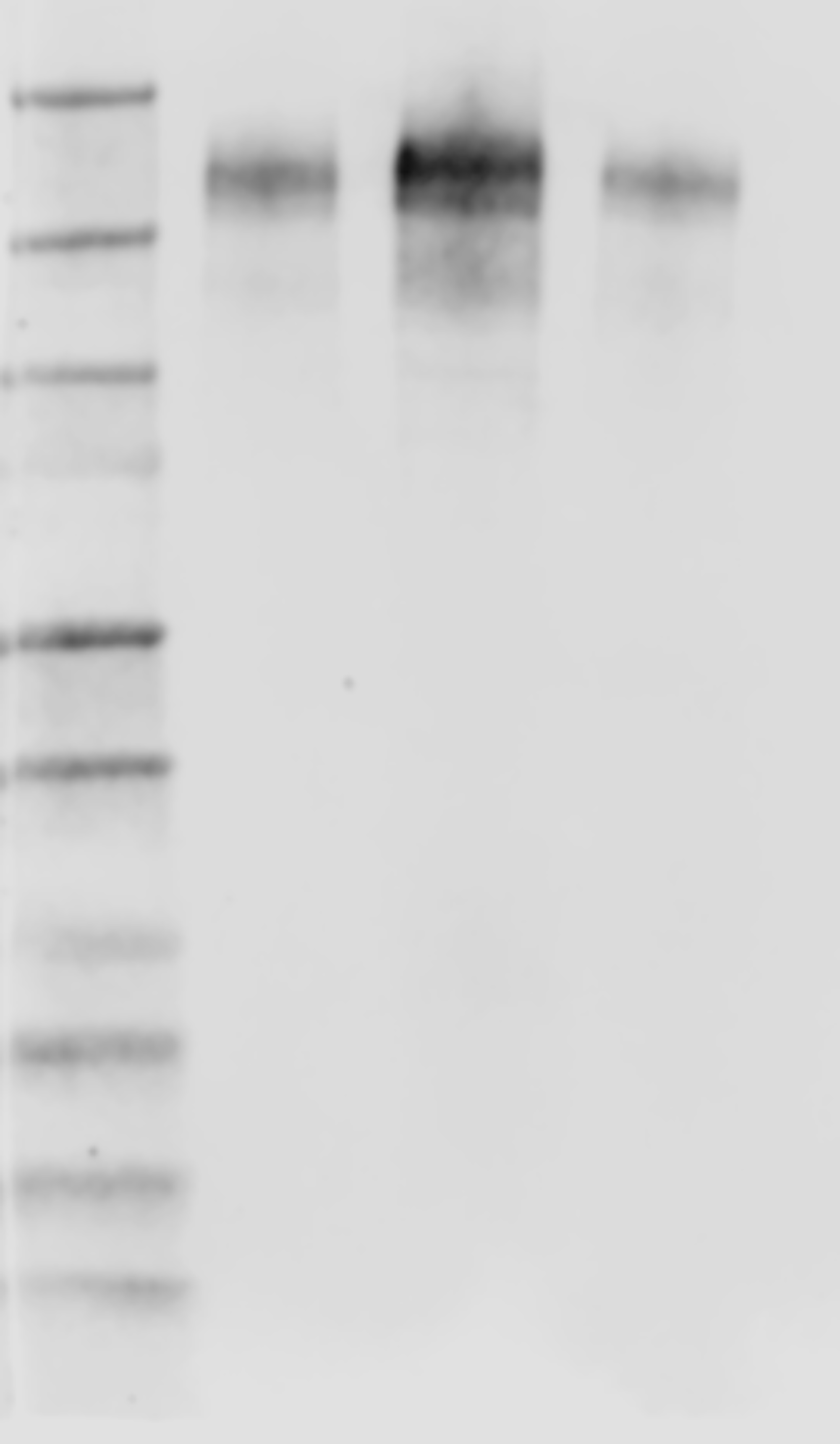

Supplement: Figure 1—figure supplement 3—source data 1. [file elife-63678-fig1-figsupp3-data1.zip › Figure 1 - Figure Supplement 3 - Source Data 1/Fig1Sup3 - EGFRPY1148 - PY.tif]

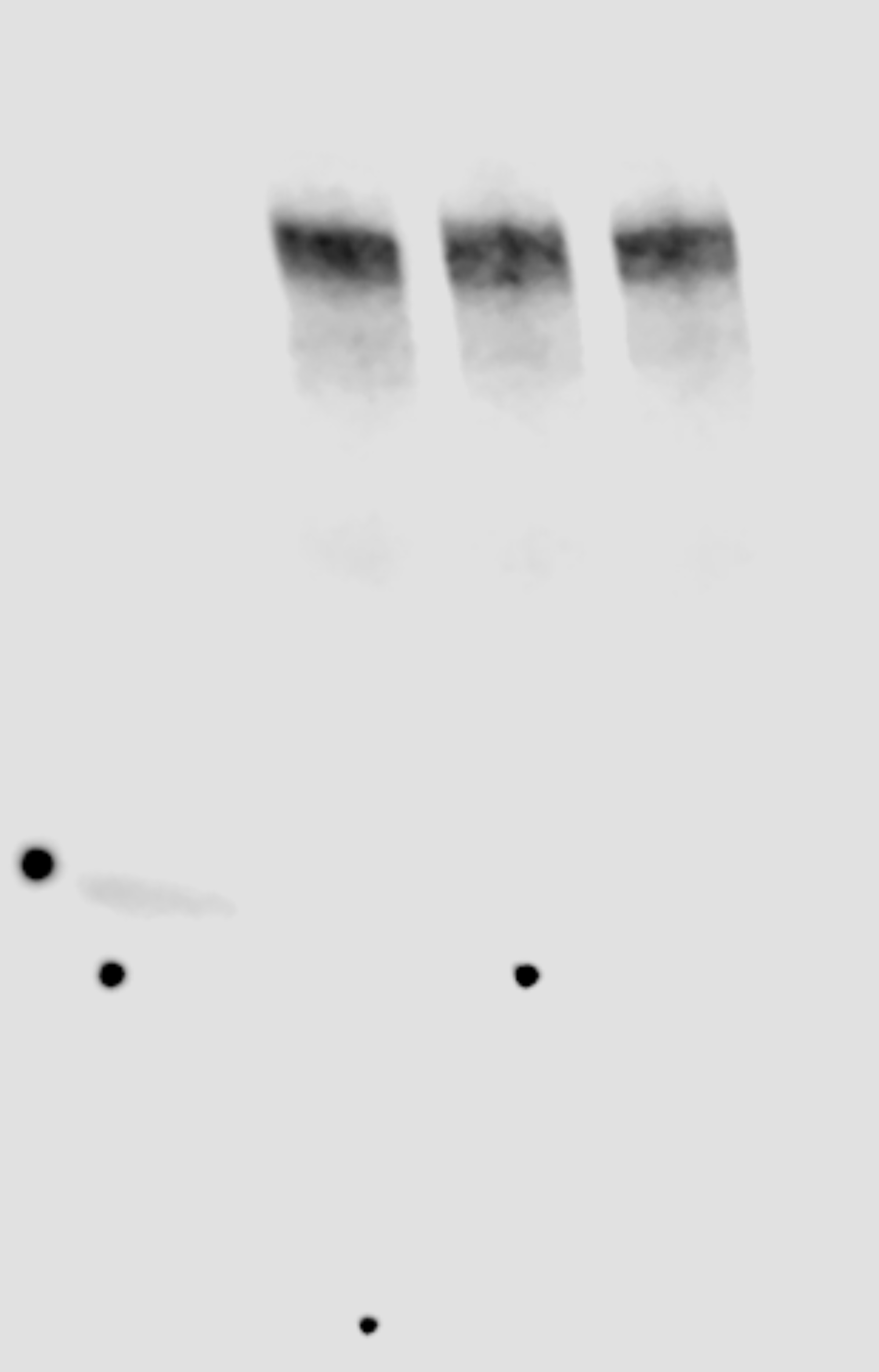

Supplement: Figure 1—figure supplement 3—source data 1. [file elife-63678-fig1-figsupp3-data1.zip › Figure 1 - Figure Supplement 3 - Source Data 1/Fig1Sup3 - EGFRPY845 - Protein.tif]

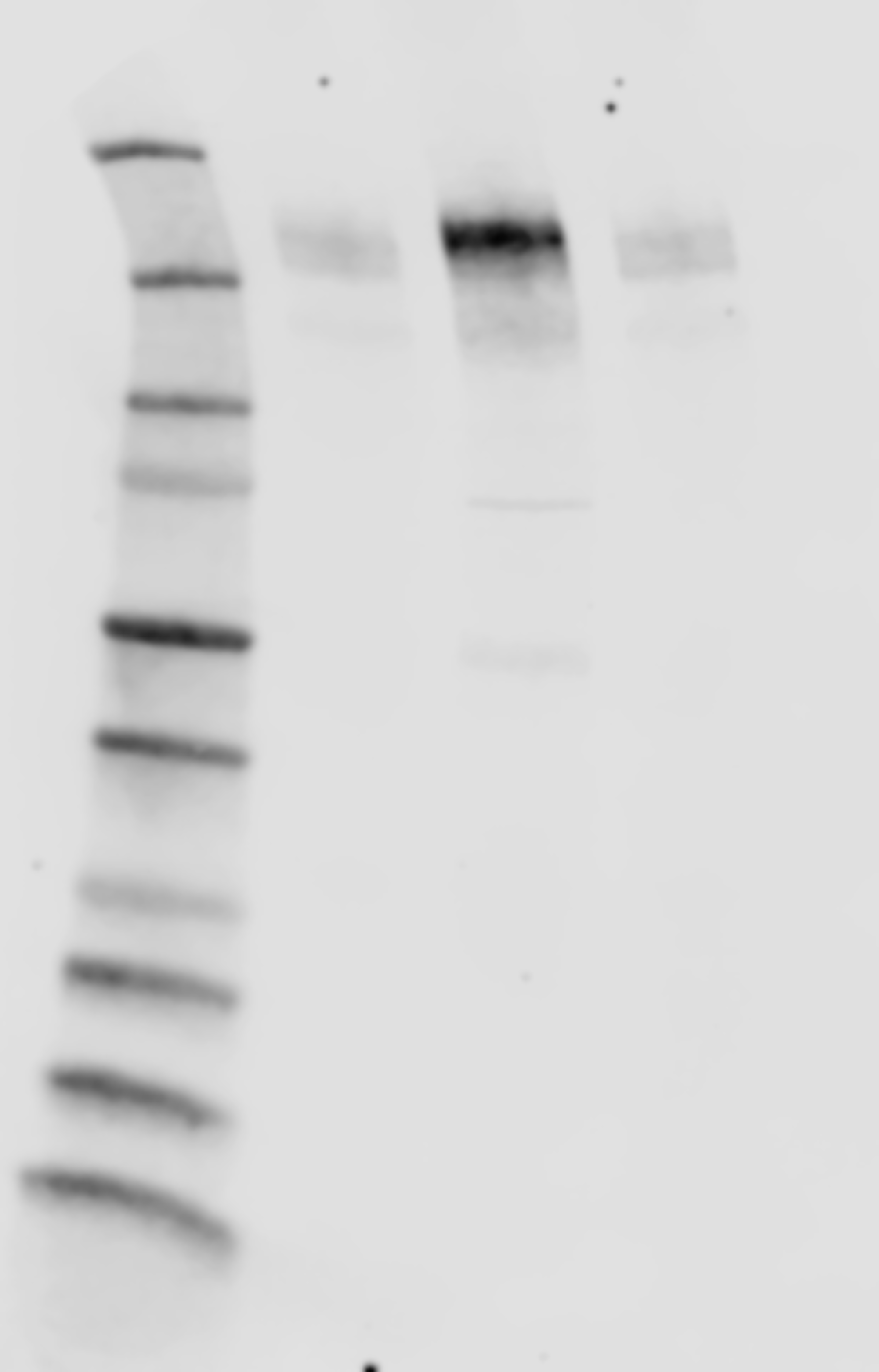

Supplement: Figure 1—figure supplement 3—source data 1. [file elife-63678-fig1-figsupp3-data1.zip › Figure 1 - Figure Supplement 3 - Source Data 1/Fig1Sup3 - EGFRPY845 - PY.tif]

Figure 1 - Figure Supplement 3 - Source Data 1

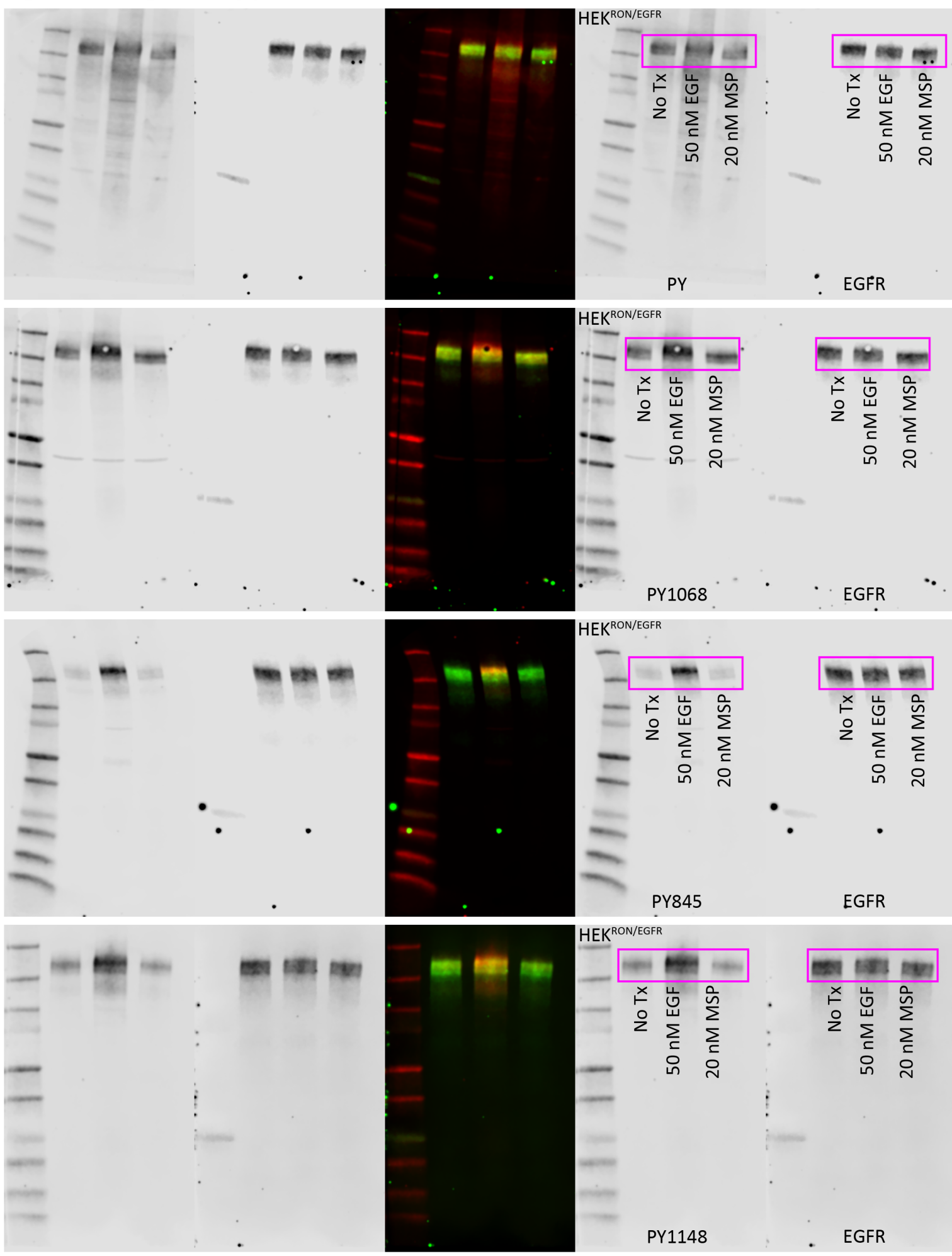

Supplement: Figure 1—figure supplement 3—source data 1. [file elife-63678-fig1-figsupp3-data1.zip › Figure 1 - Figure Supplement 3 - Source Data 1/Figure 1 - Figure Supplement 3 - Source Data 1 - Annotated.pdf]

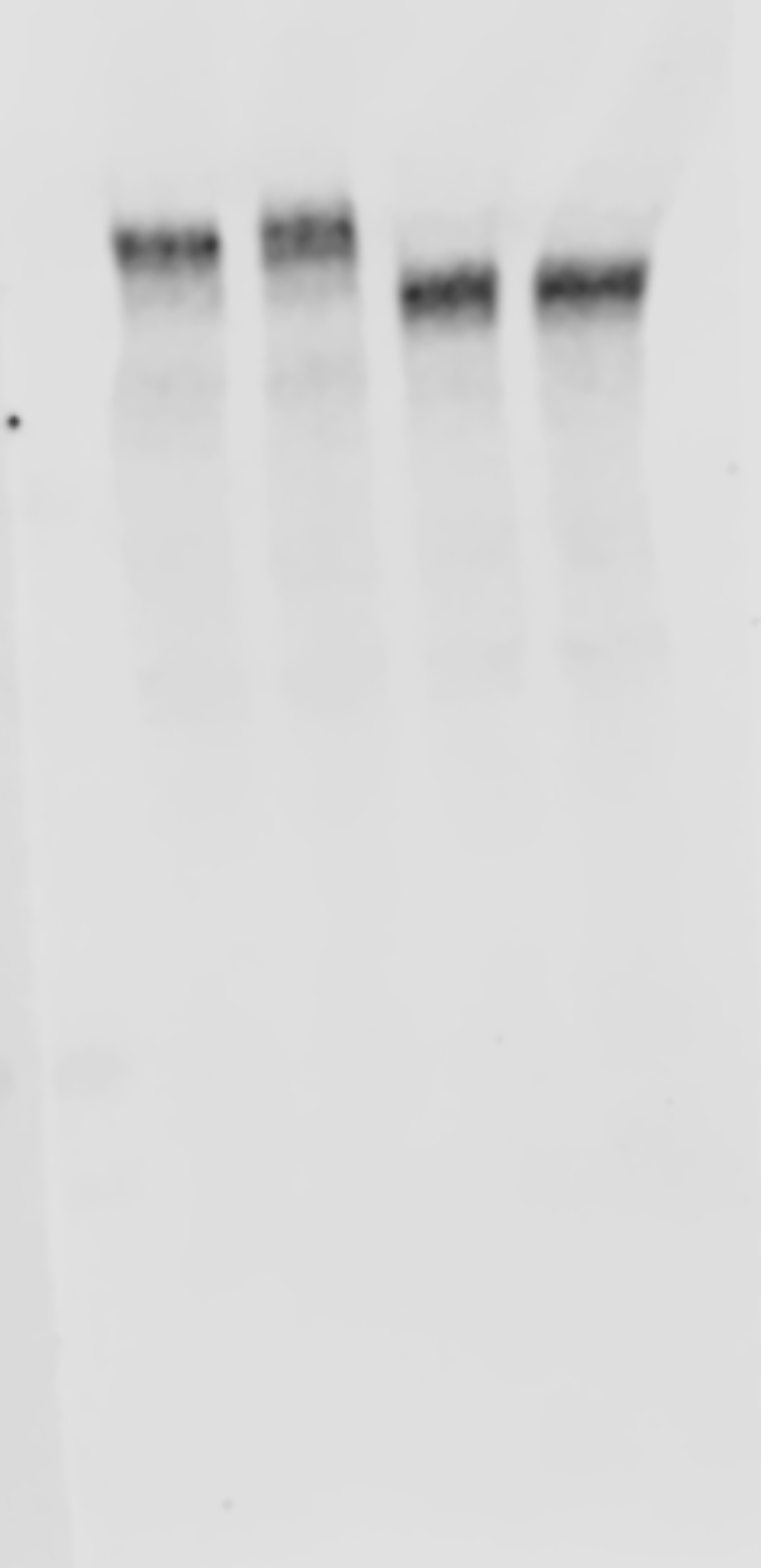

Supplement: Figure 1—figure supplement 4—source data 1. [file elife-63678-fig1-figsupp4-data1.zip › Figure 1 - Figure Supplement 4 - Source Data 1/Fig1Sup4 - RONEGFR - EGFR Protein.tif]

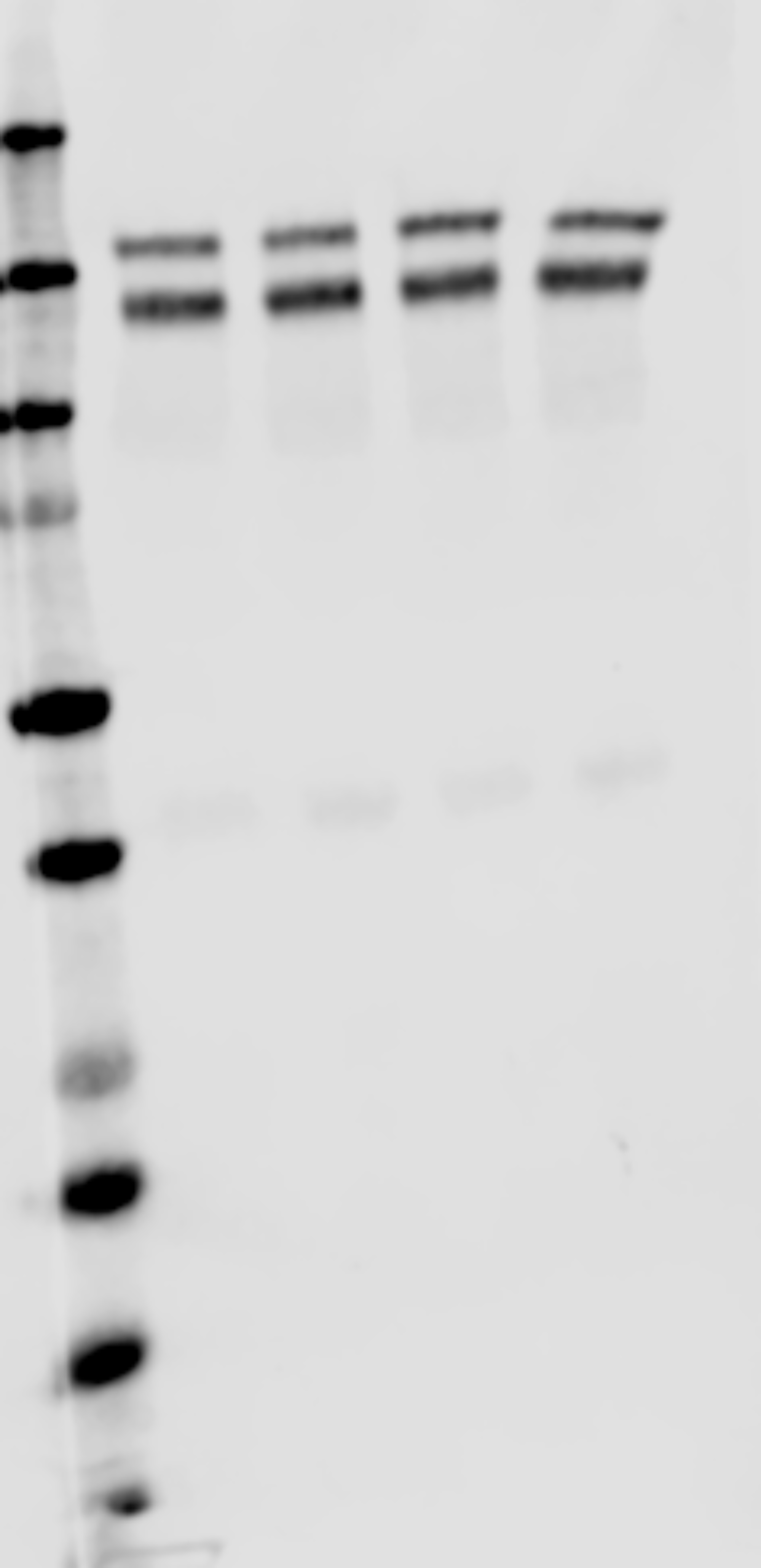

Supplement: Figure 1—figure supplement 4—source data 1. [file elife-63678-fig1-figsupp4-data1.zip › Figure 1 - Figure Supplement 4 - Source Data 1/Fig1Sup4 - RONEGFR - RON Protein.tif]

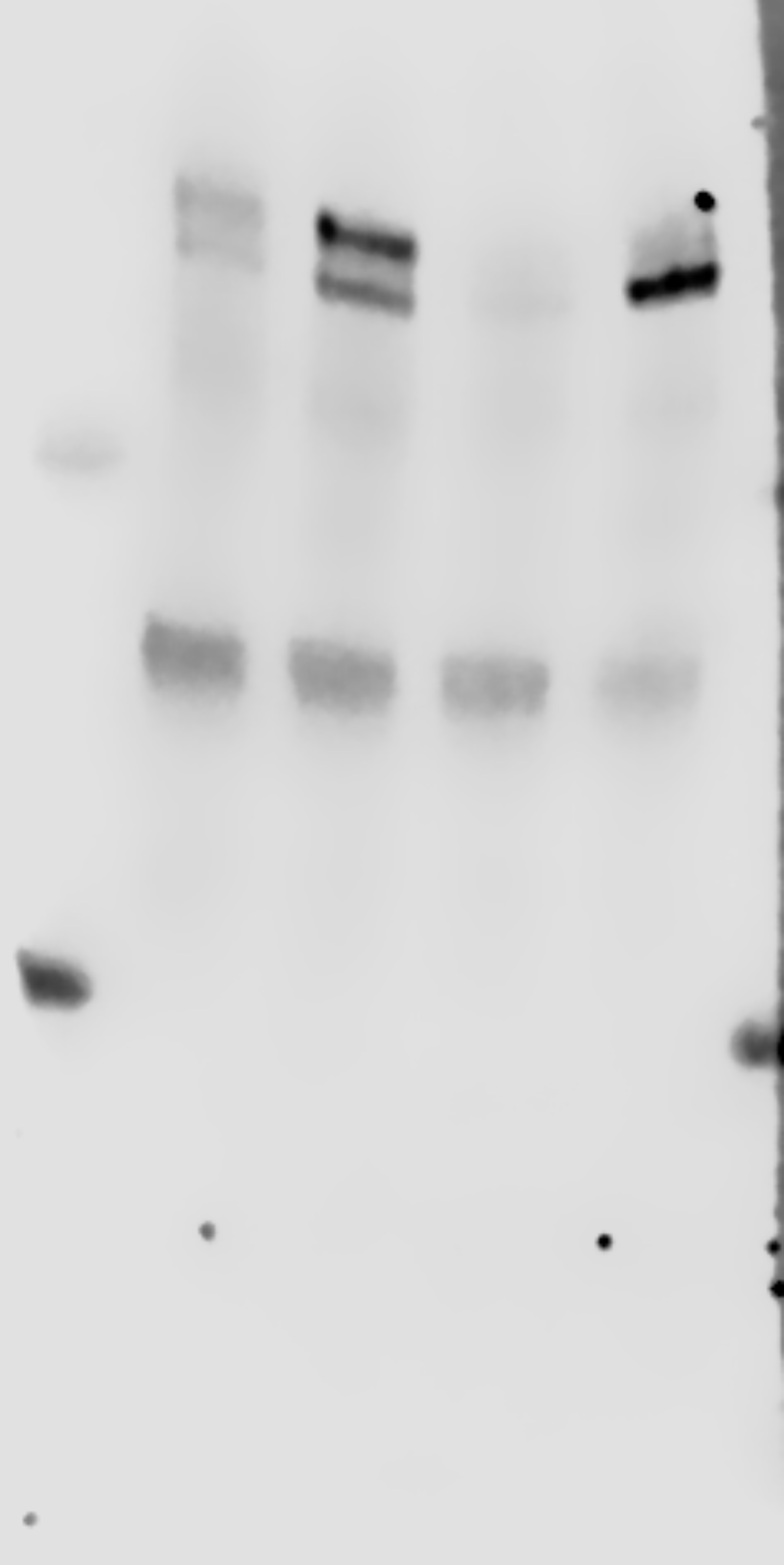

Supplement: Figure 1—figure supplement 4—source data 1. [file elife-63678-fig1-figsupp4-data1.zip › Figure 1 - Figure Supplement 4 - Source Data 1/Fig1Sup4 - RONPY1238 - Protein.tif]

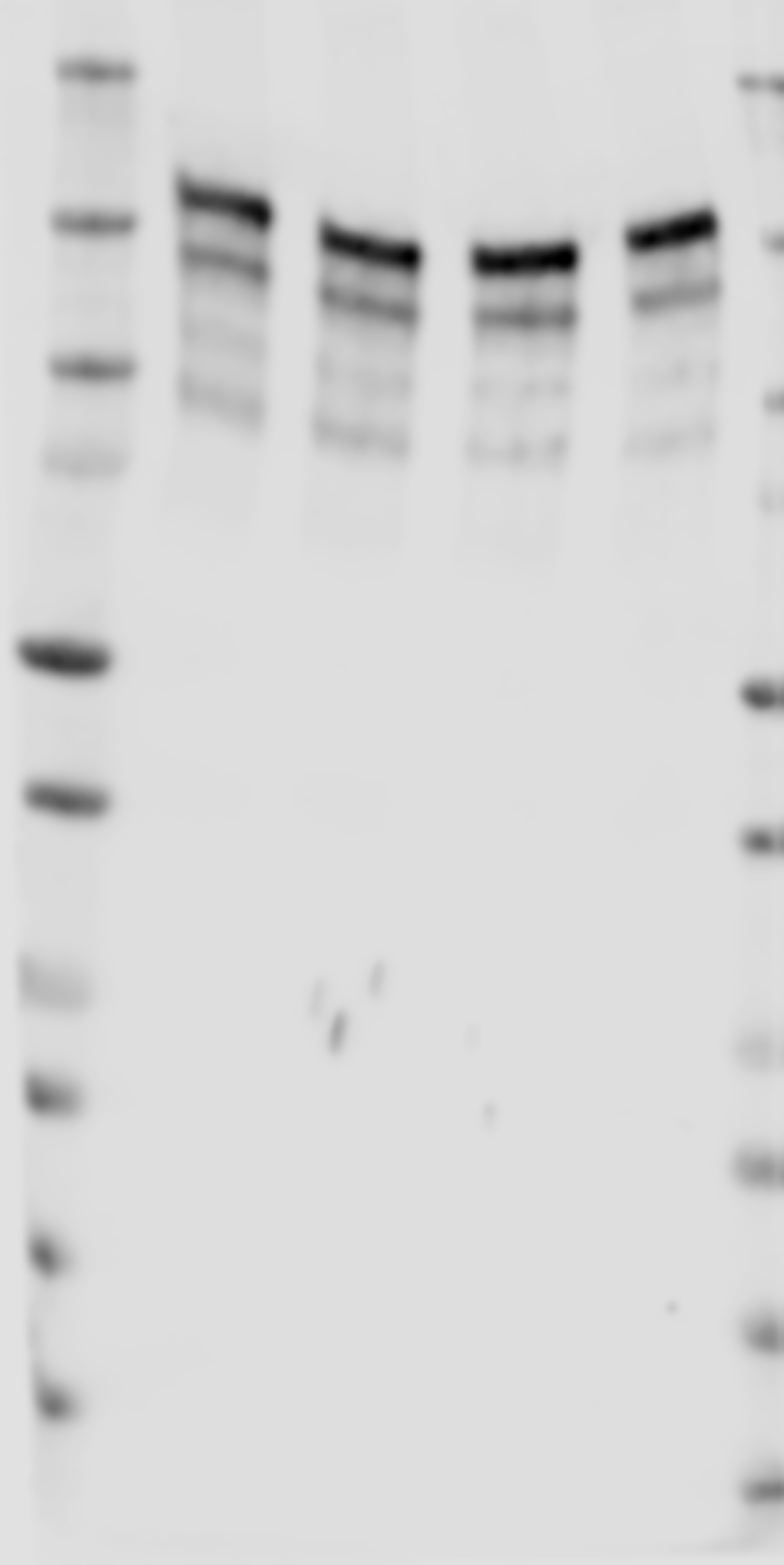

Supplement: Figure 1—figure supplement 4—source data 1. [file elife-63678-fig1-figsupp4-data1.zip › Figure 1 - Figure Supplement 4 - Source Data 1/Fig1Sup4 - RONPY1238 - PY.tif]

Figure 1 - Figure Supplement 4 - Source Data 1

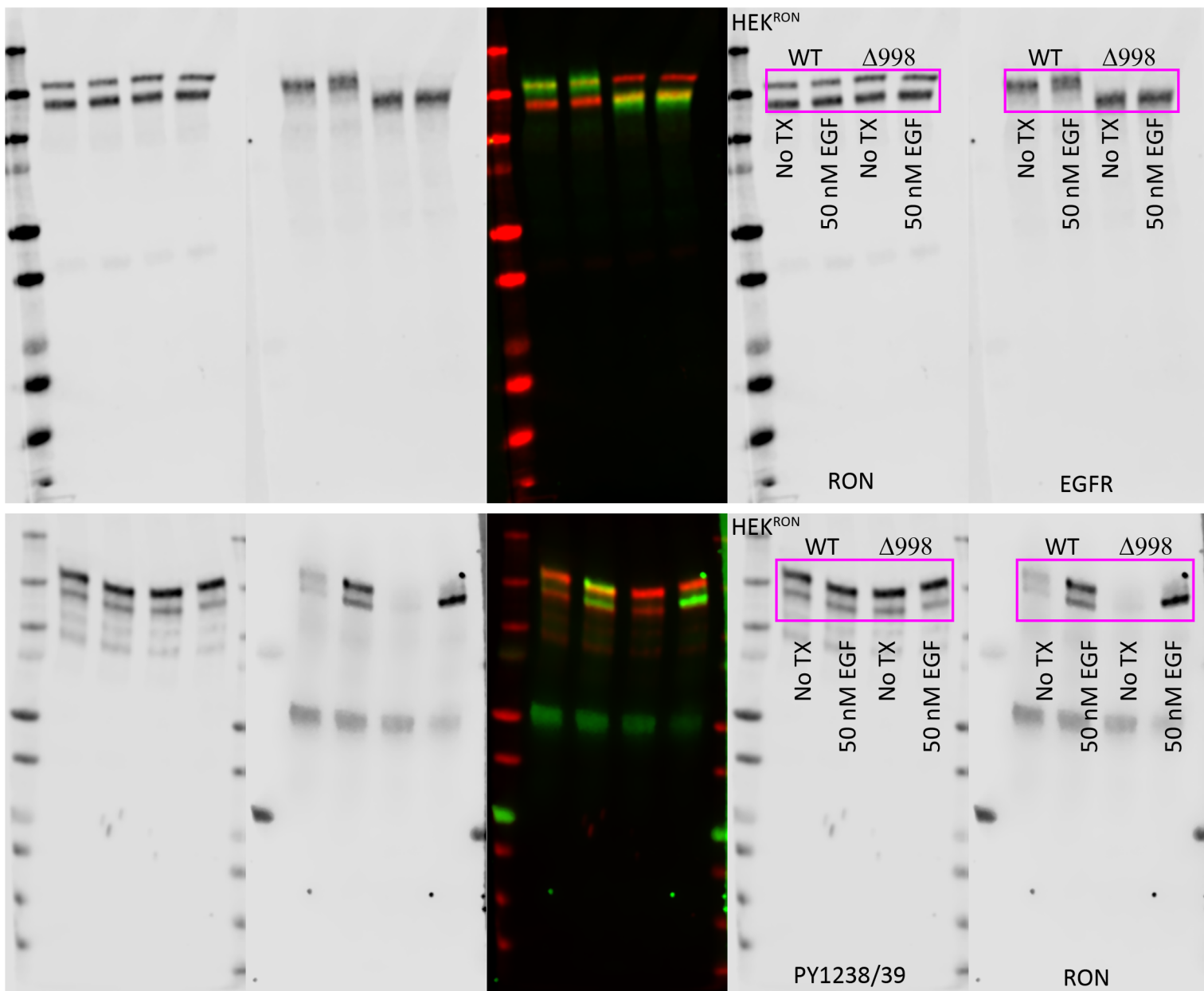

Supplement: Figure 1—figure supplement 4—source data 1. [file elife-63678-fig1-figsupp4-data1.zip › Figure 1 - Figure Supplement 4 - Source Data 1/Figure 1 - Figure Supplement 4 - Source Data 1 - Annotated.pdf]

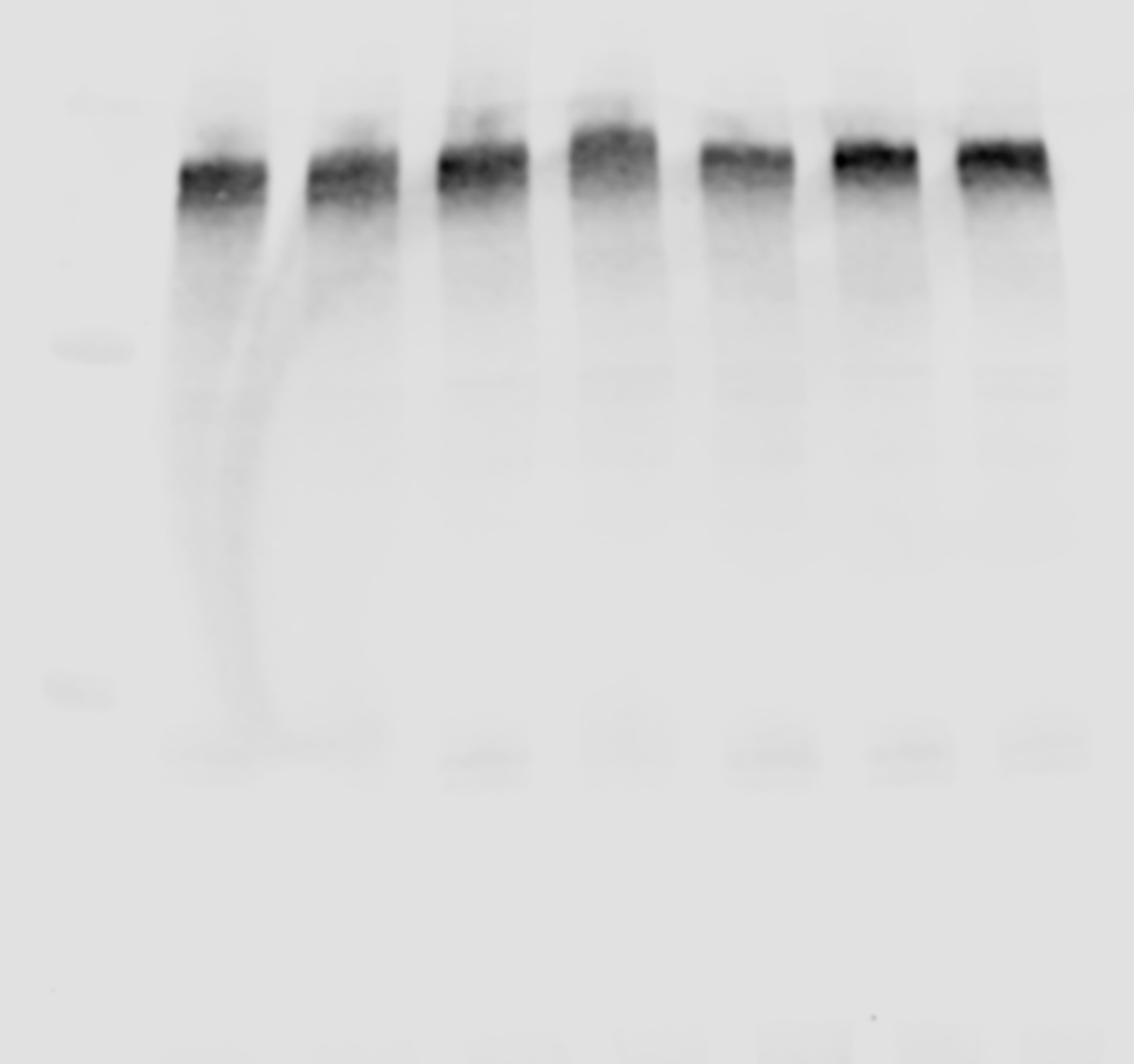

Supplement: Figure 5—source data 1. [file elife-63678-fig5-data1.zip › Figure 5 - Source Data 1/Fig5A - EGFR - Protein.tif]

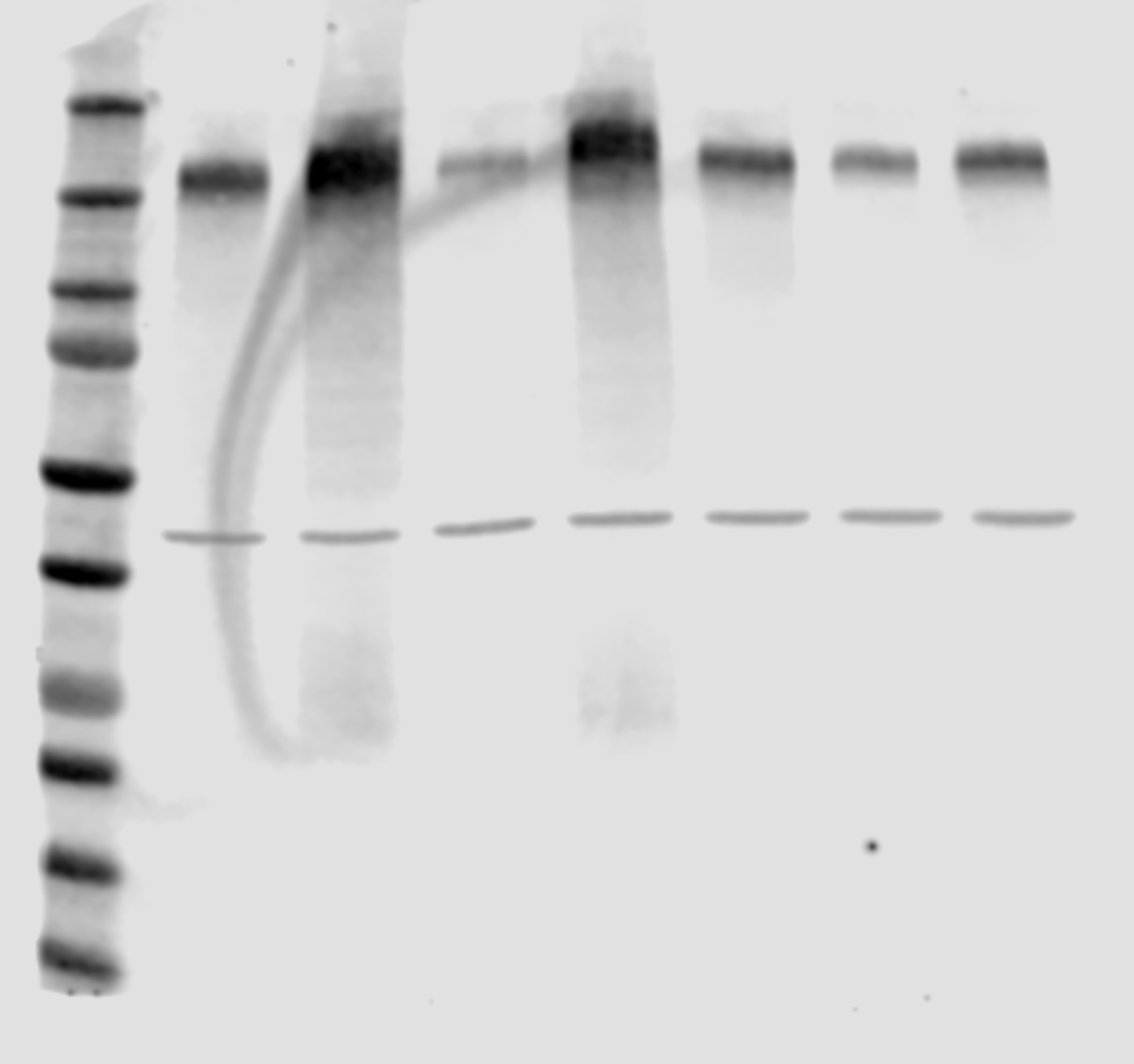

Supplement: Figure 5—source data 1. [file elife-63678-fig5-data1.zip › Figure 5 - Source Data 1/Fig5A - EGFR - PY.tif]

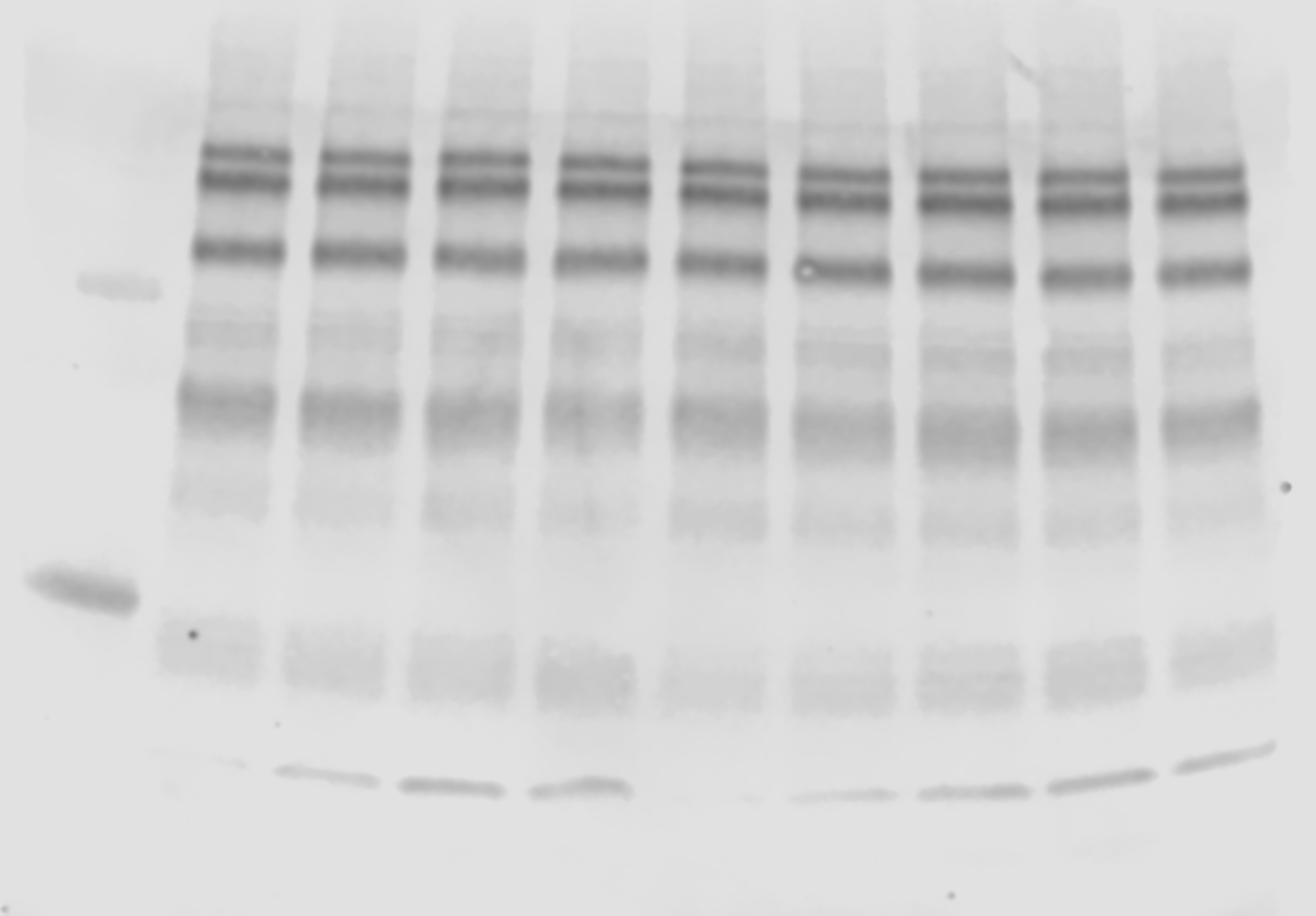

Supplement: Figure 5—source data 1. [file elife-63678-fig5-data1.zip › Figure 5 - Source Data 1/Fig5B - IP RON - Protein.tif]

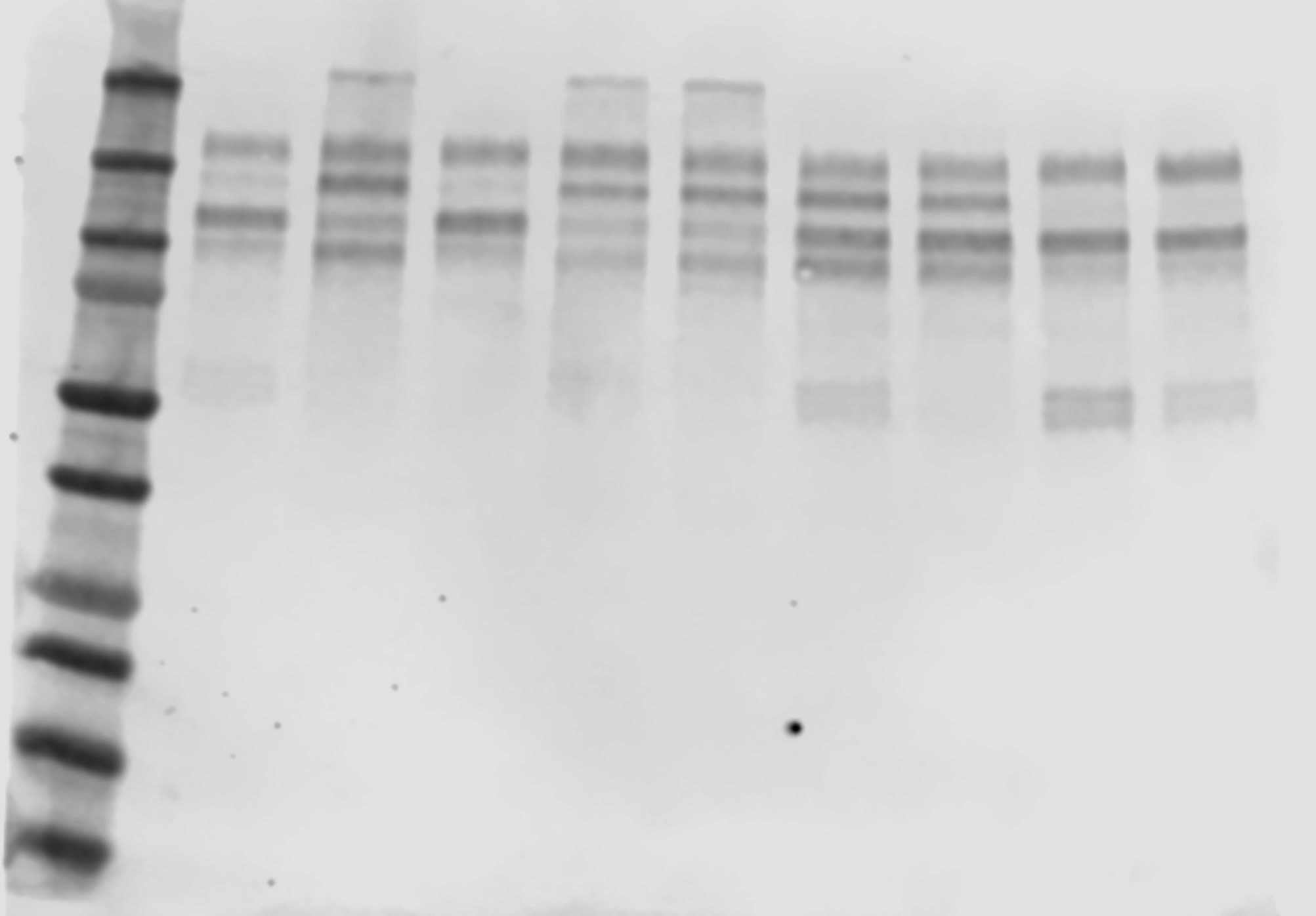

Supplement: Figure 5—source data 1. [file elife-63678-fig5-data1.zip › Figure 5 - Source Data 1/Fig5B - IP RON - PY.tif]

Figure 5A - Source Data

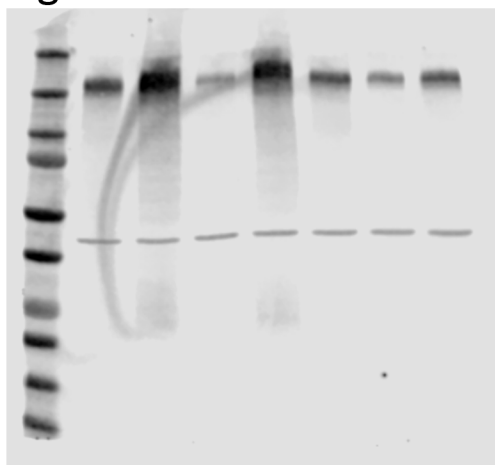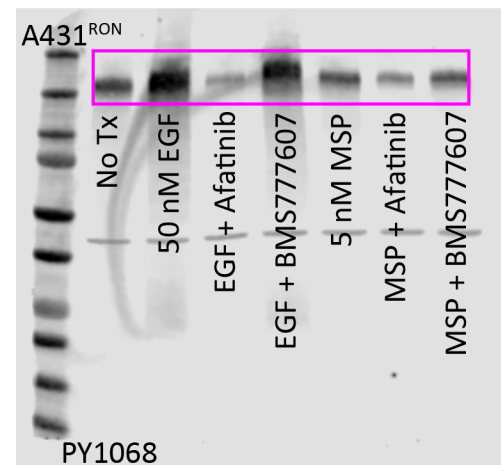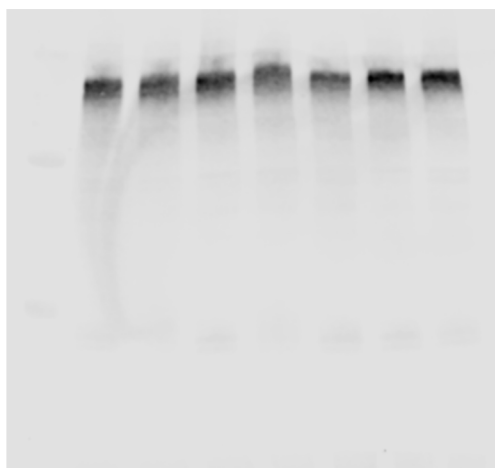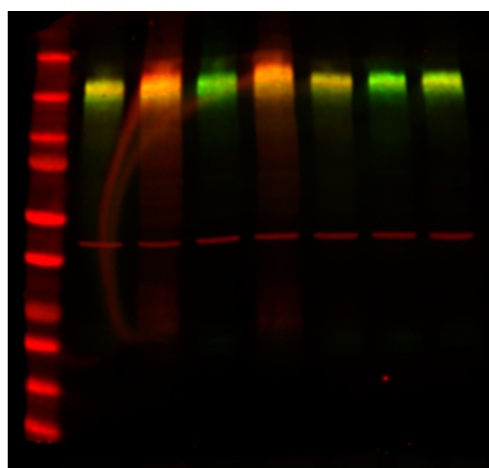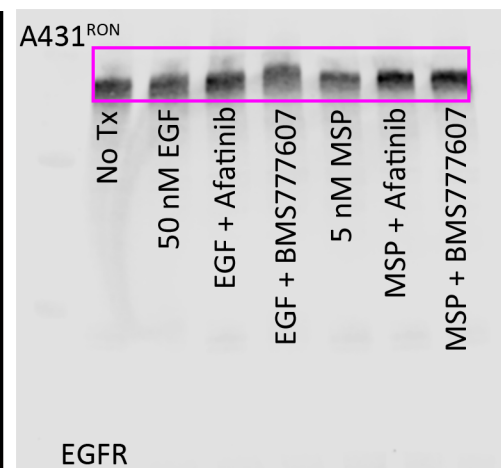

Figure 5B - Source Data

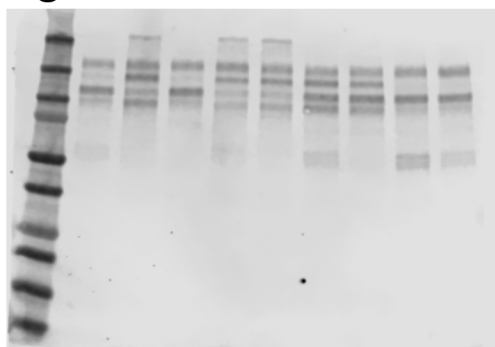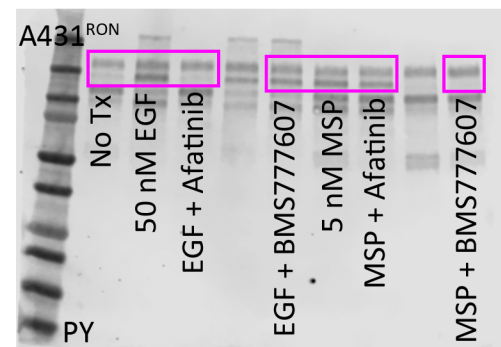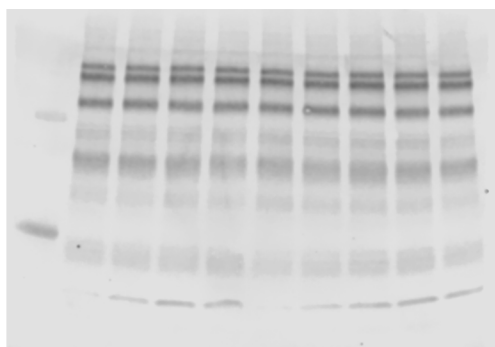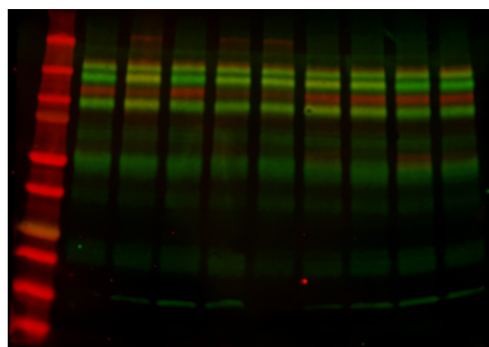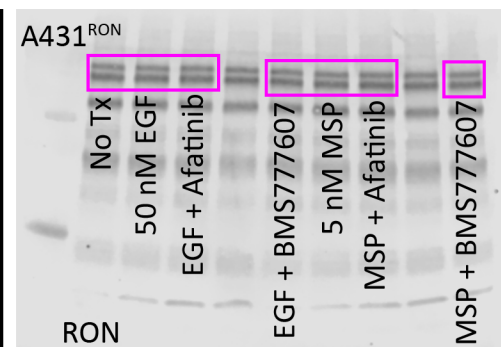

Supplement: Figure 5—source data 1. [file elife-63678-fig5-data1.zip › Figure 5 - Source Data 1/Figure 5 - Source Data 1 - Annotated.pdf]

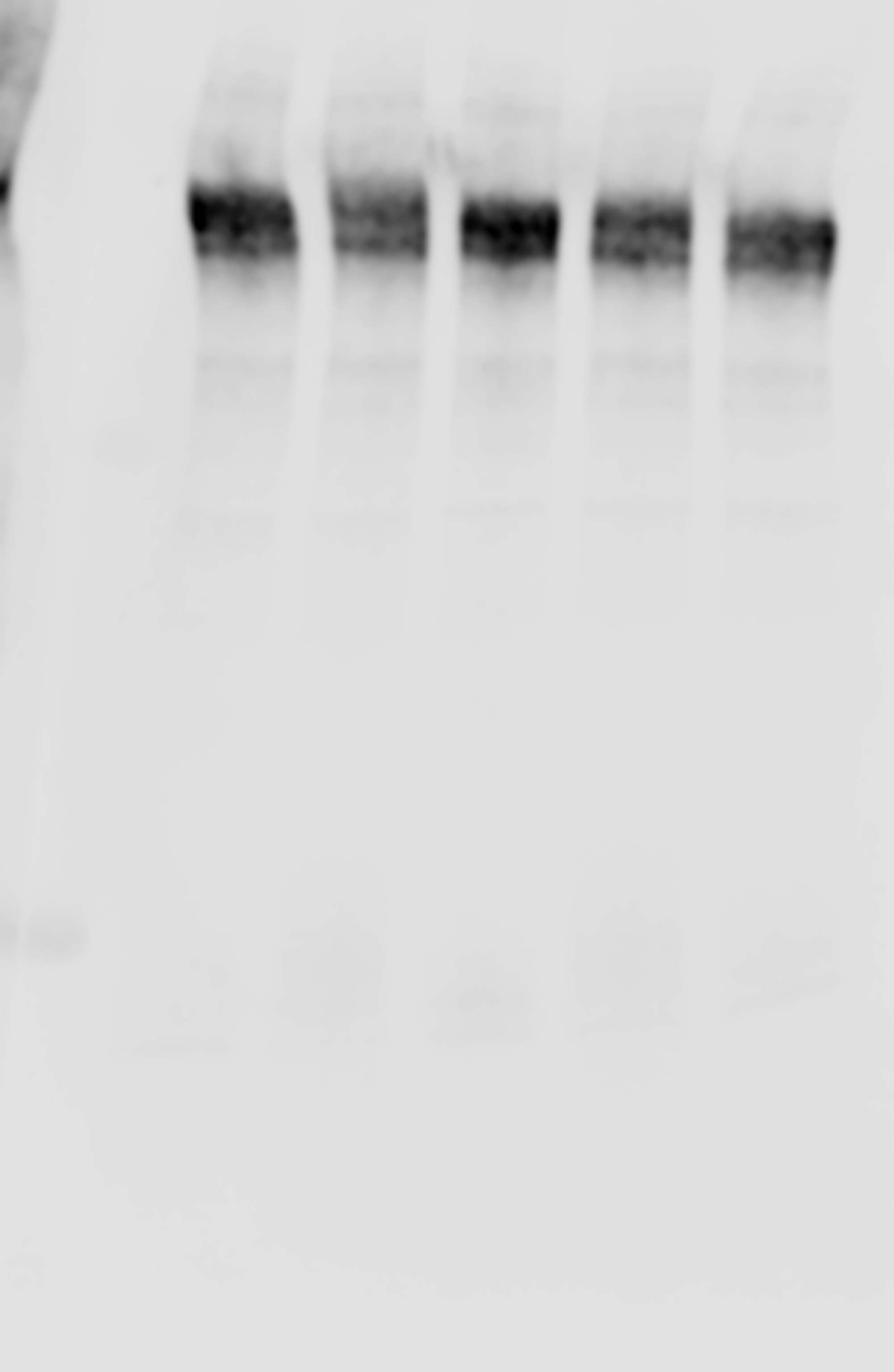

Supplement: Figure 5—figure supplement 1—source data 1. [file elife-63678-fig5-figsupp1-data1.zip › Figure 5 - Figure Supplement 1 - Source Data 1/Fig5Sup1 - EGFR - Protein.tif]

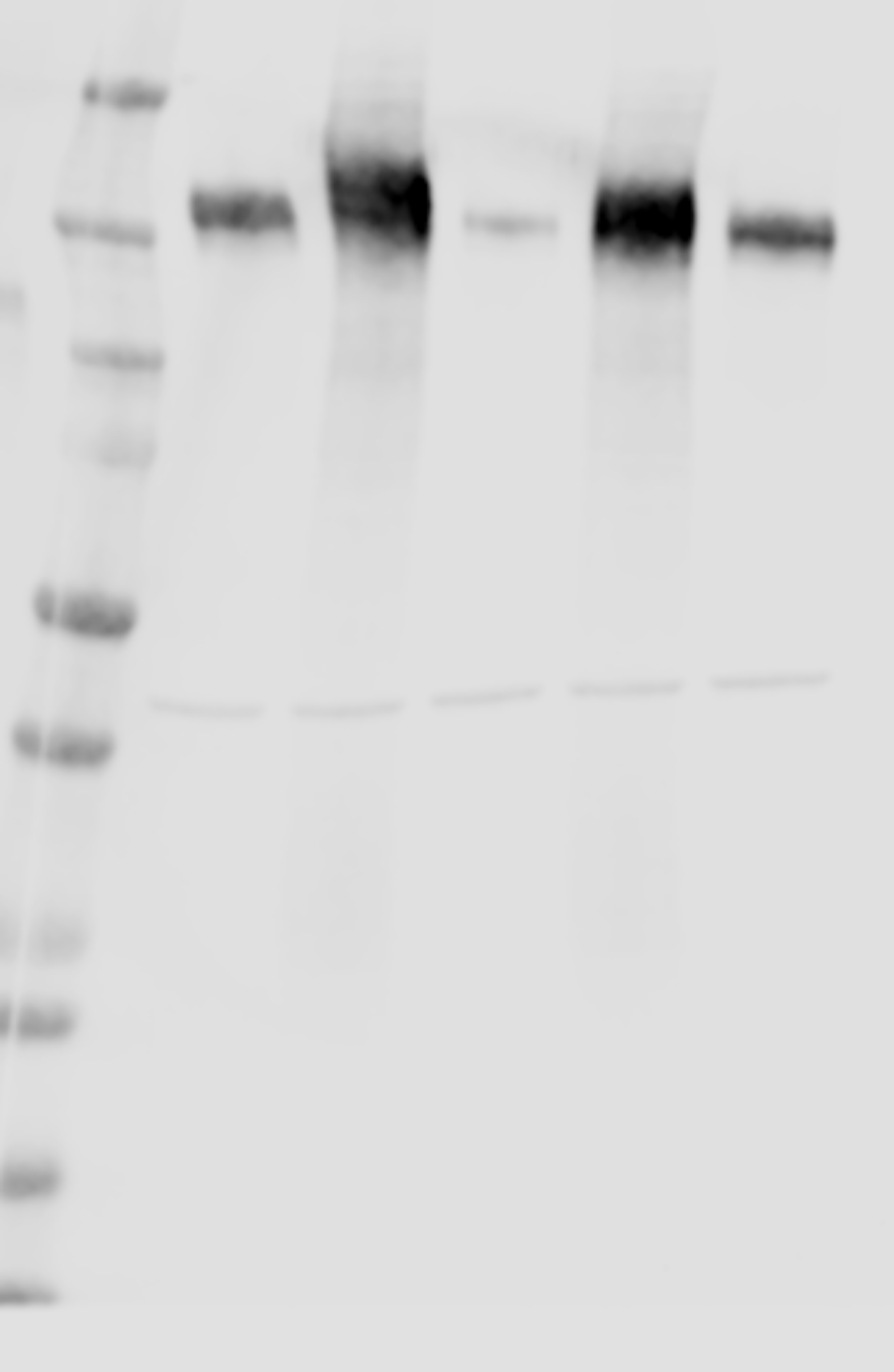

Supplement: Figure 5—figure supplement 1—source data 1. [file elife-63678-fig5-figsupp1-data1.zip › Figure 5 - Figure Supplement 1 - Source Data 1/Fig5Sup1 - EGFR - PY.tif]

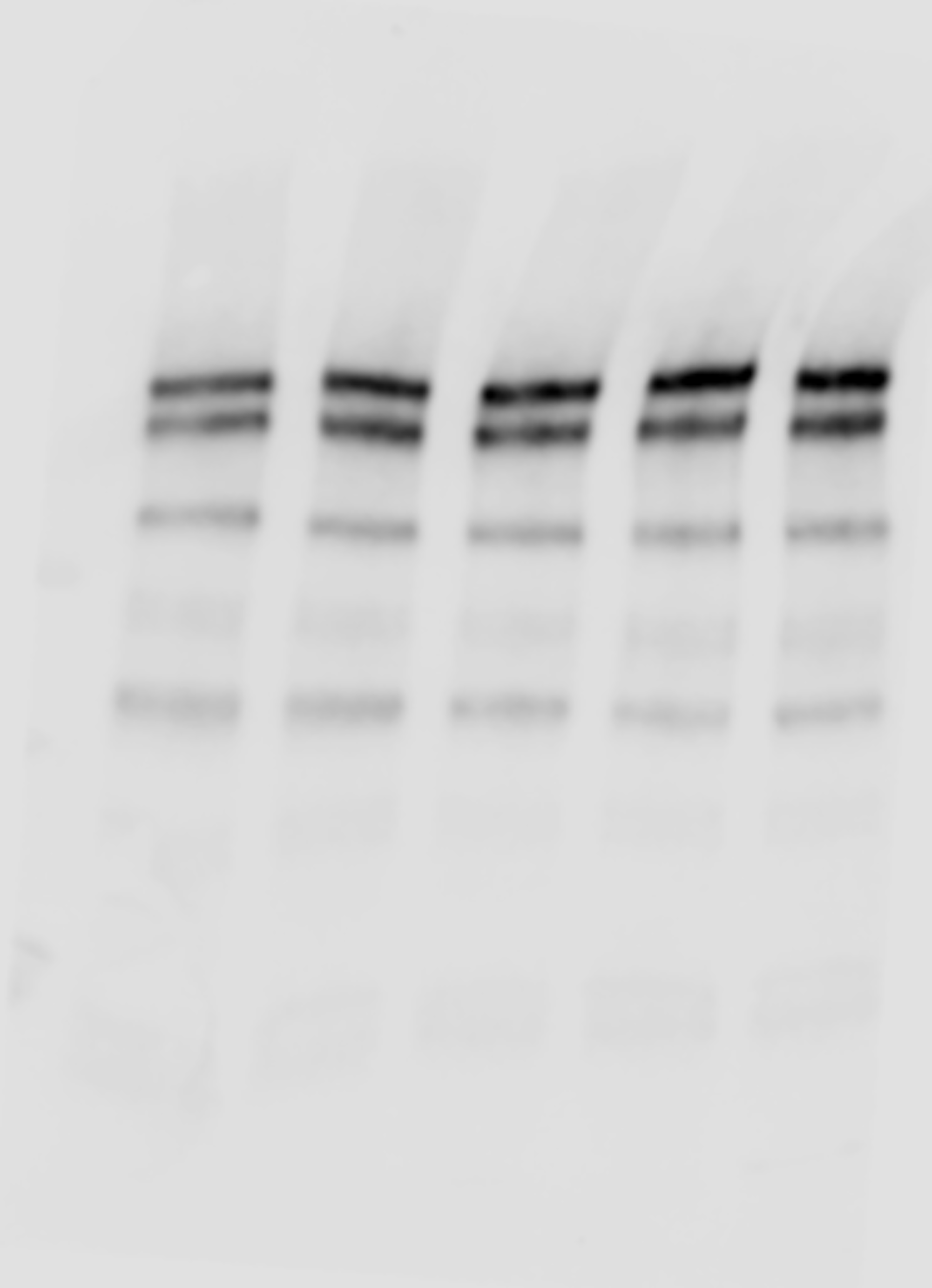

Supplement: Figure 5—figure supplement 1—source data 1. [file elife-63678-fig5-figsupp1-data1.zip › Figure 5 - Figure Supplement 1 - Source Data 1/Fig5Sup1 - RON IP - Protein.tif]

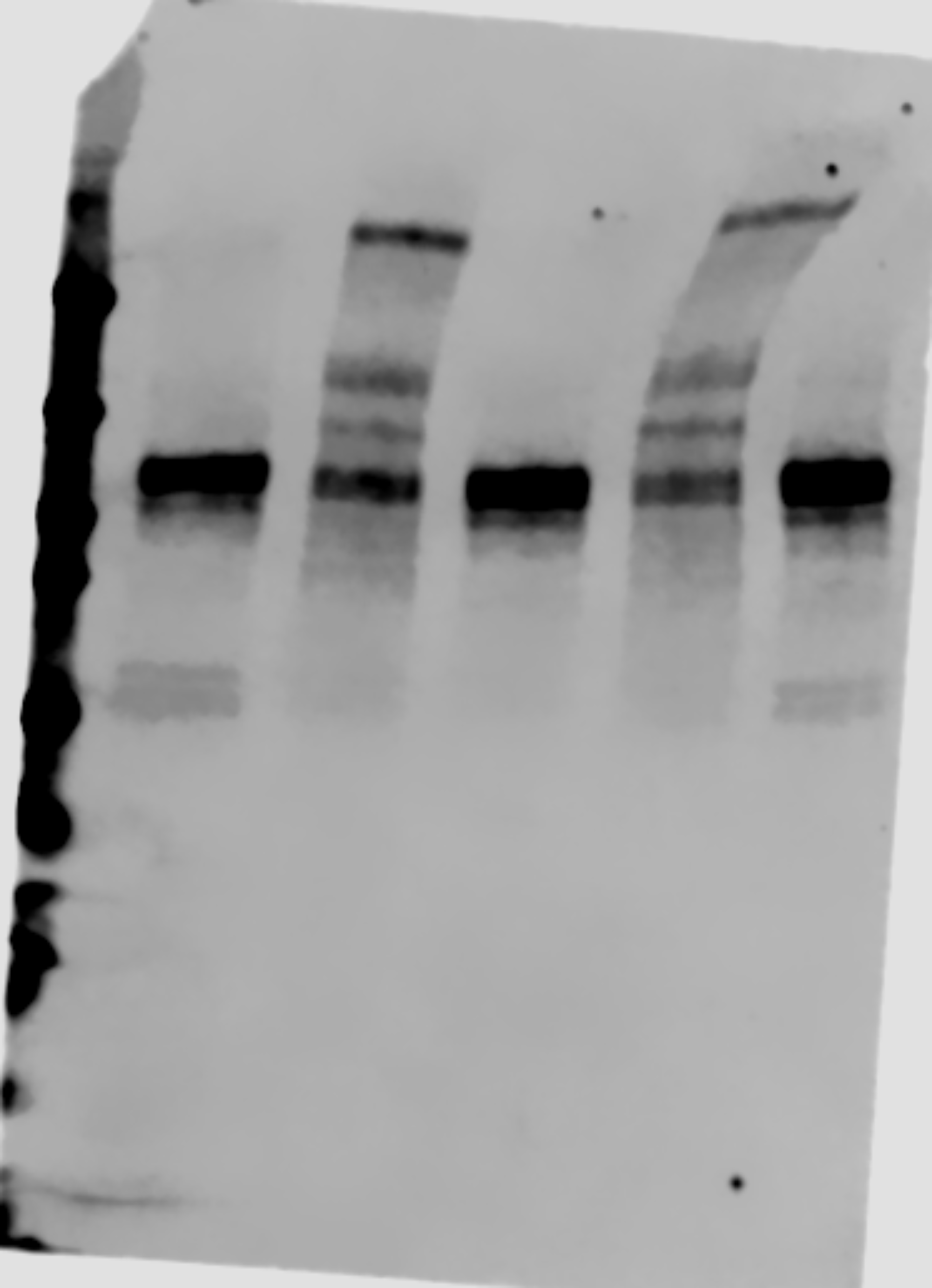

Supplement: Figure 5—figure supplement 1—source data 1. [file elife-63678-fig5-figsupp1-data1.zip › Figure 5 - Figure Supplement 1 - Source Data 1/Fig5Sup1 - RON IP - PY.tif]

Figure 5 - Figure Supplement 1 - Source Data 1

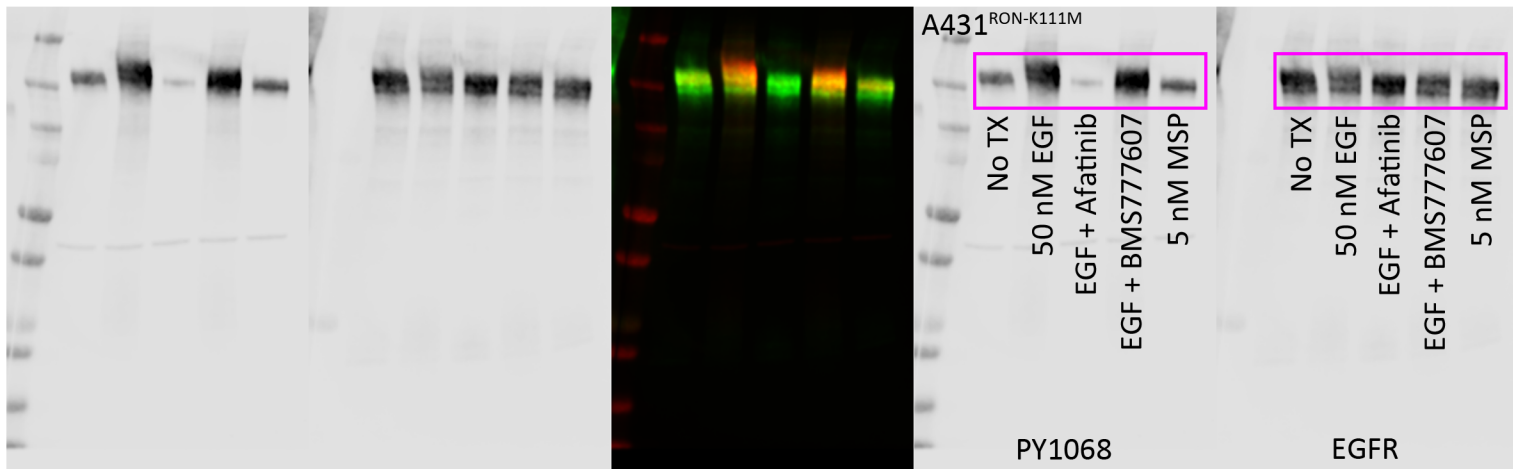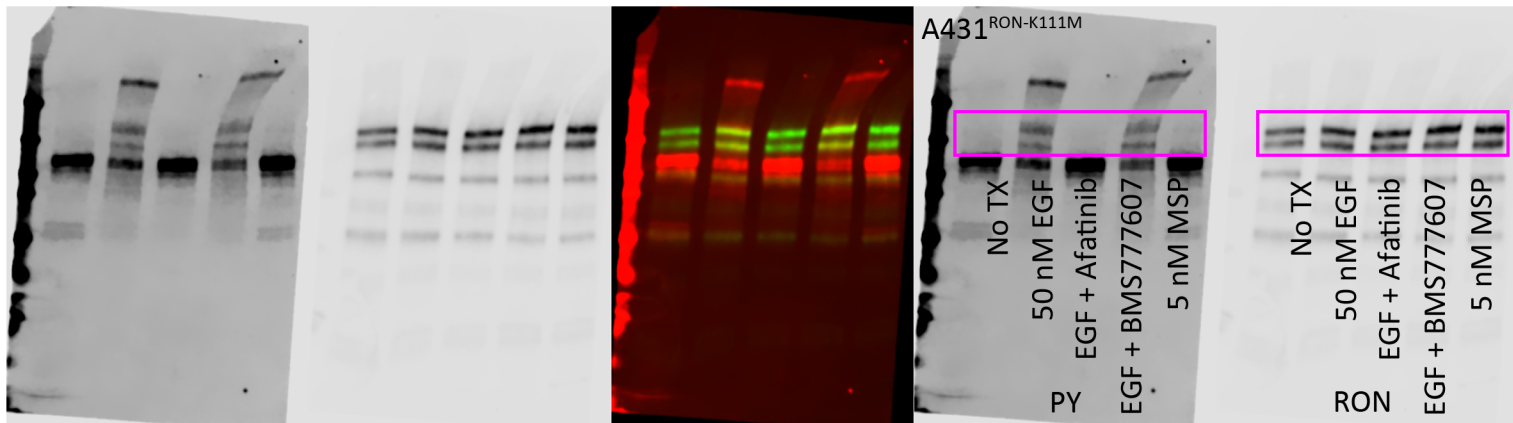

Supplement: Figure 5—figure supplement 1—source data 1. [file elife-63678-fig5-figsupp1-data1.zip › Figure 5 - Figure Supplement 1 - Source Data 1/Figure 5 - Figure Supplement 1 - Source Data 1 - Annotated.pdf]

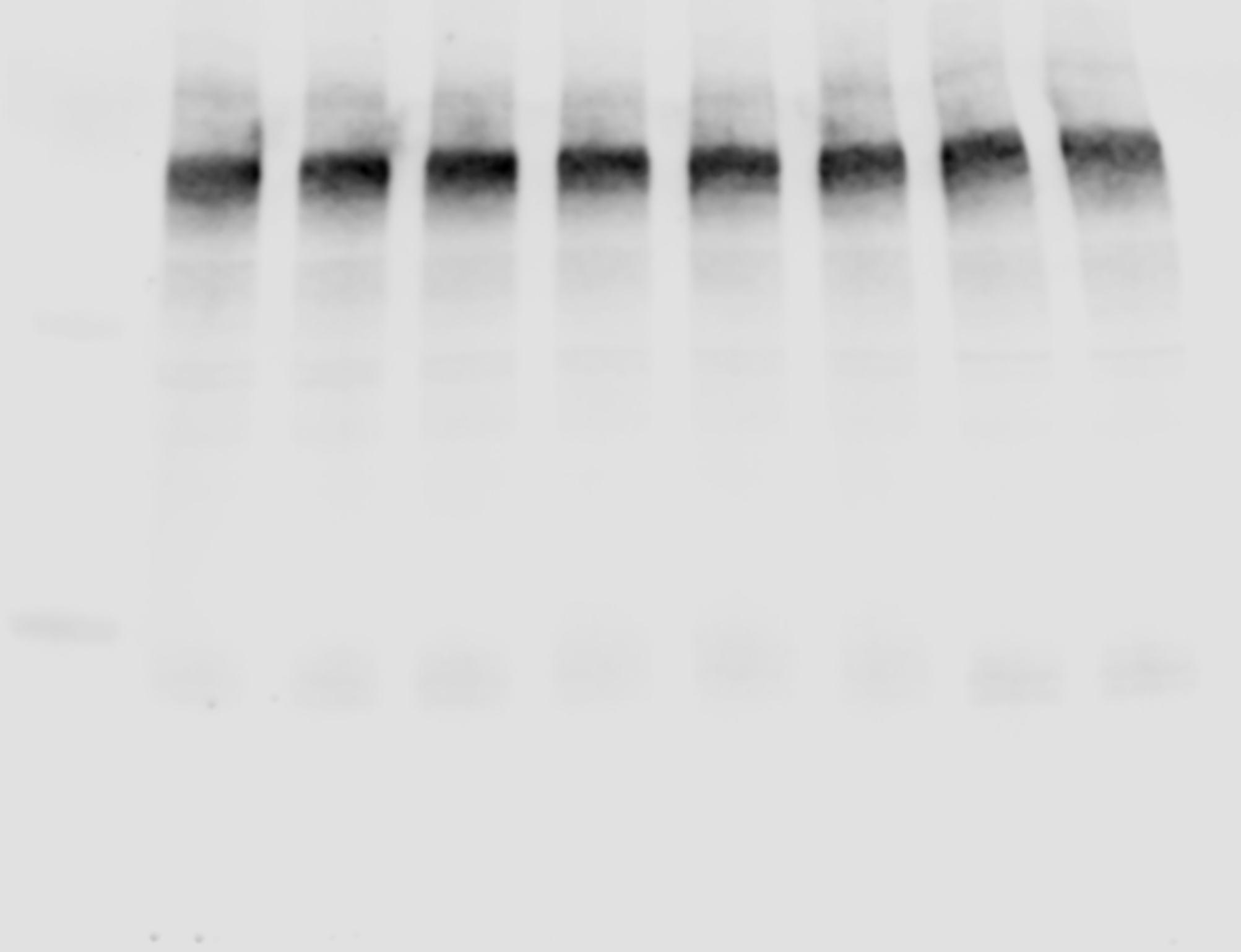

Supplement: Figure 6—source data 1. [file elife-63678-fig6-data1.zip › Figure 6 - Source Data 1/Fig6A - EGFR - Protein.tif]

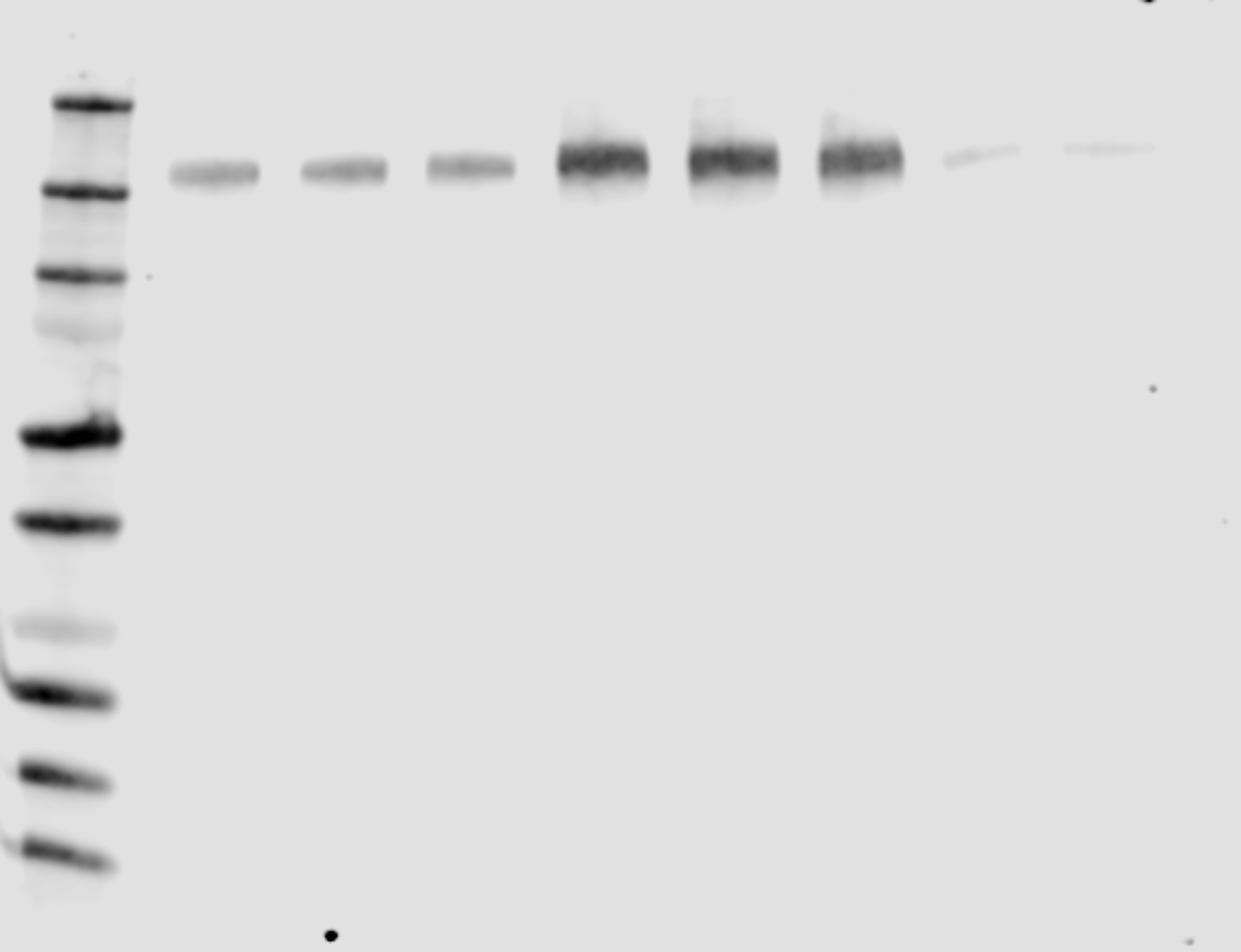

Supplement: Figure 6—source data 1. [file elife-63678-fig6-data1.zip › Figure 6 - Source Data 1/Fig6A - EGFR - PY.tif]

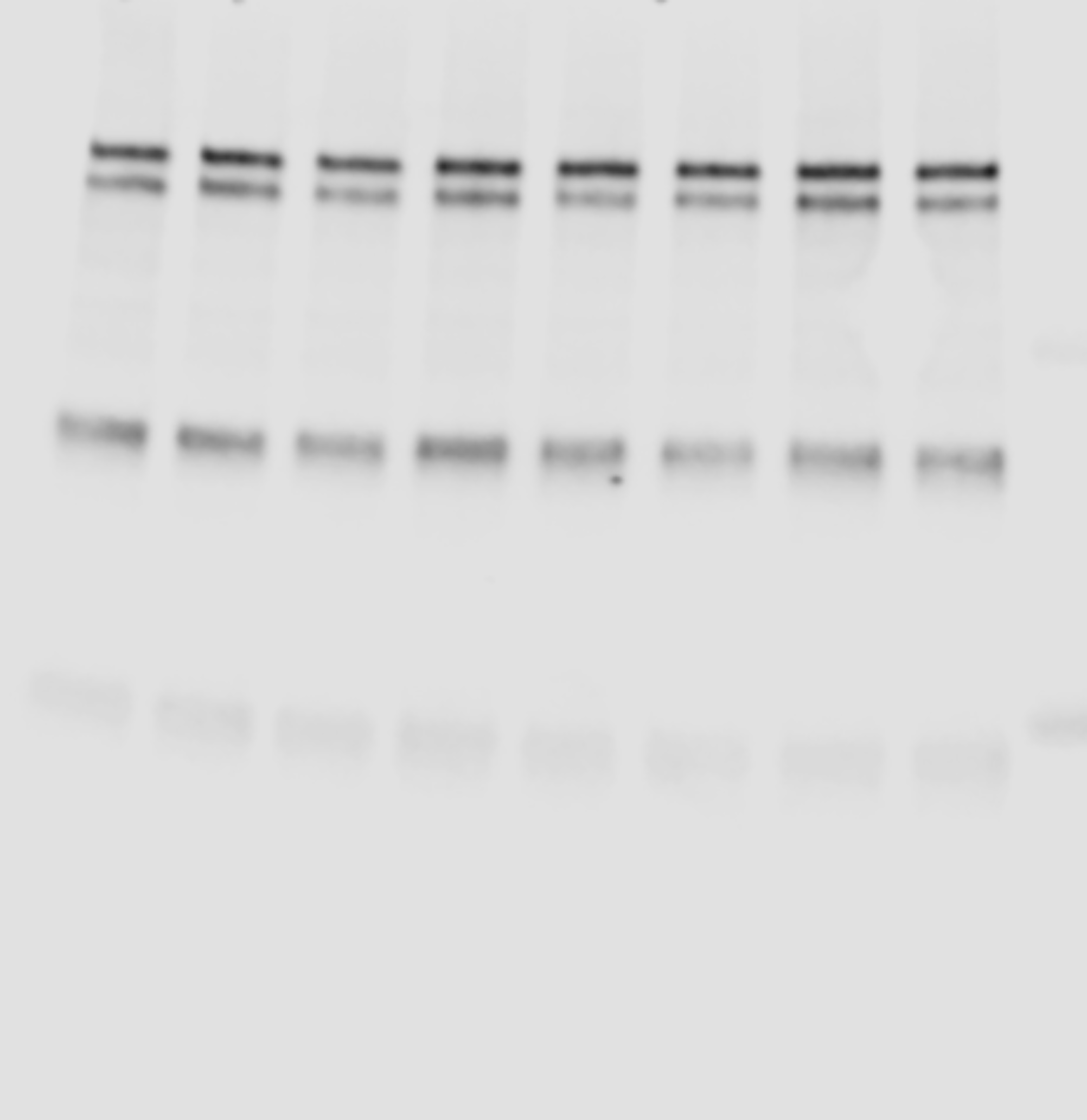

Supplement: Figure 6—source data 1. [file elife-63678-fig6-data1.zip › Figure 6 - Source Data 1/Fig6A - RON IP - Protein.tif]

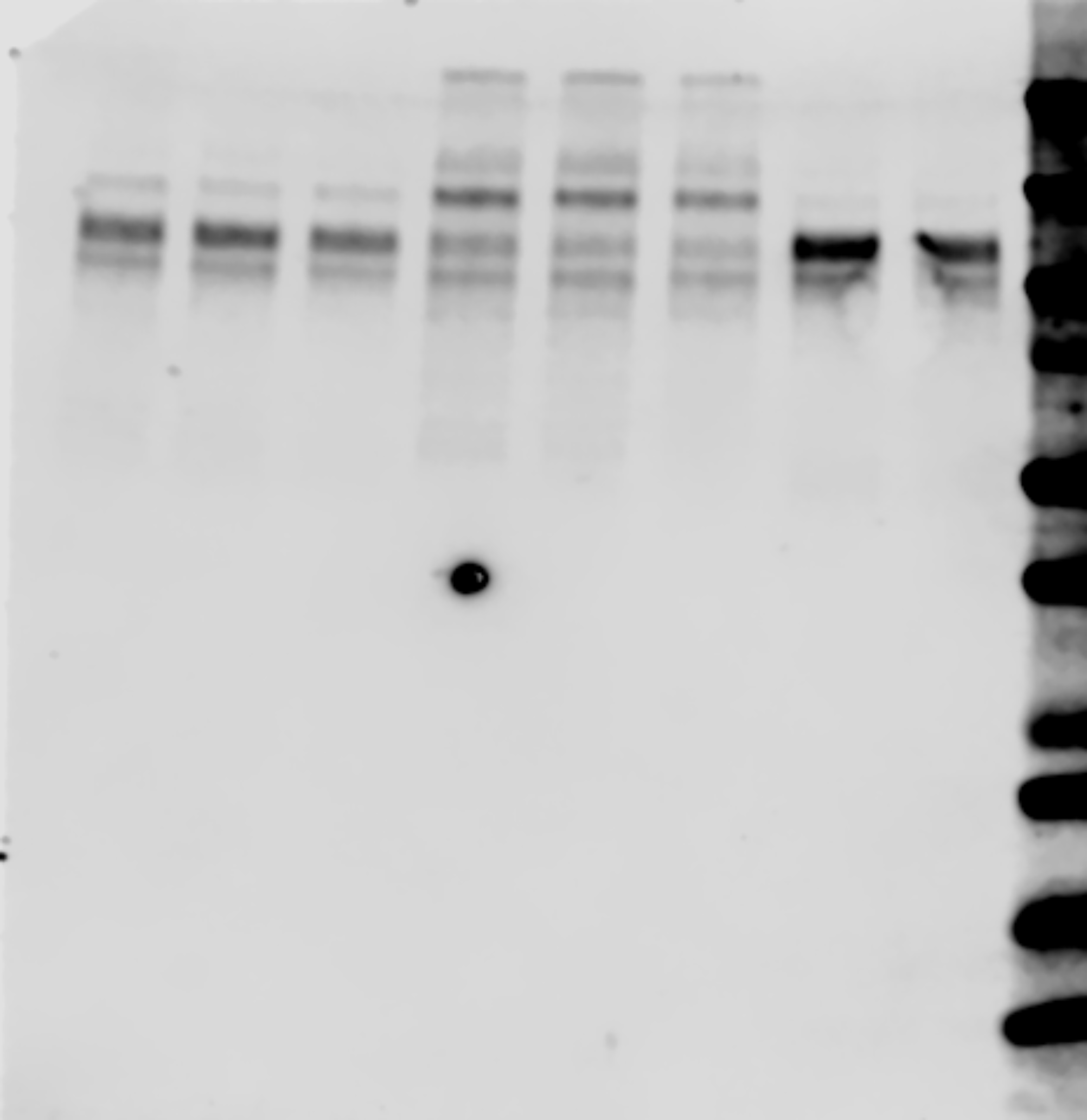

Supplement: Figure 6—source data 1. [file elife-63678-fig6-data1.zip › Figure 6 - Source Data 1/Fig6A - RON IP - PY.tif]

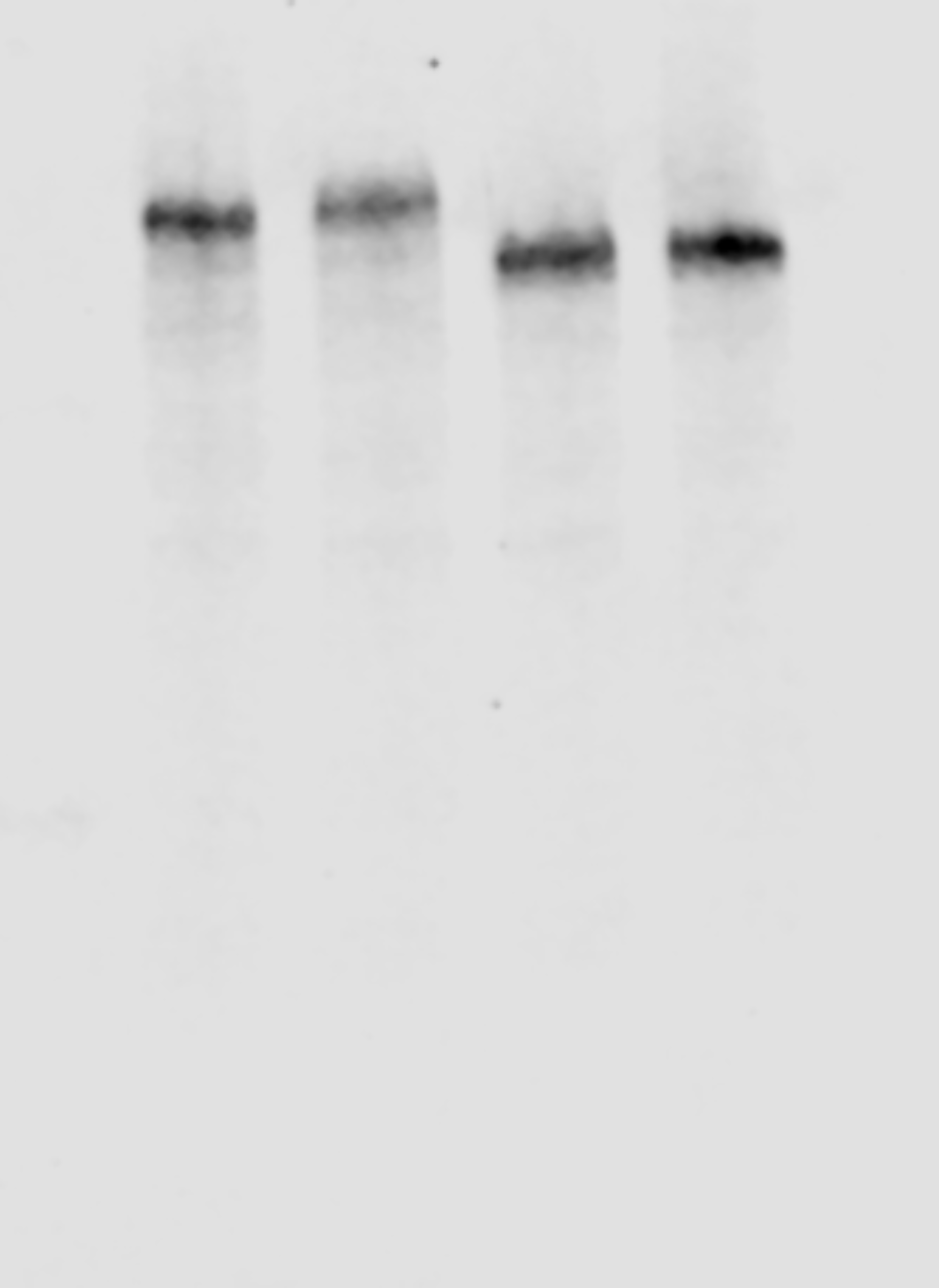

Supplement: Figure 6—source data 1. [file elife-63678-fig6-data1.zip › Figure 6 - Source Data 1/Fig6B - EGFR - inset.tif]

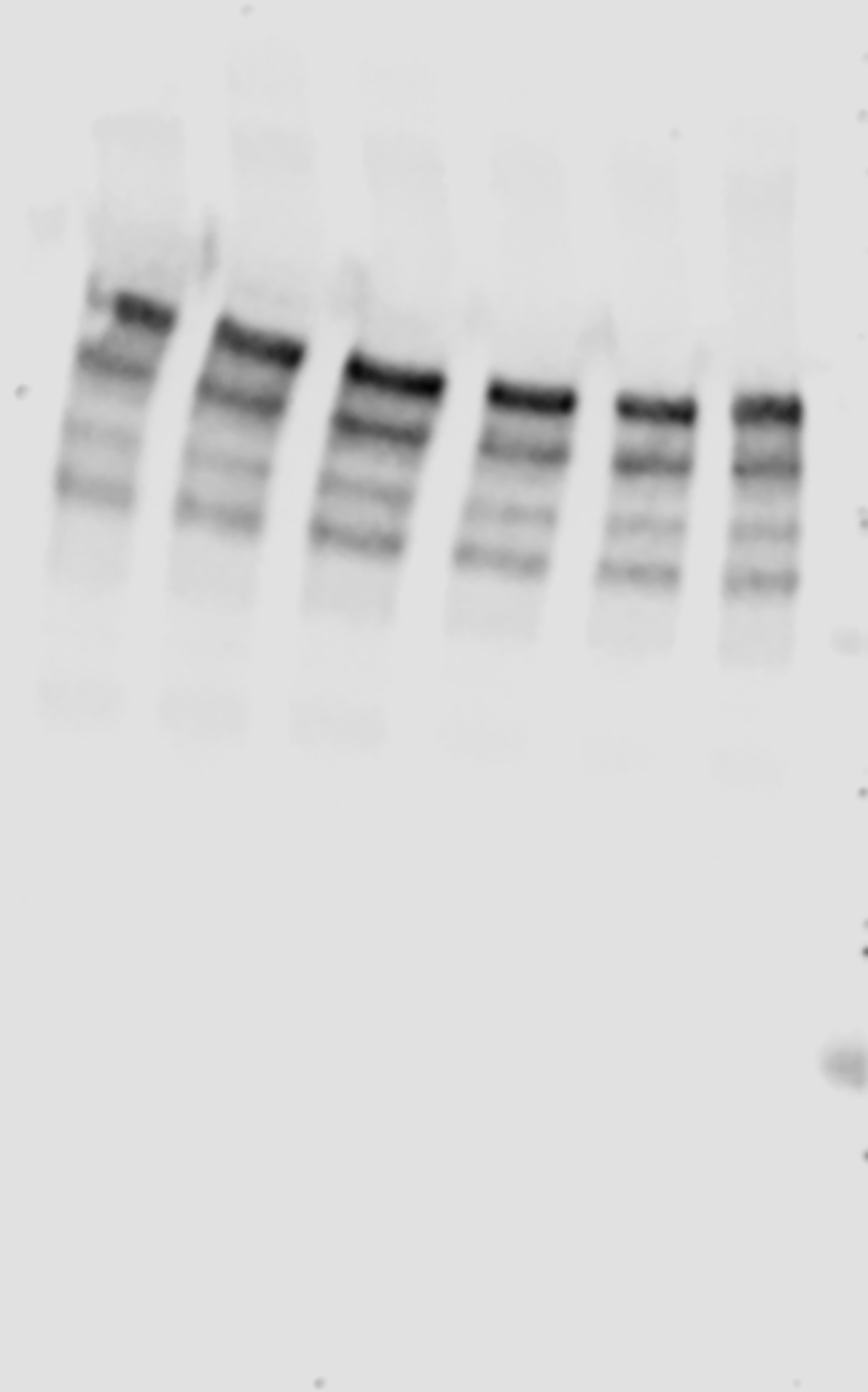

Supplement: Figure 6—source data 1. [file elife-63678-fig6-data1.zip › Figure 6 - Source Data 1/Fig6B - RON IP - Protein.tif]

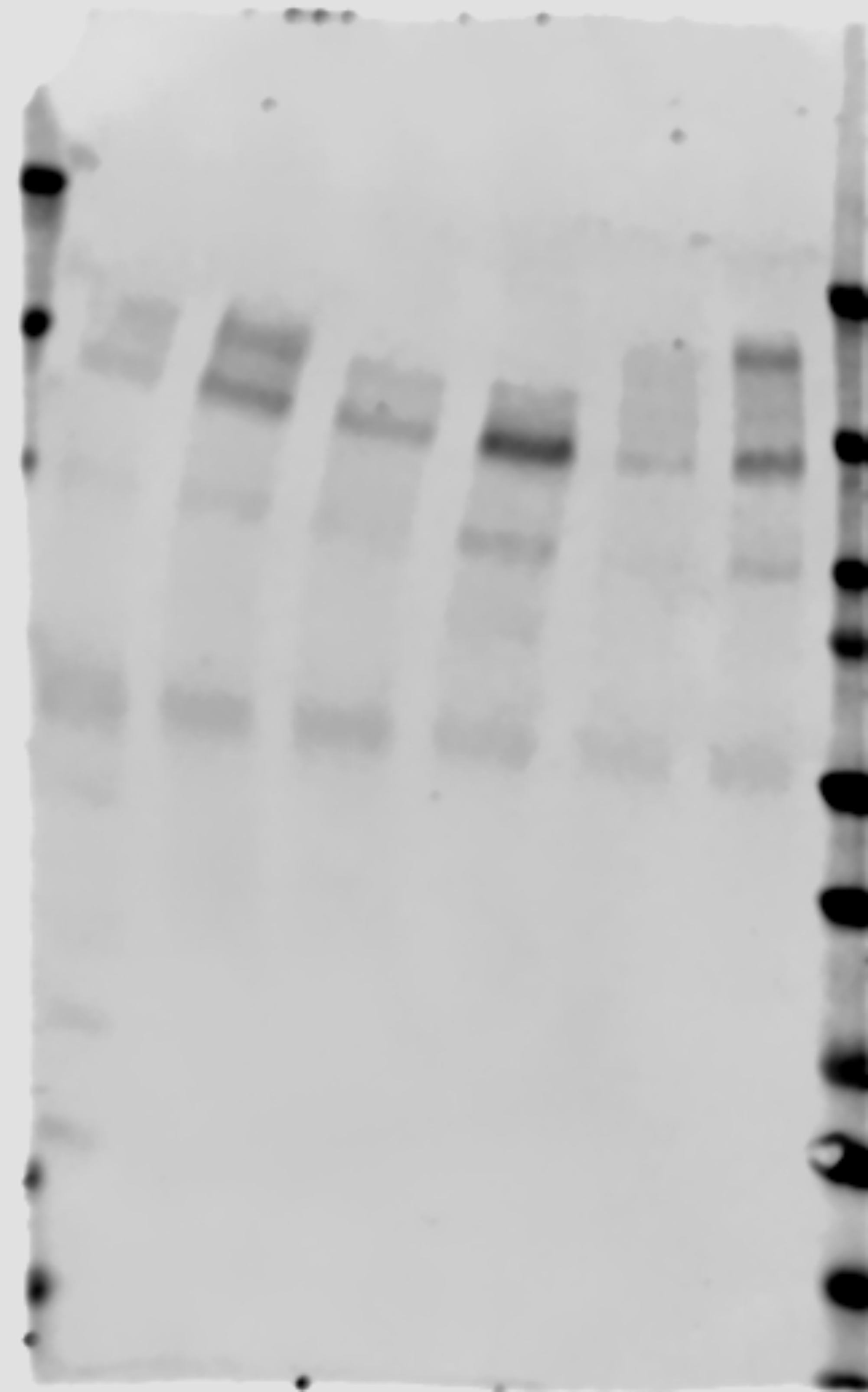

Supplement: Figure 6—source data 1. [file elife-63678-fig6-data1.zip › Figure 6 - Source Data 1/Fig6B - RON IP - PY.tif]

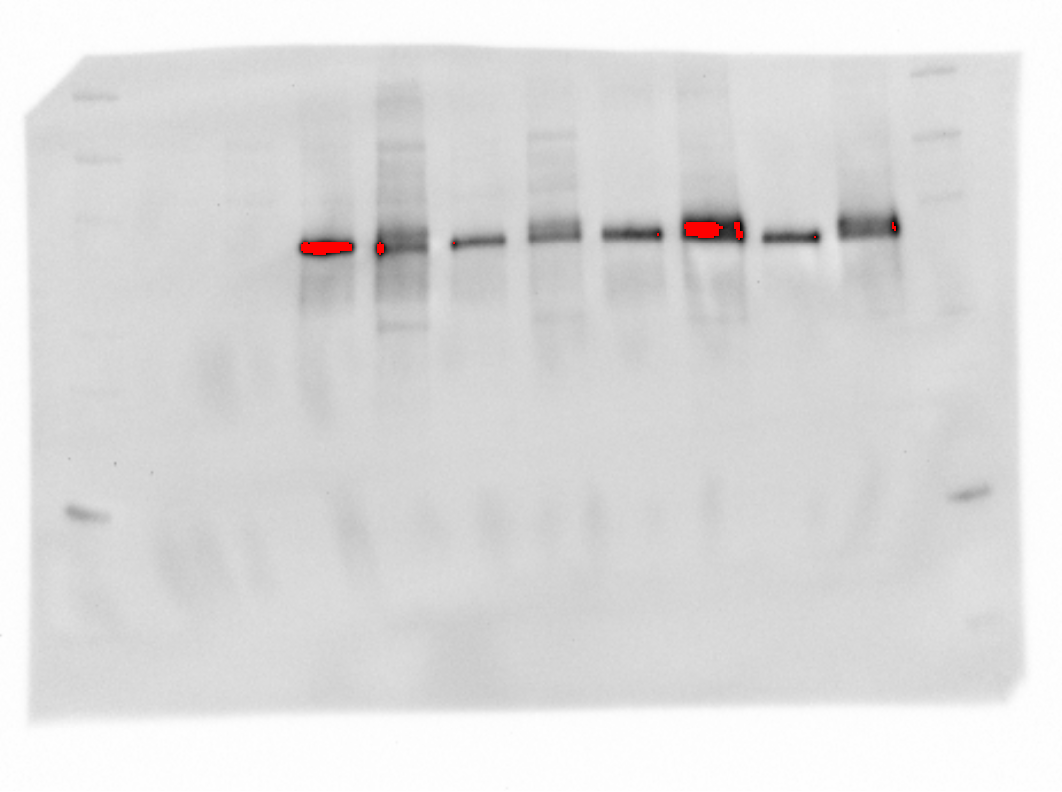

Supplement: Figure 6—source data 1. [file elife-63678-fig6-data1.zip › Figure 6 - Source Data 1/Fig6C - EGFR - Protein.tif]

Figure 6A - Source Data

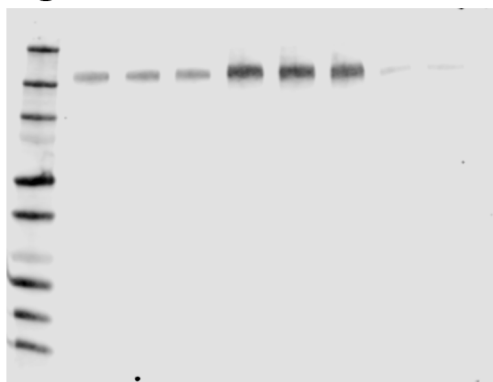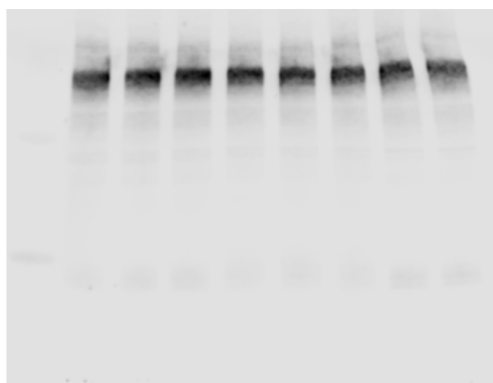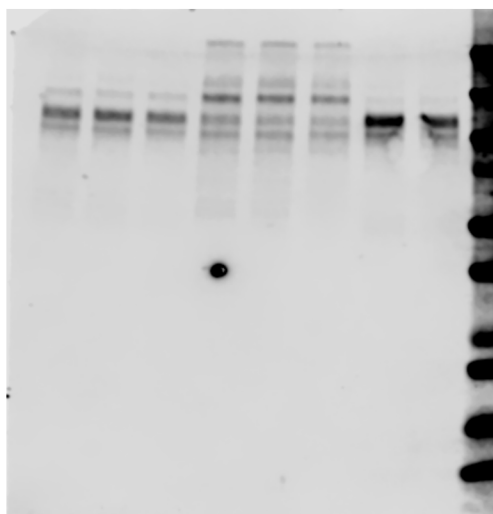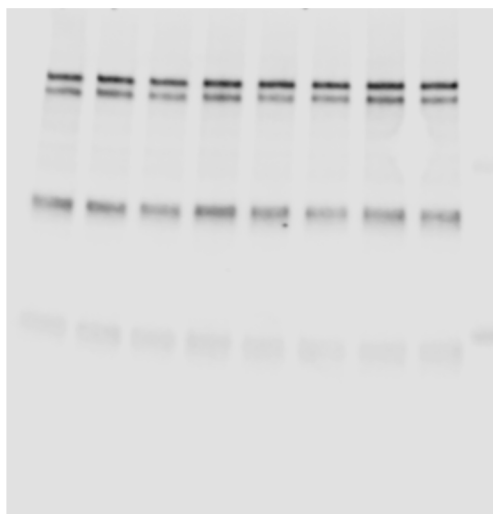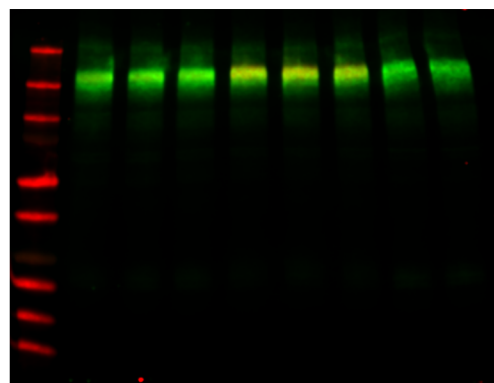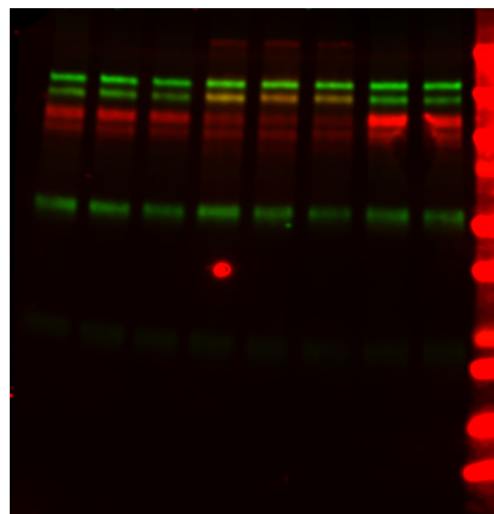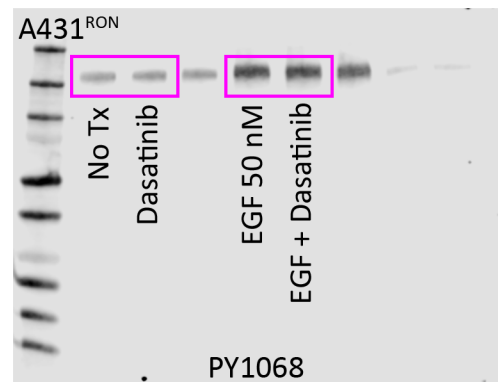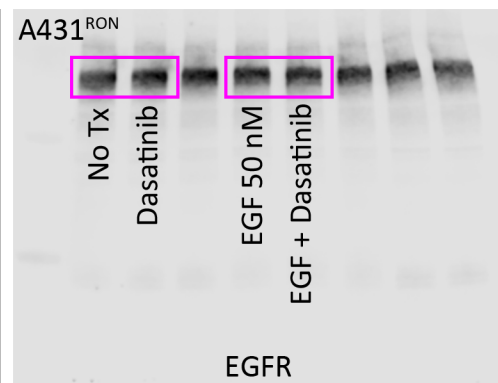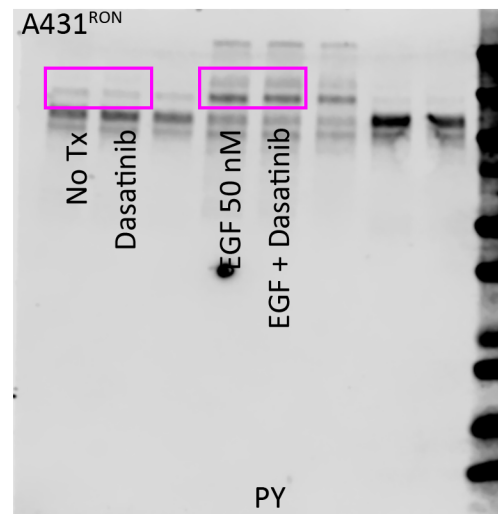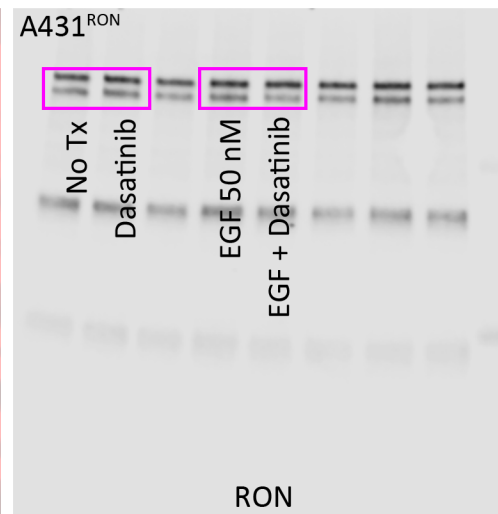

Figure 6B - Source Data

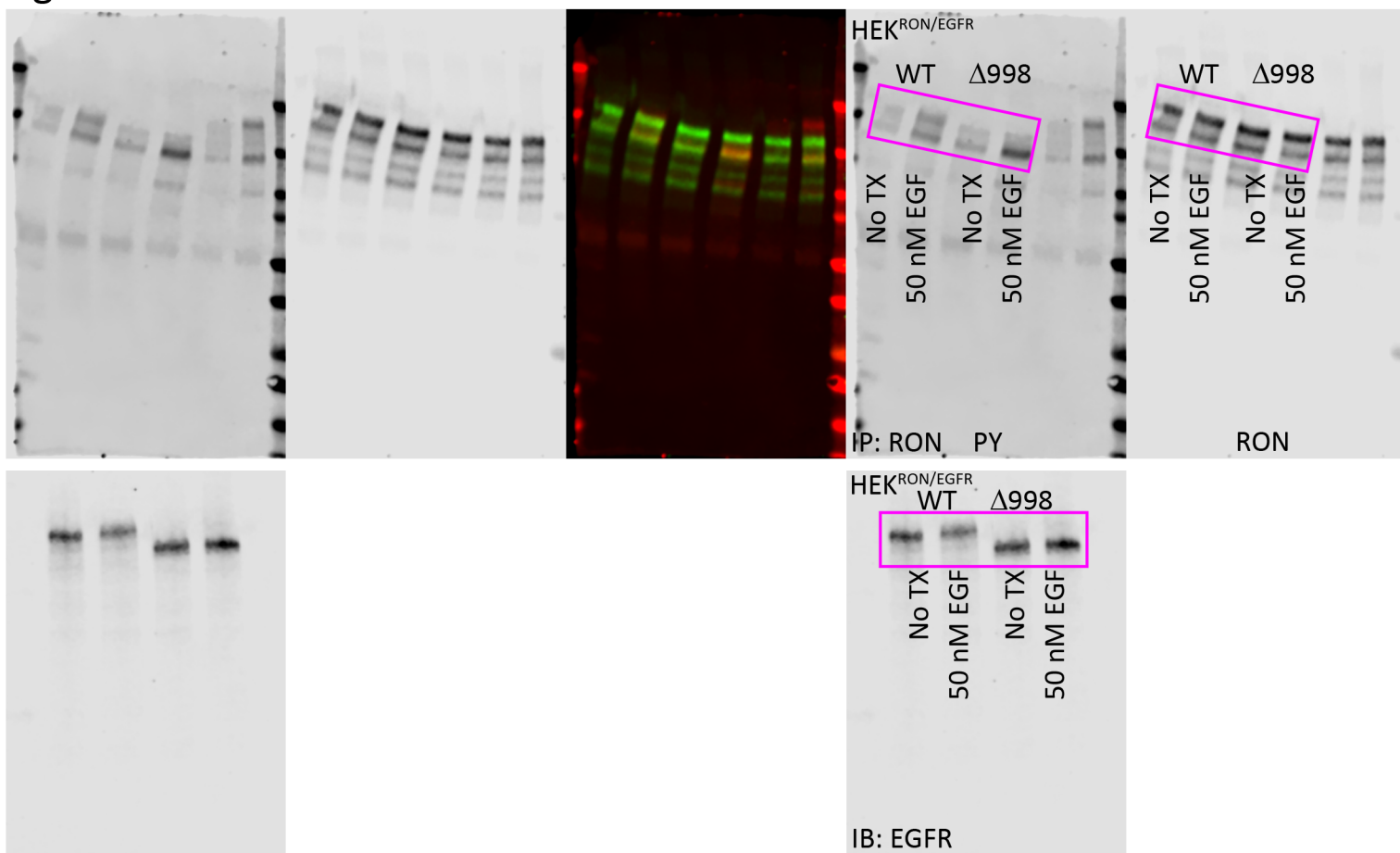

Figure 6C - Source Data

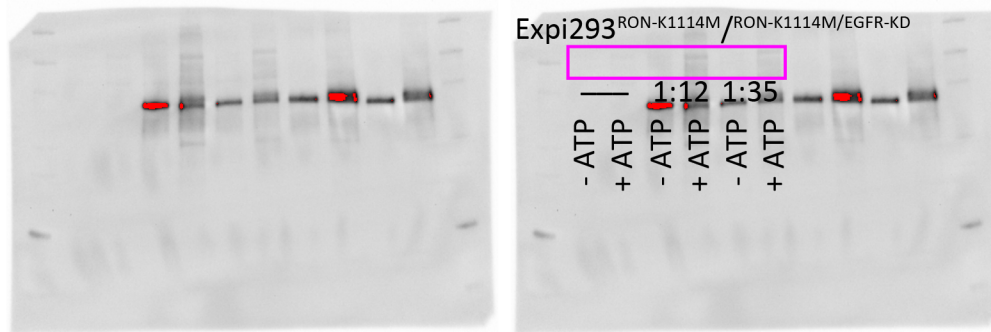

Supplement: Figure 6—source data 1. [file elife-63678-fig6-data1.zip › Figure 6 - Source Data 1/Figure 6 - Source Data 1 - Annotated.pdf]

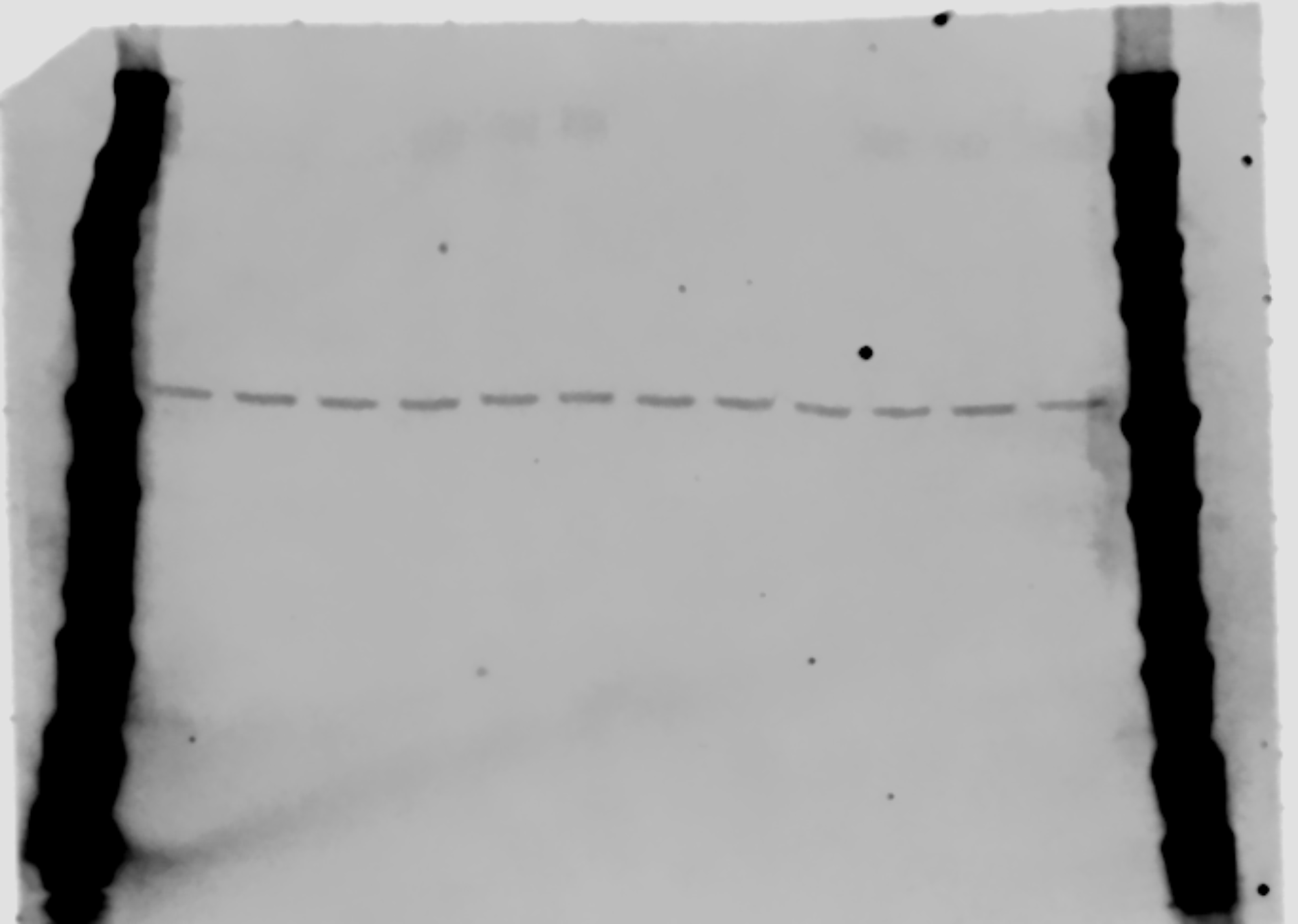

Supplement: Figure 6—figure supplement 1—source data 1. [file elife-63678-fig6-figsupp1-data1.zip › Figure 6 - Figure Supplement 1 - Source Data 1/Fig6Sup1 - Src - Protein.tif]

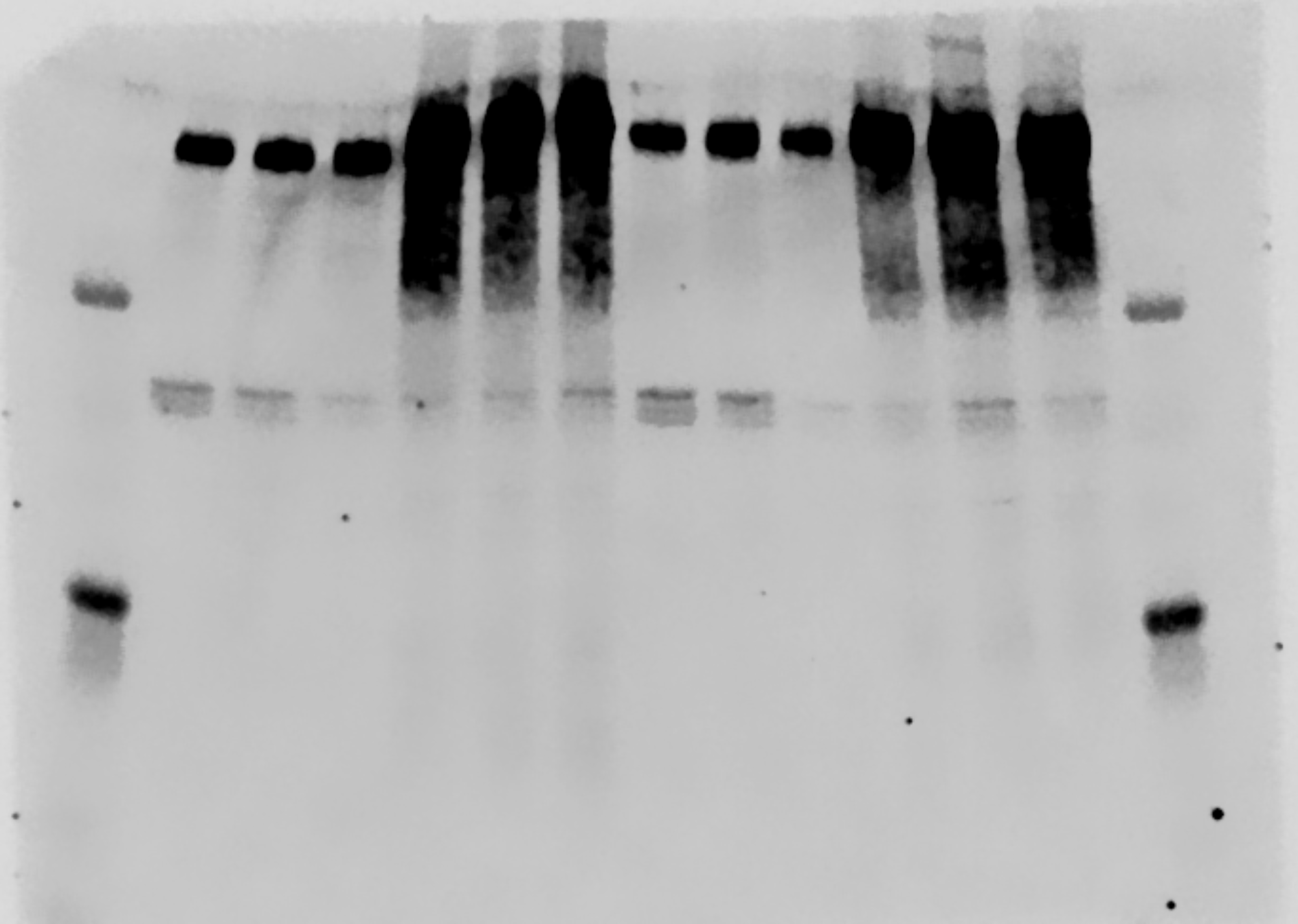

Supplement: Figure 6—figure supplement 1—source data 1. [file elife-63678-fig6-figsupp1-data1.zip › Figure 6 - Figure Supplement 1 - Source Data 1/Fig6Sup1 - Src - PY.tif]

Figure 6 - Figure Supplement 1 - Source Data 1

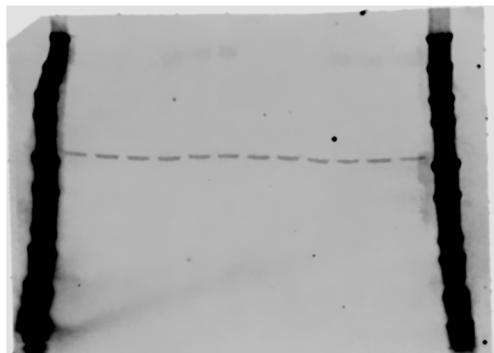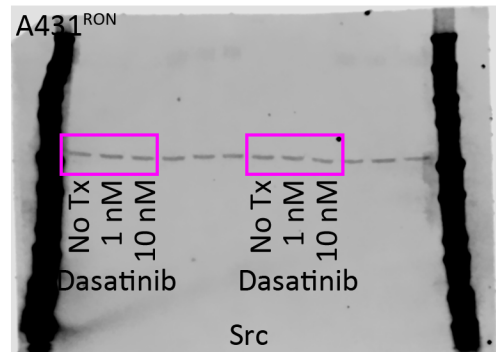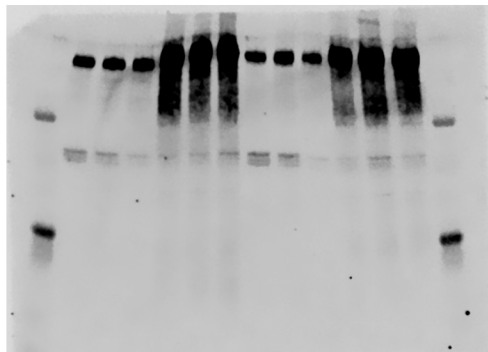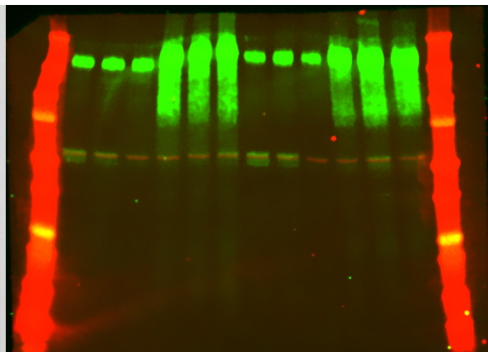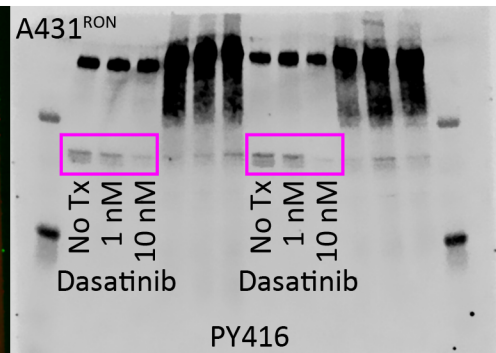

Supplement: Figure 6—figure supplement 1—source data 1. [file elife-63678-fig6-figsupp1-data1.zip › Figure 6 - Figure Supplement 1 - Source Data 1/Figure 6 - Figure Supplement 1 - Source Data 1 - Annotated.pdf]

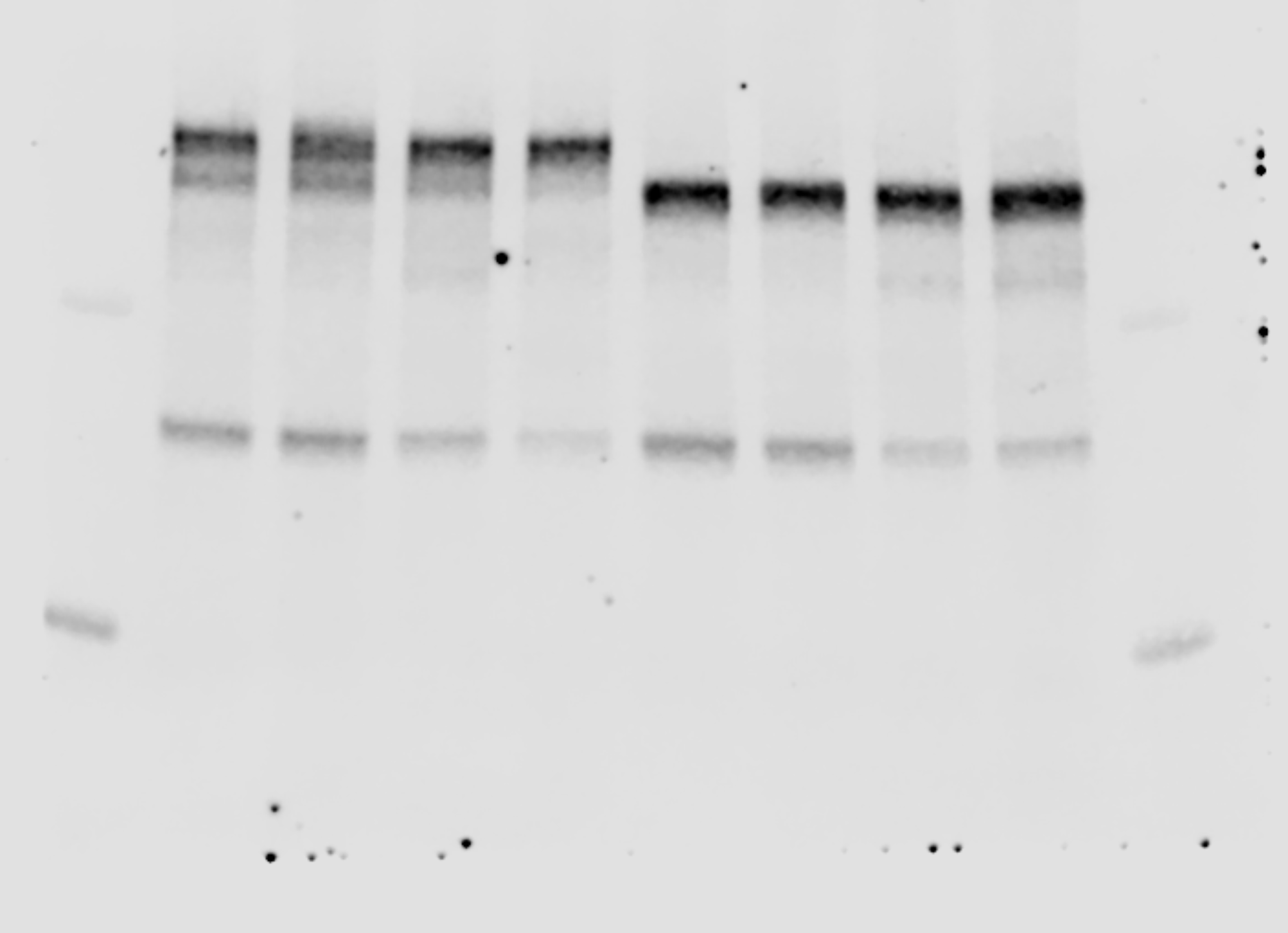

Supplement: Figure 6—figure supplement 2—source data 1. [file elife-63678-fig6-figsupp2-data1.zip › Figure 6 - Figure Supplement 2 - Source Data 1/Fig6Sup2 - EGFR - Replicate1 - Protein.tif]

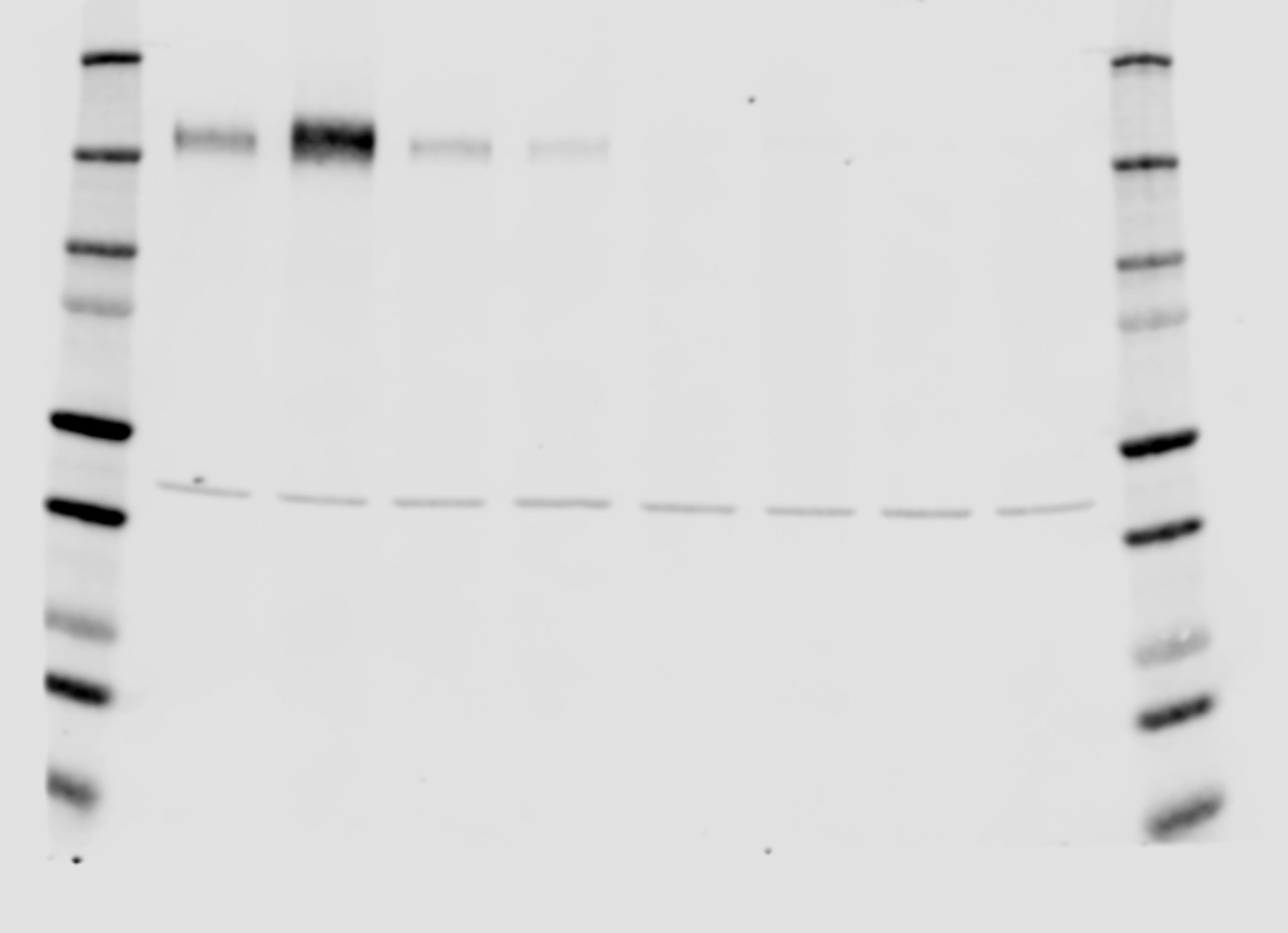

Supplement: Figure 6—figure supplement 2—source data 1. [file elife-63678-fig6-figsupp2-data1.zip › Figure 6 - Figure Supplement 2 - Source Data 1/Fig6Sup2 - EGFR - Replicate1 - PY.tif]

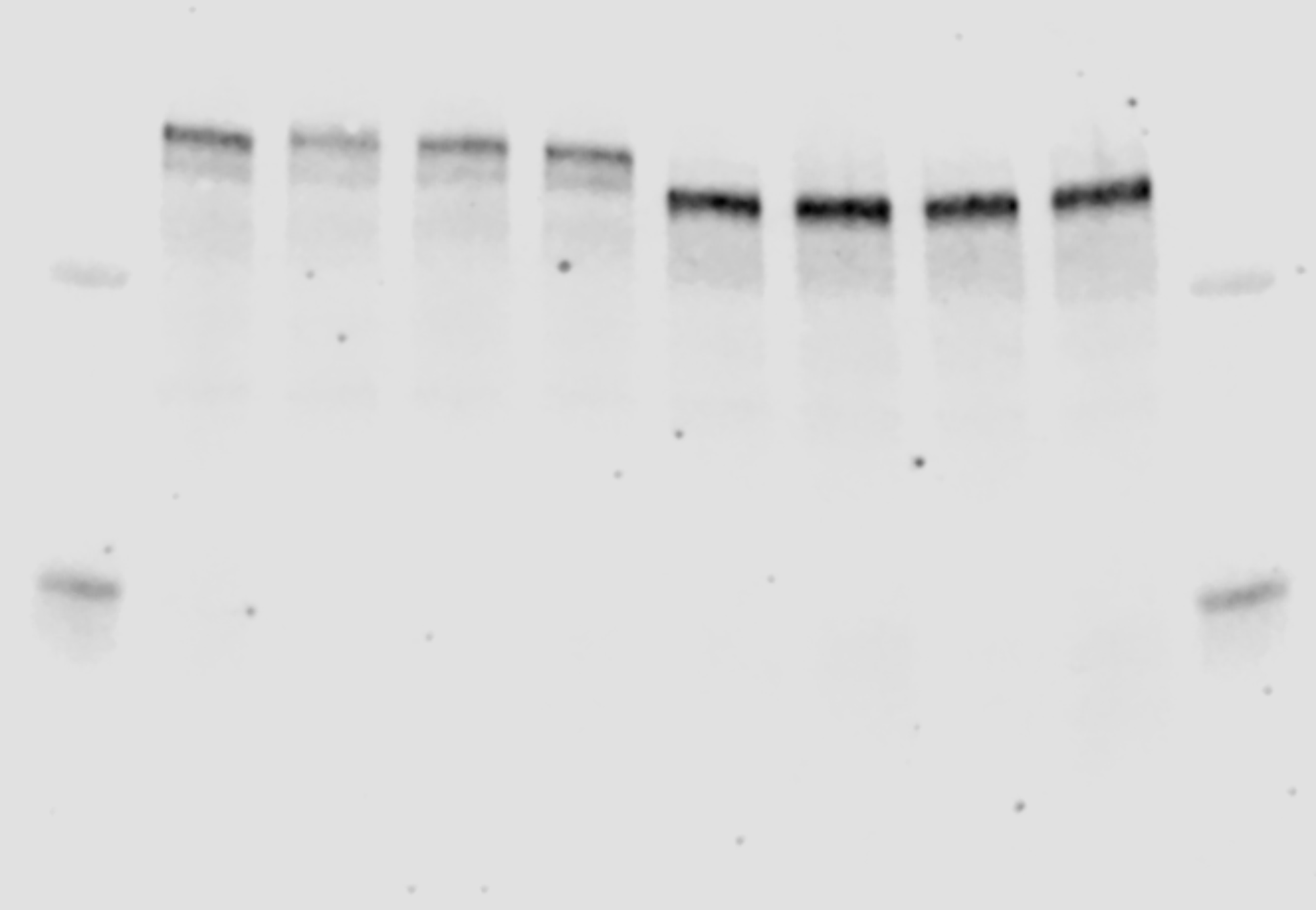

Supplement: Figure 6—figure supplement 2—source data 1. [file elife-63678-fig6-figsupp2-data1.zip › Figure 6 - Figure Supplement 2 - Source Data 1/Fig6Sup2 - EGFR - Replicate2 - Protein.tif]

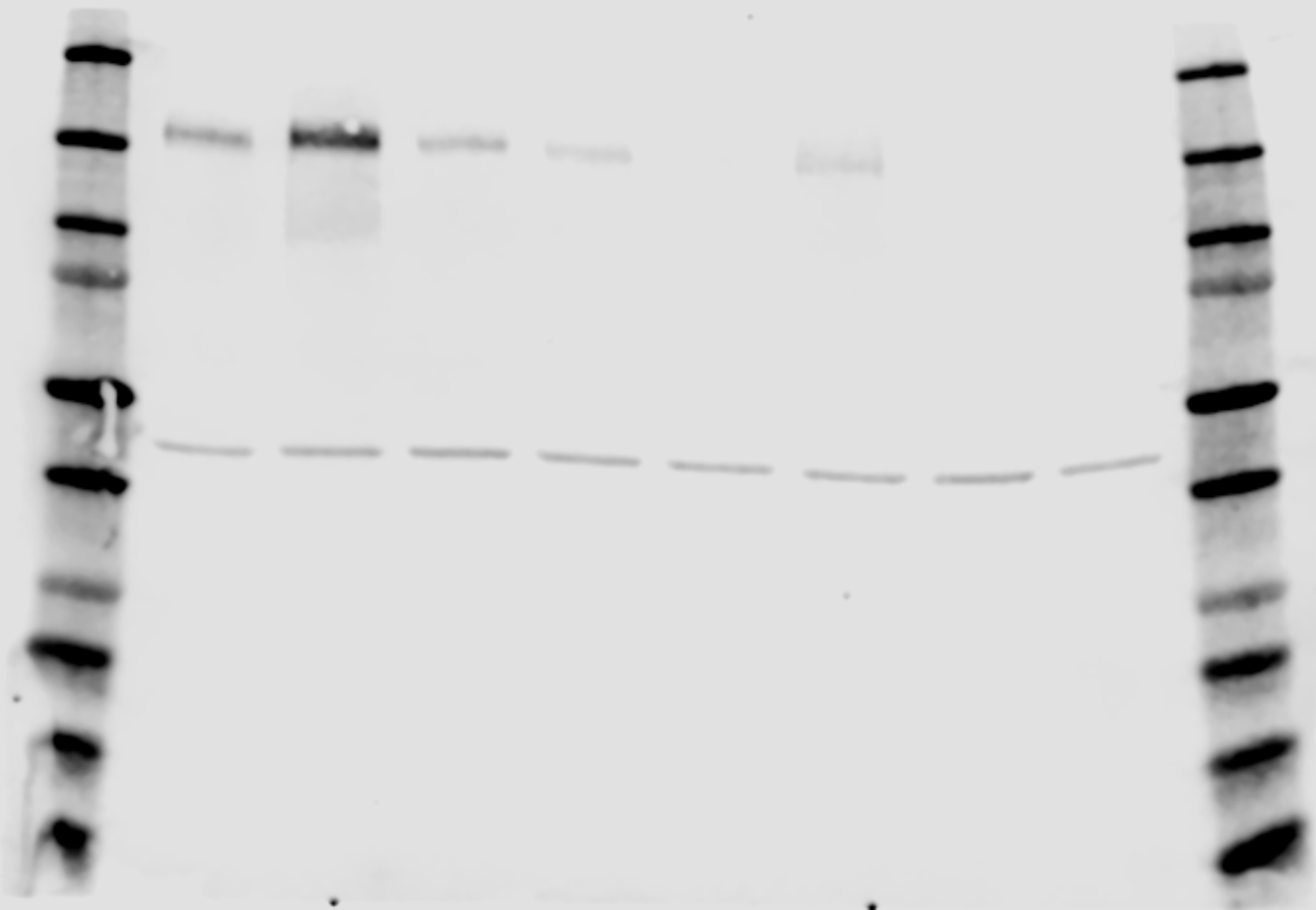

Supplement: Figure 6—figure supplement 2—source data 1. [file elife-63678-fig6-figsupp2-data1.zip › Figure 6 - Figure Supplement 2 - Source Data 1/Fig6Sup2 - EGFR - Replicate2 - PY.tif]

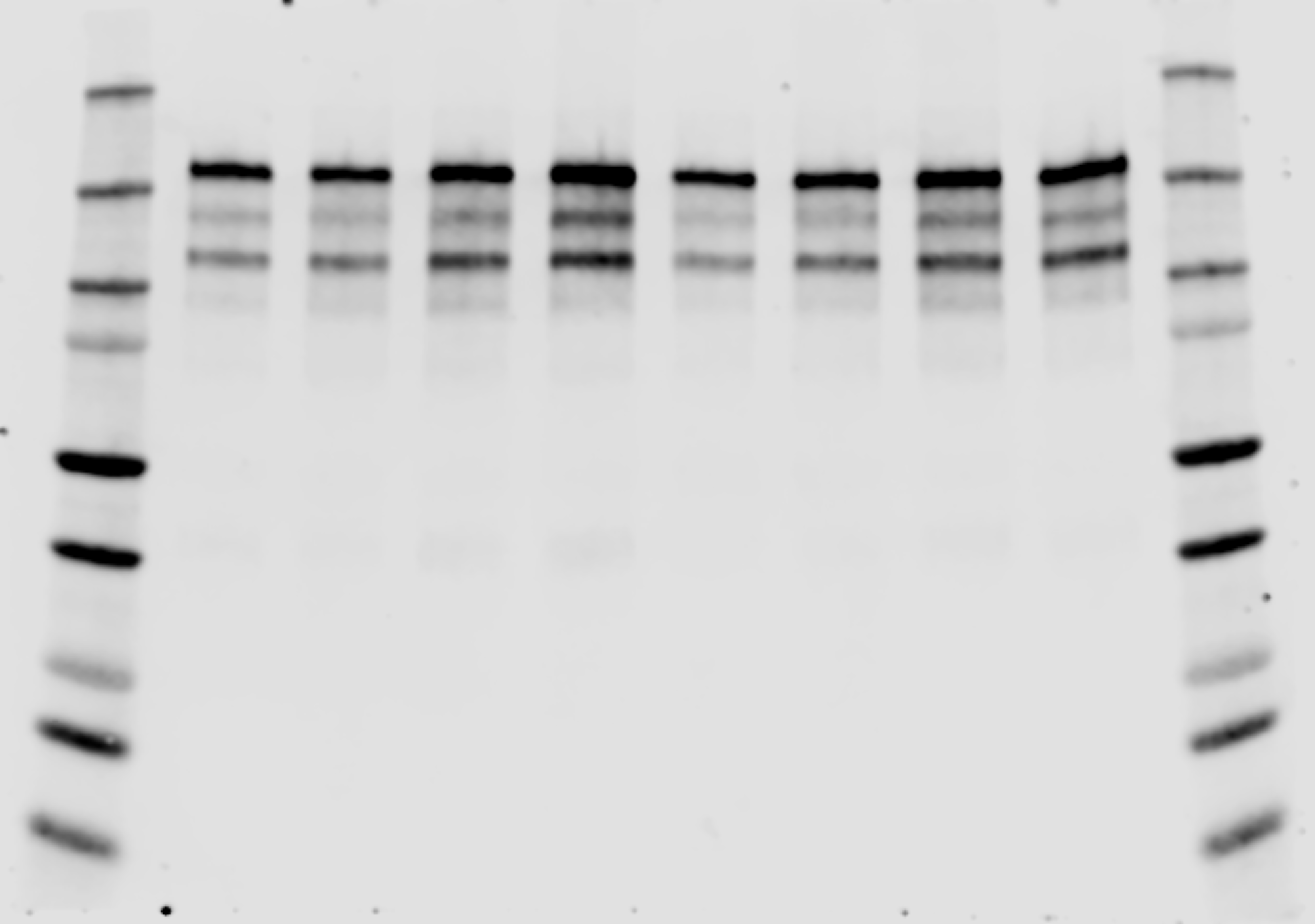

Supplement: Figure 6—figure supplement 2—source data 1. [file elife-63678-fig6-figsupp2-data1.zip › Figure 6 - Figure Supplement 2 - Source Data 1/Fig6Sup2 - RON IP - Replicate1 - Protein.tif]

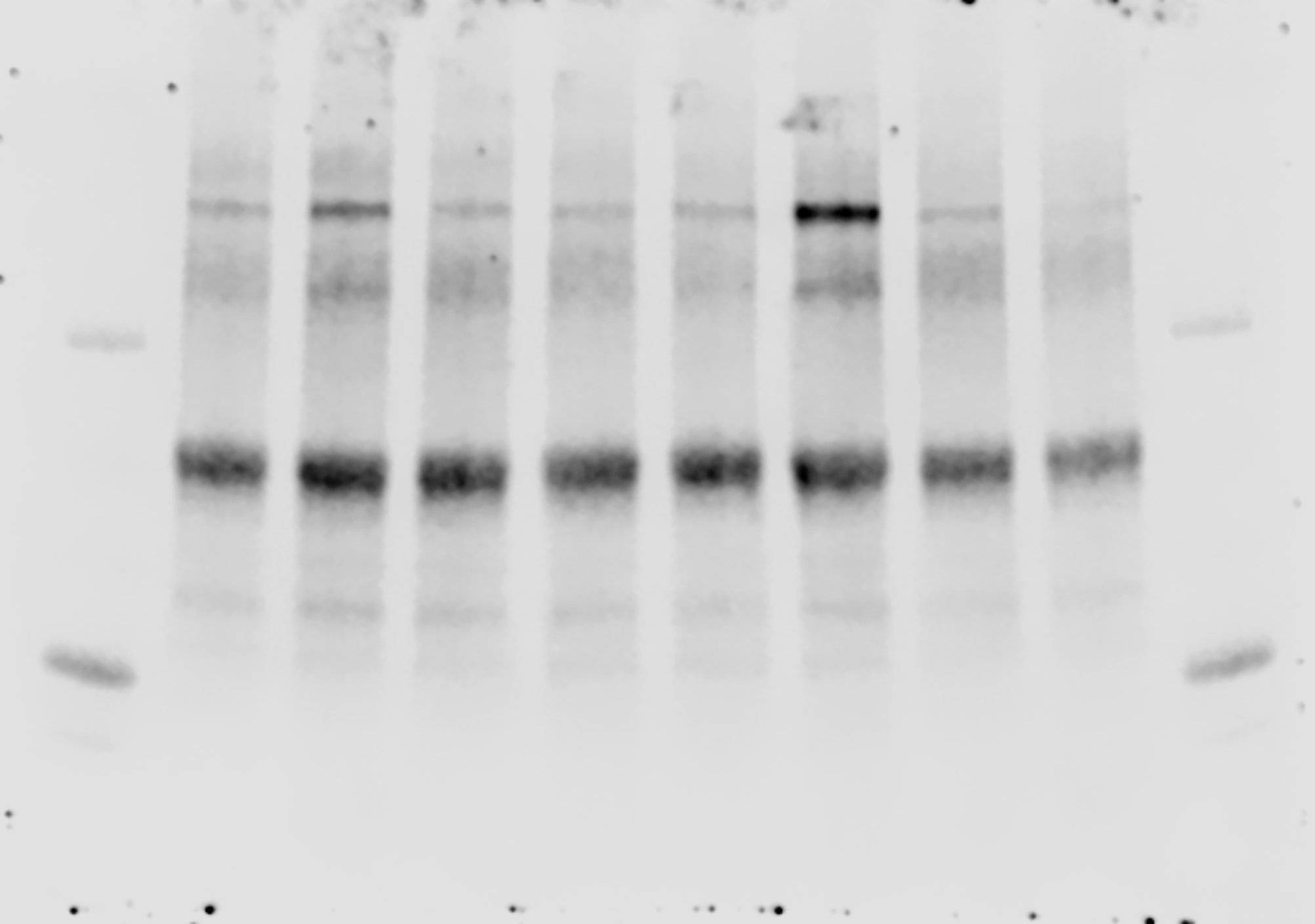

Supplement: Figure 6—figure supplement 2—source data 1. [file elife-63678-fig6-figsupp2-data1.zip › Figure 6 - Figure Supplement 2 - Source Data 1/Fig6Sup2 - RON IP - Replicate1 - PY.tif]

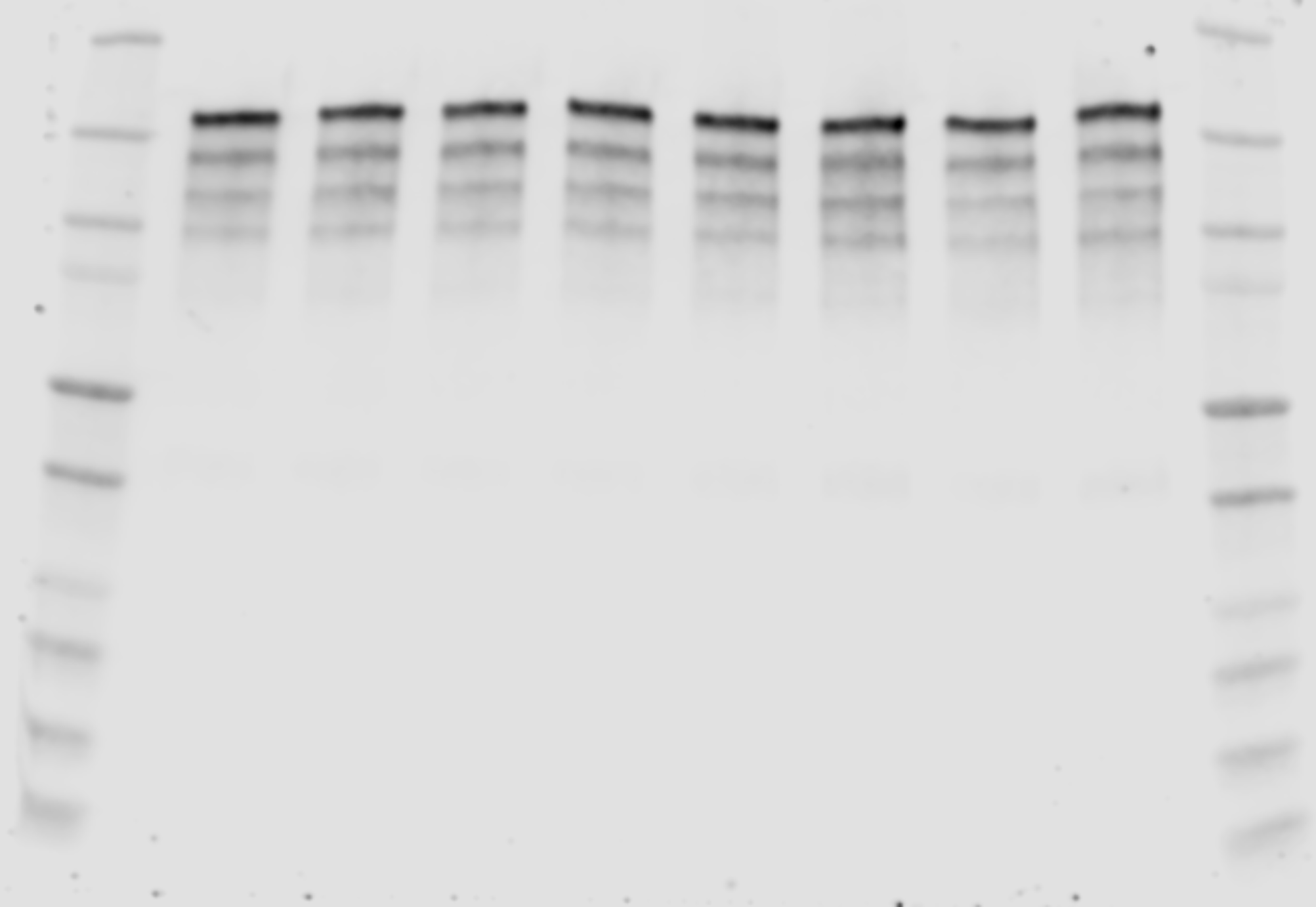

Supplement: Figure 6—figure supplement 2—source data 1. [file elife-63678-fig6-figsupp2-data1.zip › Figure 6 - Figure Supplement 2 - Source Data 1/Fig6Sup2 - RON IP - Replicate2 - Protein.tif]

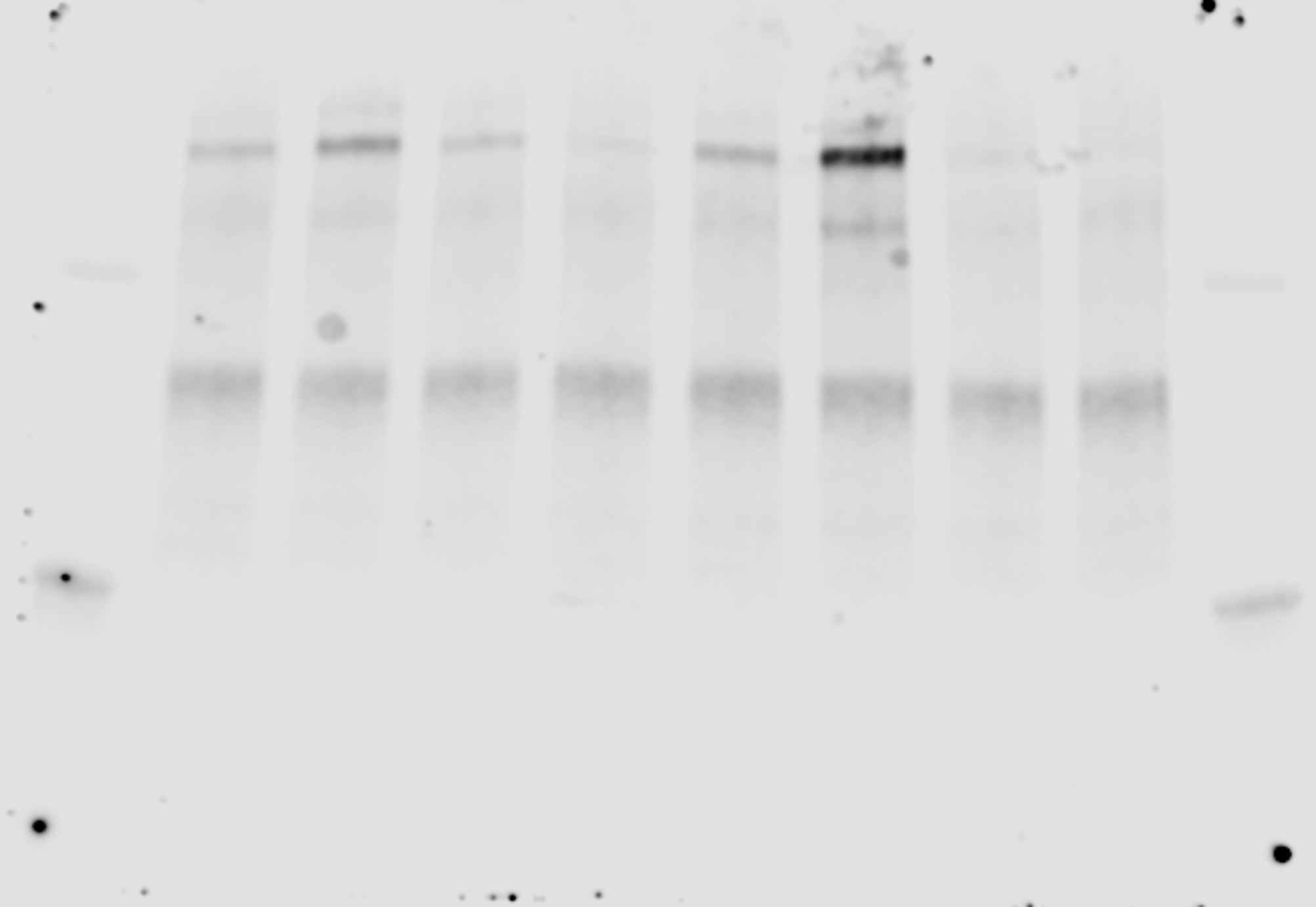

Supplement: Figure 6—figure supplement 2—source data 1. [file elife-63678-fig6-figsupp2-data1.zip › Figure 6 - Figure Supplement 2 - Source Data 1/Fig6Sup2 - RON IP - Replicate2 - PY.tif]

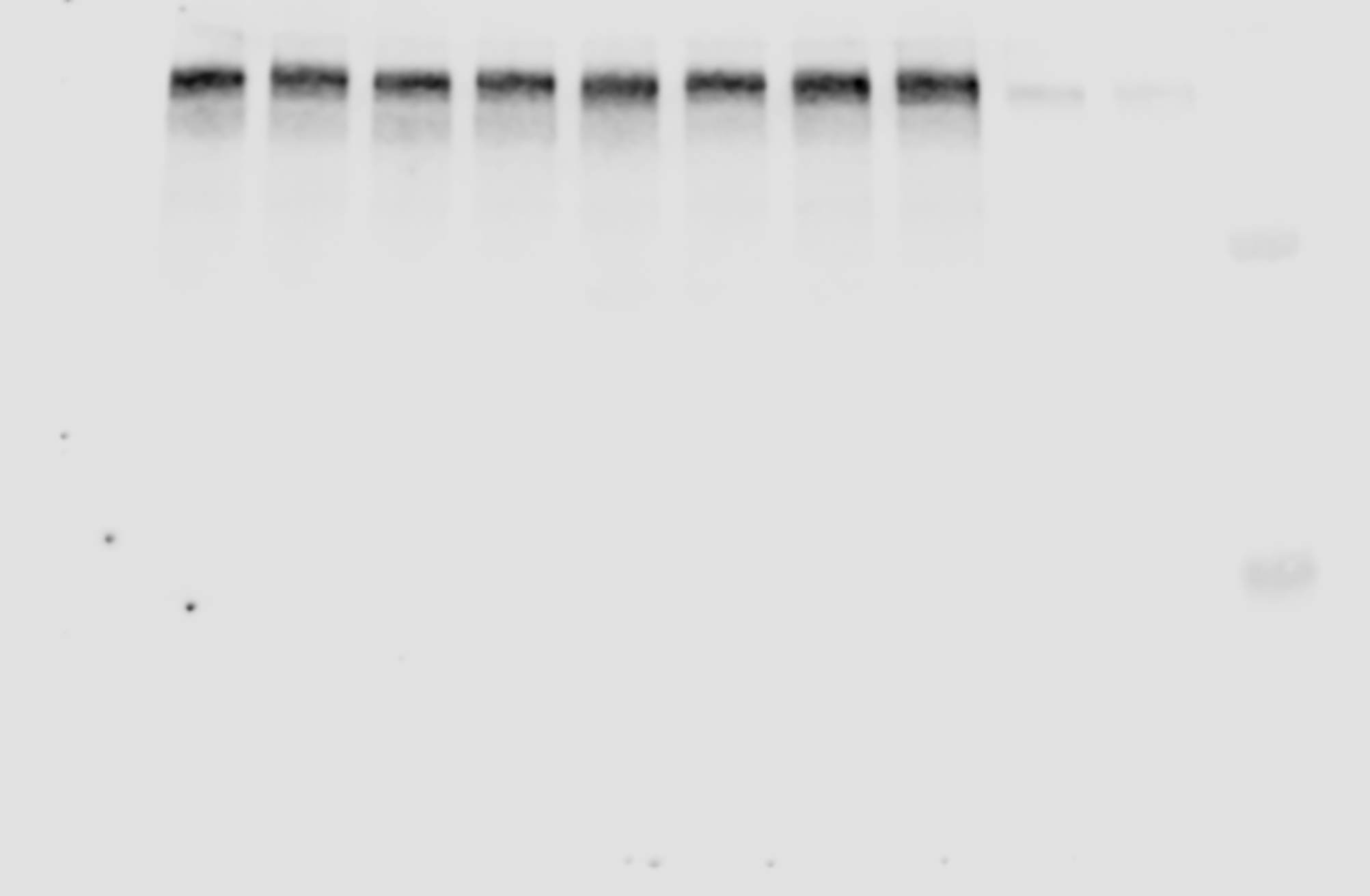

Supplement: Figure 7—source data 1. [file elife-63678-fig7-data1.zip › Figure 7 - Source Data 1/Fig7A -EGFR - Protein.tif]

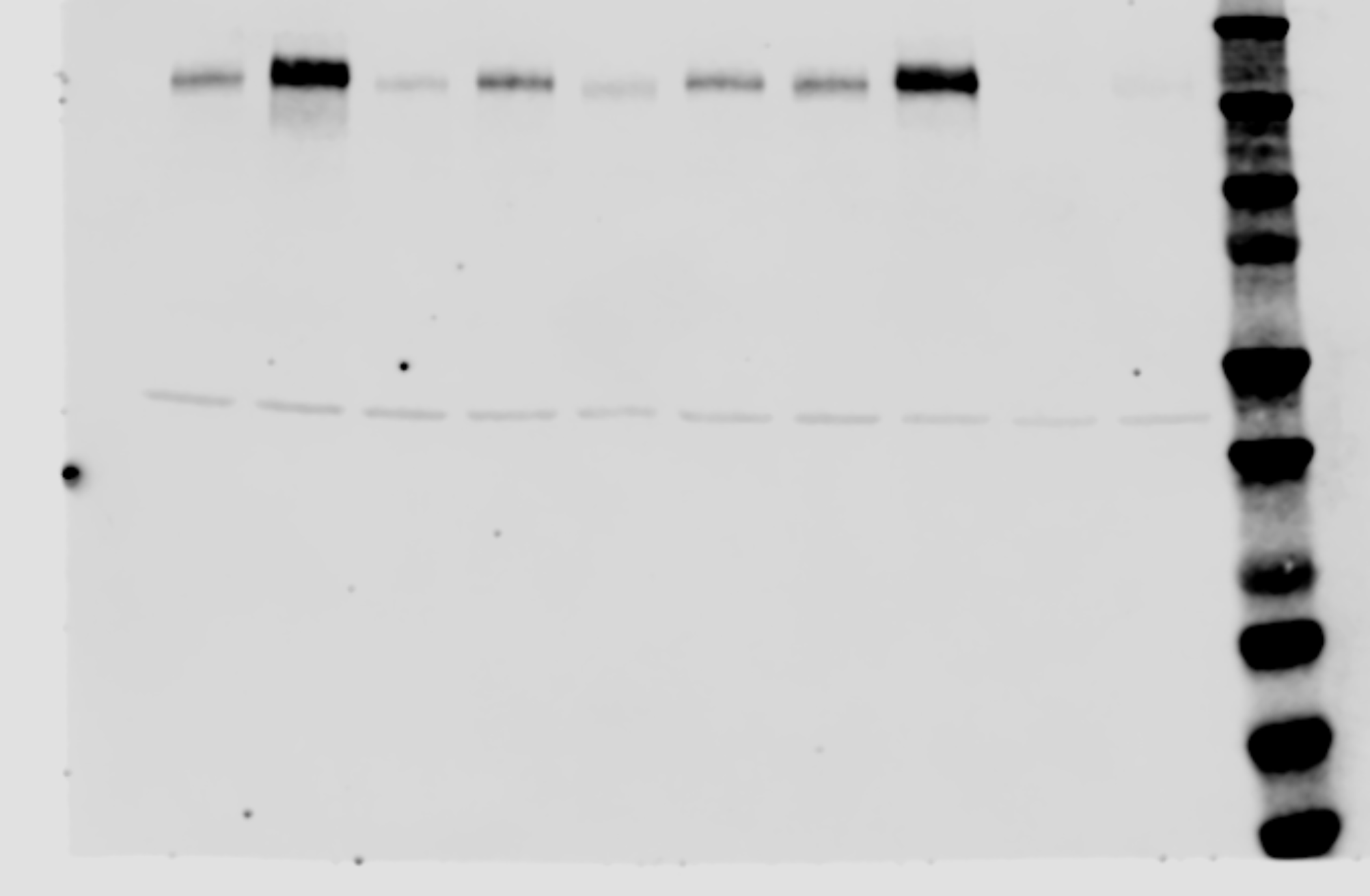

Supplement: Figure 7—source data 1. [file elife-63678-fig7-data1.zip › Figure 7 - Source Data 1/Fig7A -EGFR - PY.tif]

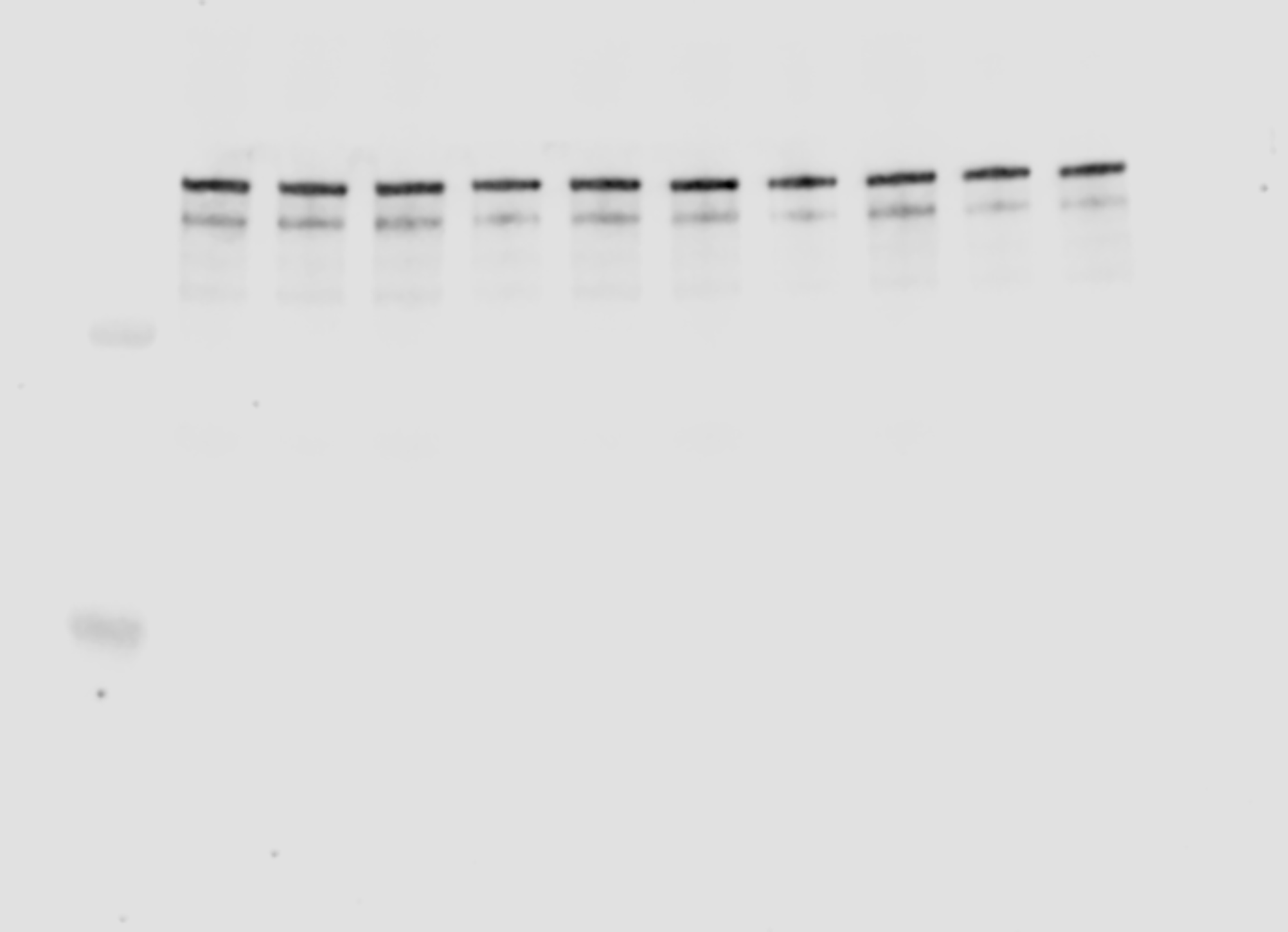

Supplement: Figure 7—source data 1. [file elife-63678-fig7-data1.zip › Figure 7 - Source Data 1/Fig7B - RON IP - Protein.tif]

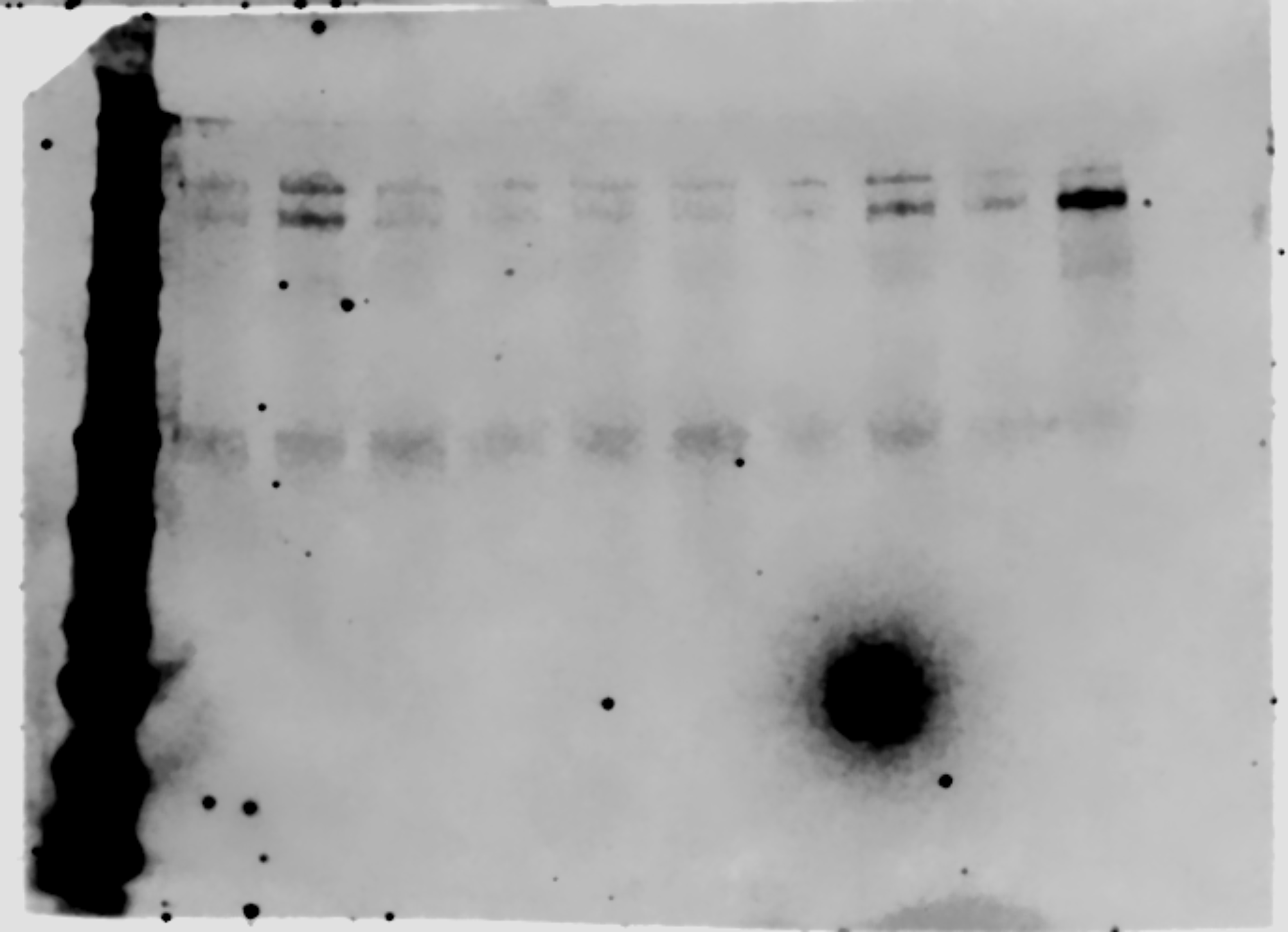

Supplement: Figure 7—source data 1. [file elife-63678-fig7-data1.zip › Figure 7 - Source Data 1/Fig7B - RON IP - PY.tif]

Figure 7 - Source Data 1

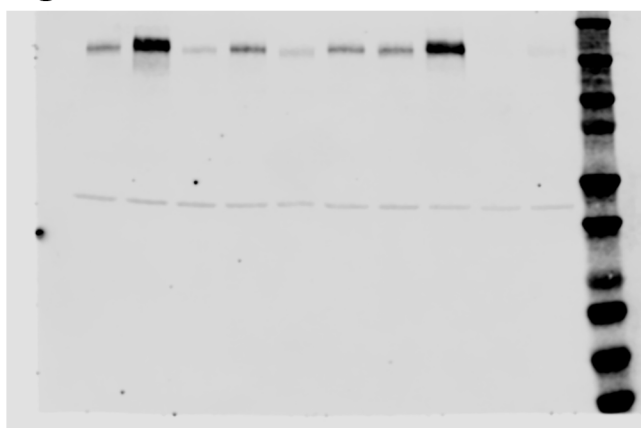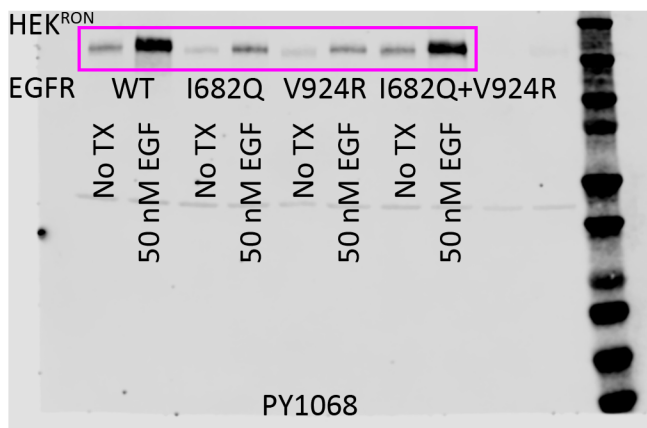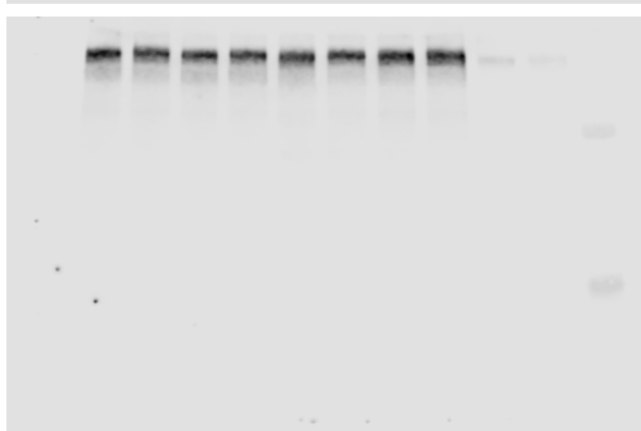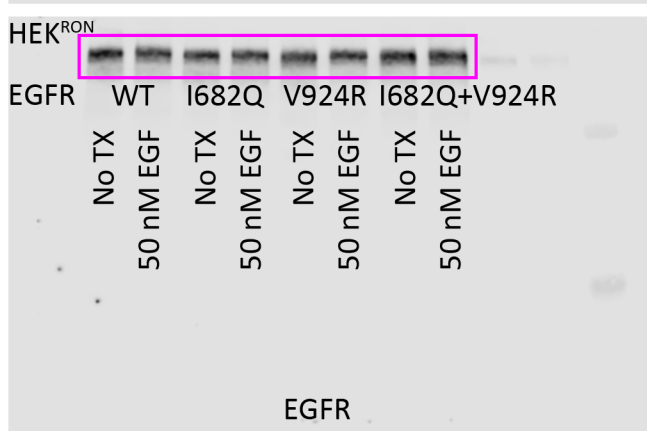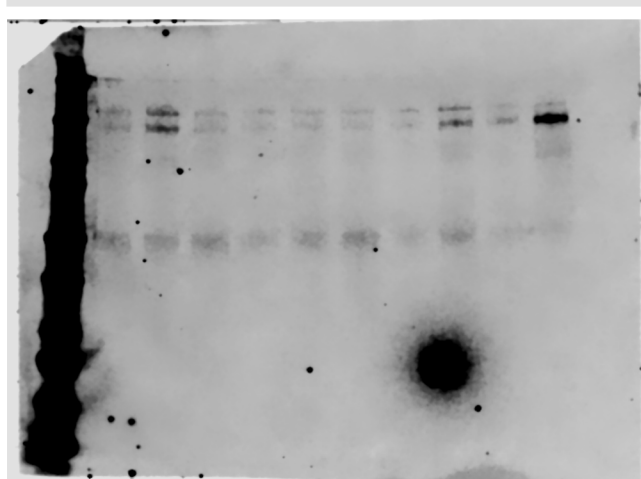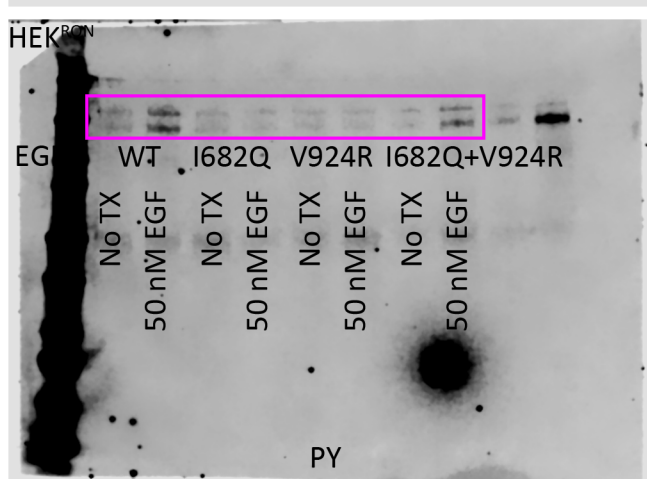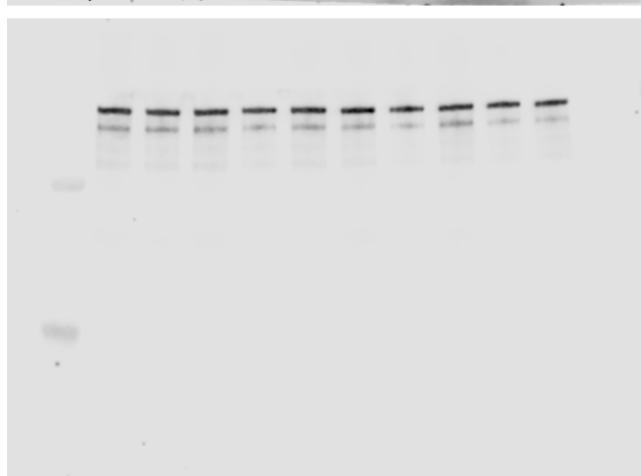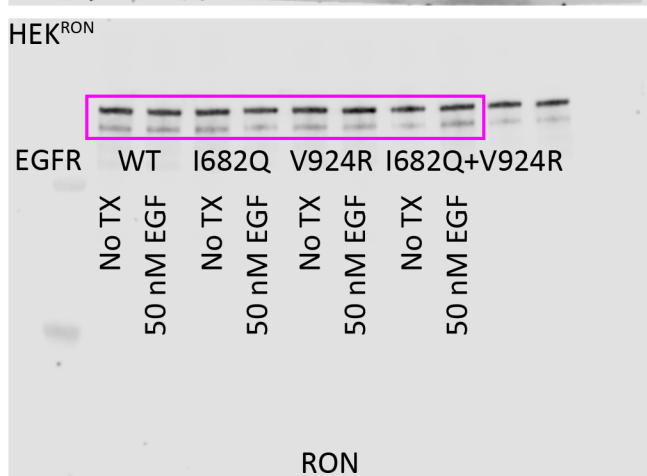

Supplement: Figure 7—source data 1. [file elife-63678-fig7-data1.zip › Figure 7 - Source Data 1/Figure 7 - Source Data 1 - Annotated.pdf]
