# Supplementary material for: EGFR transactivates RON to drive oncogenic crosstalk
Source: eLife. 2021 Nov 25;10:e63678. doi: 10.7554/eLife.63678 (PMC8654365; doi:10.7554/eLife.63678)

Figure 6 - Figure Supplement 2 - Source Data 1 - Replicate 1

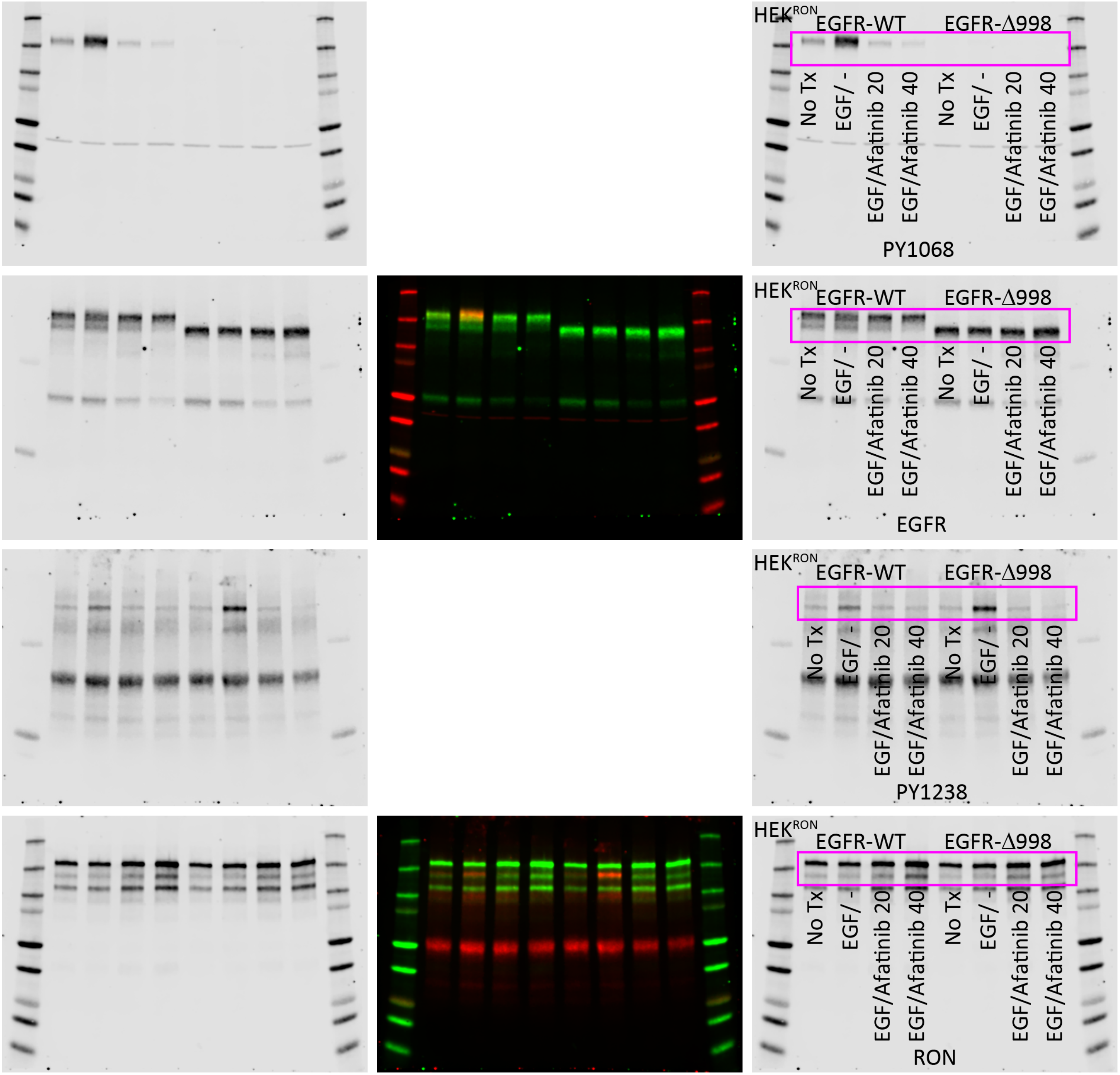

Figure 6 - Figure Supplement 2 - Source Data 1 - Replicate 2

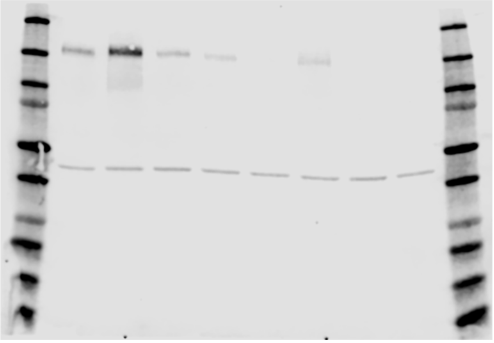

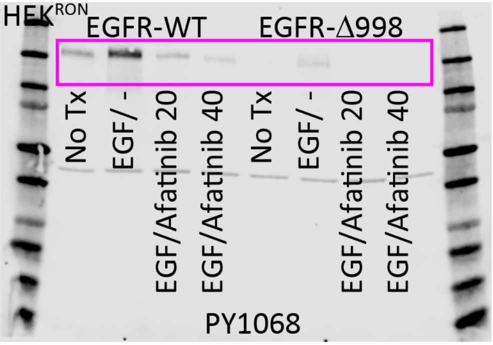

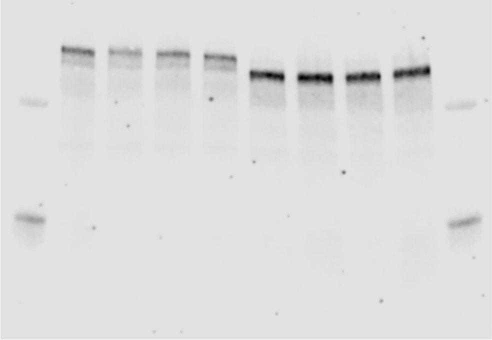

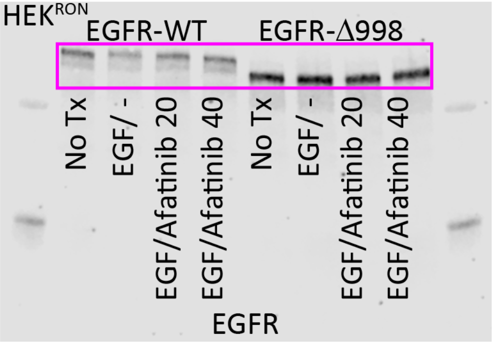

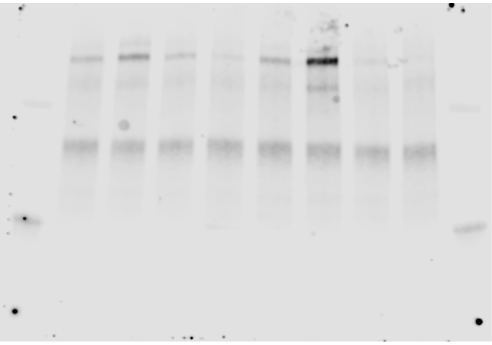

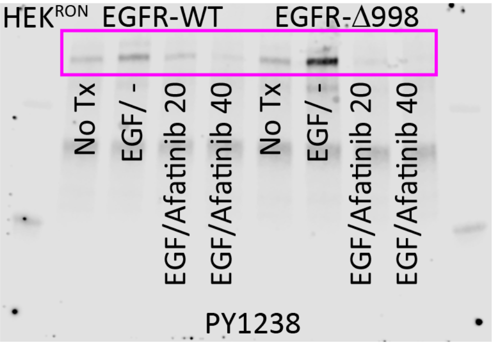

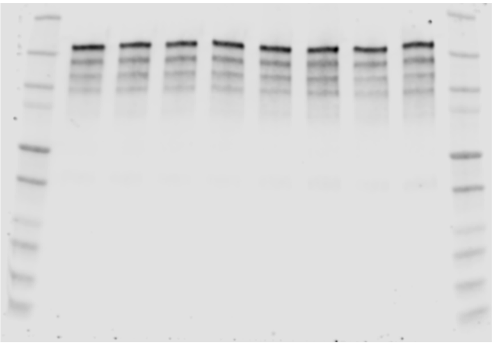

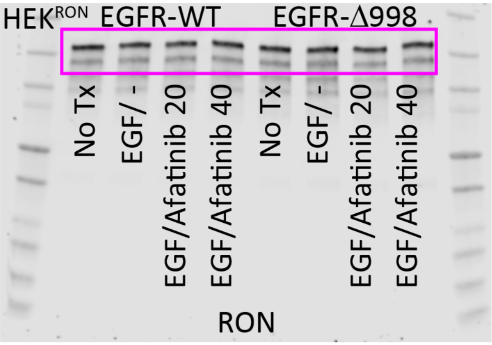

Supplement: Figure 6—figure supplement 2—source data 1. [file elife-63678-fig6-figsupp2-data1.zip › Figure 6 - Figure Supplement 2 - Source Data 1/Figure 6 - Figure Supplement 2 - Source Data 1 - Annotated.pdf]
