# Supplementary material for: EGFR transactivates RON to drive oncogenic crosstalk
Source: eLife. 2021 Nov 25;10:e63678. doi: 10.7554/eLife.63678 (PMC8654365; doi:10.7554/eLife.63678)
Supplement: Supplementary file 1. [file elife-63678-supp1.docx]

**Supplementary File 1.** List of top proteins co-IP in A431^RON^ cells with anti-HA antibody for RON pulldown, as analyzed by Mass Spectrometry.

| Gene | Description (UniProt Accession) | Molecular Weight (KDa) | Peptide Spectrum Matches | Sequence Coverage (%) | Spectral counts:  No Tx | Spectral counts:  50 nM EGF | Spectral counts:  5 nM MSP |
| --- | --- | --- | --- | --- | --- | --- | --- |
| RON | Macrophage-stimulating protein receptor (**Q04912**) | 152.6 | 1474 | 83.40 | 458.85 | 433.40 | 404.15 |
| RPB2 | DNA-directed RNA polymerase II subunit RPB2 (**P30876**) | 134.2 | 604 | 67.20 | 227.00 | 137.00 | 188.00 |
| RRP12 | RRP12-like protein (**Q5JTH9**) | 144.0 | 538 | 78.70 | 176.00 | 131.00 | 184.00 |
| MTCL1 | Microtubule cross-linking factor 1 (**Q9Y4B5**) | 210.0 | 444 | 74.60 | 153.00 | 101.00 | 153.00 |
| SOGA1 | Protein SOGA1 (**O94964**) | 160.1 | 429 | 77.90 | 136.00 | 97.00 | 152.00 |
| MA7D1 | MAP7 domain-containing protein 1 (**Q3KQU3**) | 93.0 | 390 | 78.00 | 162.00 | 59.00 | 125.00 |
| DLG5 | Disks large homolog 5 (**Q8TDM6**) | 214.3 | 358 | 67.70 | 127.00 | 75.00 | 138.00 |
| BCAR1 | Breast cancer anti-estrogen resistance protein 1 (**P56945**) | 93.6 | 328 | 88.00 | 115.50 | 63.50 | 118.50 |
| EGFR | Epidermal growth factor receptor (**P00533**) | 134.6 | 302 | 69.30 | 98.53 | 74.04 | 86.04 |
| RECQ4 | ATP-dependent DNA helicase Q4 (**O94761**) | 133.4 | 296 | 70.40 | 95.00 | 81.00 | 99.00 |
| PHLB2 | Pleckstrin homology-like domain family B member 2 (**Q86SQ0**) | 142.4 | 283 | 74.10 | 108.50 | 51.50 | 96.50 |
| CE170 | Centrosomal protein of 170 kDa (**Q5SW79**) | 175.7 | 279 | 70.90 | 106.00 | 37.50 | 97.50 |
| K2C1 | Keratin, type II cytoskeletal 1 (**P04264**) | 66.2 | 264 | 76.70 | 52.50 | 115.50 | 66.50 |
| RHG23 | Rho GTPase-activating protein 23 (**Q9P227**) | 162.5 | 253 | 69.40 | 90.50 | 58.50 | 87.00 |
| UACA | Uveal autoantigen with coiled-coil domains and ankyrin repeats (**Q9BZF9**) | 162.8 | 253 | 68.10 | 95.58 | 25.58 | 103.58 |
| KIF14 | Kinesin-like protein KIF14 (**Q15058**) | 186.9 | 253 | 62.40 | 91.00 | 57.00 | 95.00 |
| PLEC | Plectin (**Q15149**) | 532.9 | 251 | 32.90 | 101.00 | 24.50 | 106.50 |
| PKHA7 | Pleckstrin homology domain-containing family A member 7 (**Q6IQ23**) | 127.4 | 245 | 73.40 | 94.00 | 51.00 | 81.00 |
| LAMC2 | Laminin subunit gamma-2 (**Q13753**) | 131.2 | 242 | 65.80 | 81.00 | 71.00 | 79.00 |
| PKHA5 | Pleckstrin homology domain-containing family A member 5 (**Q9HAU0**) | 127.7 | 241 | 79.40 | 95.00 | 52.00 | 81.00 |
| RHG32 | Rho GTPase-activating protein 32 (**A7KAX9**) | 231.0 | 241 | 61.10 | 96.99 | 39.99 | 87.00 |
| A0A0U1RQF3 | Uncharacterized protein (**A0A0U1RQF3**) | 118.9 | 233 | 77.00 | 82.00 | 55.00 | 80.00 |
| CLAP2 | CLIP-associating protein 2 (**O75122**) | 141.4 | 226 | 67.20 | 77.50 | 52.50 | 72.50 |
| PTN14 | Tyrosine-protein phosphatase non-receptor type 14 (**Q15678**) | 135.5 | 221 | 59.10 | 79.50 | 46.50 | 71.50 |
| K1C9 | Keratin, type I cytoskeletal 9 (**P35527**) | 62.2 | 221 | 96.60 | 48.00 | 95.00 | 56.00 |
| PKCB1 | Protein kinase C-binding protein 1 (**Q9ULU4**) | 132.0 | 218 | 60.20 | 81.00 | 42.00 | 80.00 |
| KIF7 | Kinesin-like protein KIF7 (**Q2M1P5**) | 150.9 | 218 | 65.30 | 84.99 | 48.99 | 77.99 |
| CLH1 | Clathrin heavy chain 1 (**Q00610**) | 192.0 | 212 | 64.50 | 93.00 | 29.00 | 75.00 |
| SHRM3 | Protein Shroom3 (**Q8TF72**) | 217.3 | 210 | 54.50 | 82.00 | 39.00 | 69.00 |
| CAR10 | Caspase recruitment domain-containing protein 10 (**Q9BWT7**) | 116.2 | 209 | 74.40 | 87.99 | 41.00 | 64.99 |
| ZO2 | Tight junction protein ZO-2 (**Q9UDY2**) | 134.2 | 207 | 70.80 | 78.00 | 41.50 | 76.50 |
| MYO6 | Unconventional myosin-VI (**Q9UM54**) | 150.0 | 206 | 62.40 | 71.00 | 52.00 | 68.00 |
| IQEC1 | IQ motif and SEC7 domain-containing protein 1 (**Q6DN90**) | 108.5 | 204 | 65.30 | 75.50 | 45.00 | 67.50 |
| DDB1 | DNA damage-binding protein 1 (**Q16531**) | 127.2 | 202 | 61.30 | 78.00 | 50.00 | 57.00 |
| CLAP1 | CLIP-associating protein 1 (**Q7Z460**) | 169.8 | 201 | 61.40 | 63.50 | 48.50 | 70.50 |
| C170B | Centrosomal protein of 170 kDa protein B (**Q9Y4F5**) | 172.1 | 201 | 67.80 | 74.00 | 36.00 | 73.50 |
| ZN316 | Zinc finger protein 316 (**A6NFI3**) | 108.7 | 200 | 66.80 | 65.67 | 48.69 | 65.67 |
